# Research Poster Abstracts

**DOI:** 10.1080/24740527.2017.1329323

**Published:** 2017-05-22

## A controlled study of the presence of upper cervical dysfunction in concussion and whiplash patients

Howard Vernons^a^, John Crawford^a^, and Lauren Ercalao^a^

^a^Research, Canadian Memorial Chiropractic College, Toronto, Ontario, Canada

**CONTACT** Howard Vernon hvernon@cmcc.ca

Introduction/Aim: Concussions or mild traumatic brain injuries (mTBI) resulting from sports-related or whiplash injuries are a major public health issue. The extent to which neck soft tissue injuries co-occur in concussion events is not well-known nor is the extent to which the symptoms associated with these injuries contribute to post-concussion syndrome.

Methods: 43 non-acute subjects were studied: concussion (16), whiplash (13) or controls (14). Subjects completed a pain scale (VAS), the Neck Disability Index (NDI) and the Rivermead Post-Concussion Symptoms Questionnaire (RPCSQ). Three tests for cervical joint and myofascial dysfunction were conducted: joint restriction on manual palpation; tenderness to manual palpation and the Flexion-Rotation test (F-R test). Scores on all variables were compared by ANOVA or Chi-Squared testing with significance at p = 0.05.

Results: 73% of concussion subjects had head impact; none occurred in the whiplash group. In both clinical groups, the pain VAS, NDI and RPCSQ scores were not statistically different. Significantly more upper cervical joint restrictions, tenderness and positive F-R tests were found in the concussion group vs. controls. Joint restriction and F-R test findings were similar between concussion and whiplash groups.

Discussion/Conclusions: Our results provide preliminary support for similarity of symptoms and signs in both concussion and whiplash patients as well as similarly high levels of cervical joint and myofascial dysfunction in concussion subjects. Soft tissue injuries to the upper cervical spine should be assessed as early as possible in post-concussion management.

## Exploring the ecological validity of a new measure of activity-related pain using qualitative methods

Karla Jacobsen^a^, Christine Kalyn^a^, Gabriela Regalado^a^, Shabnam Taherian^a^, Ashlea Watkin^a^, Laurence Roy^a^, Jordan Miller^b^, and Timothy Wideman^a^

^a^Physical and Occupational Therapy, McGill University, Montreal, Quebec, Canada; ^b^Physical Therapy, Queen’s University, Kingston, Ontario, Canada

**CONTACT** Karla Jacobsen karla.jacobsen@mail.mcgill.ca

© 2017 Karla Jacobsen, Christine Kalyn, Gabriela Regalado, Shabnam Taherian, Ashlea Watkin, Laurence Roy, Jordan Miller, and Timothy Wideman. Published with license by Taylor & Francis.

This is an Open Access article distributed under the terms of the Creative Commons Attribution License (http://creativecommons.org/licenses/by/3.0/), which permits unrestricted use, distribution, and reproduction in any medium, provided the original work is properly cited. The moral rights of the named author(s) have been asserted.

Introduction/Aim: Musculoskeletal chronic pain is a leading cause of healthcare utilization and disability in North America. Current clinical measures are not adequately capturing the patient’s experience and perception of pain with engagement in physical activity. Increased sensitivity to physical activity (SPA) may explain low patient adherence to activity-based rehabilitation. SPA is measured through monitoring changes in pain during brief physical tasks (e.g. lifting, walking). This study aims to explore the ecological validity of a newly developed SPA measure by exploring participants’ perspectives on how the lab-based task compares to daily pain experiences.

Methods: 18 participants completed a SPA lifting task in a laboratory. Using a constructivist interpretive description methodology, semi-structured interviews were conducted to explore how experiences during this task compared and contrasted with daily experiences of activity-related pain.

Results: Similar types, patterns and intensity of pain were evoked by both the lifting task and daily physical activity. Lab-based and real life activities elicited negative emotions such as fear and anxiety related to potential of increasing pain whereas positive emotions resulted from relief and satisfaction of task completion. Differences in experiences of the lifting task and daily activities were primarily related to the lifting task parameters such as positioning, dosage, and inability to use pacing strategies.

Discussion/Conclusions: This study is a crucial step towards exploring the ecological validity of the SPA lifting task. These findings lend preliminary support for the ecological validity of these novel measures, which suggest that they may be useful clinical proxies for assessing daily activity-related pain.

## Self-management, functional status and knee-related quality of life in older adults with mid to late-stage osteoarthritis

Alix Cagnin^a^, Manon Choinière^b^, Nathalie Bureau^c^, Laetitia De Polo^c^, Hichem Saidi^b^, and Nicola Hagemeister^d^

^a^Génie concentration Technologies de la Santé, École de Technologie Supérieure (ÉTS), Montréal, Québec, Canada; ^b^Evaluation, systèmes de soins et service, Centre de Recherche du CHUM (CRCHUM), Montréal, Québec, Canada; ^c^Imagerie et Ingénierie, Centre de Recherche du CHUM (CRCHUM), Montréal, Québec, Canada; ^d^Imagerie et Ingénierie, École de Technologie Supérieure (ÉTS), Centre de Recherche du CHUM (CRCHUM), Montréal, Québec, Canada

**CONTACT** Alix Cagnin alix.cagnin.1@ens.etsmtl.ca

© 2017 Ecole de Technologie Superieure. Published with license by Taylor & Francis.

This is an Open Access article distributed under the terms of the Creative Commons Attribution License (http://creativecommons.org/licenses/by/3.0/), which permits unrestricted use, distribution, and reproduction in any medium, provided the original work is properly cited. The moral rights of the named author(s) have been asserted.

Introduction/Aim: The aim of this study was to investigate the associations between self-management perceived ability, functional status and quality of life (QOL) in patients with mid to late-stage knee osteoarthritis (OA).

Methods: Patients were selected for participation in this study if 1) they rated their worst pain in the past 7 days ≥ 4 on a 0-10 pain intensity scale, 2) had a Kellgren-Lawrence (KL) OA grade ≥ 2 on radiographs. The patients’ self-management perceived competency was measured with the Partners in Health Scale (PIHS) and the Knee Injury and Osteoarthritis Outcome Score (KOOS) was also used.

Results: There were 207 patients from 30 clinics. 60.9% were women, the mean age was 62.8 years (standard deviation SD: 9.5). More than two-thirds had an OA KL-grade of 3 (37.2%) or 4 (31.4%). PIHS scores were relatively high (mean: 79/96, SD: 12.6) and mean scores on the KOOS subscales were the following: Symptoms (61.9/100; SD: 18.3), Pain (59.4; 18.7), Activity of Daily Living (65.8; 19.5), Sports/Recreation Function (36.8; 25.9) and QOL (49.6; 24.1). PIHS scores were significantly correlated with all KOOS subscales (0.24 < r < 0.34; all p < 0.01).

Discussion/Conclusions: The more competent patients felt in their ability for self-management, the better their condition was. However, this perceived ability explained only a small proportion of the variation in their symptomatology. This suggests that there is still a lot of room for education to these patients for improving their self-care management, especially through exercise programs that target functional improvements.

## Chart audit investigating high frequency emergency department users, their health demographics for chronic pain and practice patterns of opioid provision

Bryan MacLeod^a^, Patricia Poulin^b^, Catherine Smyth^c^, Yaadwinder Shergill^d^, Dave Savage^a^, Bayley Ostenfeldt^e^, Josh Burley^a^, and Samantha Biggs^a^

^a^Northern Ontario School of Medicine, Lakehead University, Thunder Bay, ON, Canada; ^b^The Pain Clinic, The Ottawa Hospital, Ottawa, ON, Canada; ^c^Department of Anesthesiology, The Ottawa Hospital, Ottawa, ON, Canada; ^d^Clinical Research, The Ottawa Hospital, Ottawa, ON, Canada; ^e^CERAH, Lakehead University, Thunder Bay, ON, Canada

**CONTACT** Bryan MacLeod bmacleod@nosm.ca

© 2017 Bryan MacLeod, Patricia Poulin, Catherine Smyth, Yaadwinder Shergill, Dave Savage, Bayley Ostenfeldt, Josh Burley, and Samantha Biggs. Published with license by Taylor & Francis.

This is an Open Access article distributed under the terms of the Creative Commons Attribution License (http://creativecommons.org/licenses/by/3.0/), which permits unrestricted use, distribution, and reproduction in any medium, provided the original work is properly cited.

Introduction/Aim: Chronic pain (CP) affects 1 in 5 Canadians and challenges primary care providers (PCP). Lack of community care and limited expertise access can lead to frequent, costly emergency department (ED) visits and hospital admissions. A 2012 chart review by The Ottawa Hospital (TOH) demonstrated 12.9% of high frequency users (HFU) (>12 ED visits/year) ED visits were for CP. When admitted, HFU average length-of-stay was 20 and 5 days for CP and non-CP diagnosis, respectively. This study replicates TOH protocol within Thunder Bay Regional Health Sciences Centre (TBRHSC) ED, one of Canada’s busiest with over 100,000 visits annually. The goals of this study are: provide demographic characteristics of HFU at TBRHSC ED; identify percentage of CP- HFU presenting; identify percentage of CP-HFU with a PCP; CP-HFU length of ED stay; and expense of CP-HFU for TBRHSC.

Methods: To understand CP-HFU of the ED, a random sample of 268 charts, meeting HFU criteria, from TBRHSC ED will be reviewed in a retrospective chart audit over a one year period, yielding a 13% prevalence of CP HFU of ED; 95% CI; 4% margin of error. The audit will be completed by two R2 family medicine residents. 10% of charts will be assessed for inter-observer reliability, disagreements settled by PI.

Results: Data collection to begin February 2017. These remote urban setting results will be compared to TOH findings.

Discussion/Conclusions: CP is a personal and systems burden. Clarification of reality in Canada’s busiest ED will assist in RTC phase of research Fall 2017: implementing an interprofessional care pathway.

## The best of the best in pediatric self-report of pain intensity: An updated systematic review of measure psychometric properties

Kathryn A. Birnie^a,^^b^, Chitra Lalloo^b^, Amos Hundert^b^, Cynthia Nguyen^b^, and Jennifer Stinson^a,^^b^

^a^Lawrence S. Bloomberg Faculty of Nursing, University of Toronto, Toronto, Ontario, Canada; ^b^Child Health Evaluative Sciences, Hospital for Sick Children, Toronto, Ontario, Canada

**CONTACT** Kathryn A. Birnie kathryn.birnie@sickkids.ca

© 2017 Kathryn A. Birnie, Chitra Lalloo, Amos Hundert, Cynthia Nguyen, and Jennifer Stinson. Published with license by Taylor & Francis.

This is an Open Access article distributed under the terms of the Creative Commons Attribution License (http://creativecommons.org/licenses/by/3.0/), which permits unrestricted use, distribution, and reproduction in any medium, provided the original work is properly cited. The moral rights of the named author(s) have been asserted.

Introduction/Aim: To update a systematic review (Stinson et al., 2006 *PAIN*) evaluating the psychometric properties of self-report pain intensity measures in children and adolescents. At that time, six (of 34; 18%) measures had well-established evidence of reliability and validity, and were recommended for use in pediatric clinical trials.

Methods: Medline, EMBASE, CENTRAL, PsycINFO, CINAHL, and Web of Science were searched in 2016 for publications since the last review. Eligible studies assessed psychometric properties of single-item self-report pain intensity measures in 3-18 year olds. Measures were included if they met criteria of “well-established” assessment (Cohen et al., 2008). Studies were independently rated by two coders for reliability, validity, responsiveness, interpretability, and generalizability using the Consensus-based Standards for the selection of health Measurement Instruments (COSMIN) checklist (Mokkink et al., 2006).

Results: Of >12,000 abstracts screened, 35 new studies met inclusion and reported on 5 unique measures. They provided psychometric evidence for paper and electronic presentations of the Faces Pain Scale-Revised (FPS-R), Visual Analogue Scale (VAS), Wong Baker FACES Pain Scale (paper only), the 11-point Numeric Rating Scale (NRS), and the Color Analogue Scale (CAS). No new studies examined the Oucher Scale, Pieces of Hurt Tool, or the original Faces Pain Scale. Four new measures were excluded for not meeting Cohen’s criteria of well-established assessment. COSMIN ratings for reliability, validity, and responsiveness ranged from poor to excellent.

Discussion/Conclusions: Evidence supports two additional measures as psychometrically sound for self-report of pain intensity in children (NRS and CAS), and electronic versions of previously well-established measures.

## Inter-rater agreement of disability evaluation: A systematic review of reproducibility studies

Jürgen Barth^a^, Wout E.L. de Boer^a^, Jason W. Busse^b^, Jan L. Hoving^c^, Sarah Kedzia^a^, Rachel Couban^d^, Katrin Fischer^e^, David von Allmen^a^, Jerry Spanjer^f^, and Regina Kunz^a^

^a^Department of Clinical Research, University of Basel, Basel, Switzerland; ^b^Anesthesia, McMaster University, Hamilton, Ontario, Canada; ^c^Coronel Institute of Occupational Health, University of Amsterdam, Amsterdam, The Netherlands; ^d^Michael G. DeGroote Institute for Pain Research and Care, McMaster University, Hamilton, Ontario, Canada; ^e^School of Applied Psychology, Institute Humans in Complex Systems, Olten, Switzerland; ^f^Health Sciences, University Medical Center Groningen, Groningen, The Netherlands

**CONTACT** Jürgen Barth mail@juergen-barth.de

© 2017 Jürgen Barth, Wout E.L. de Boer, Jason W. Busse, Jan L. Hoving, Sarah Kedzia, Rachel Couban, Katrin Fischer, David von Allmen, Jerry Spanjer, and Regina Kunz. Published with license by Taylor & Francis.

This is an Open Access article distributed under the terms of the Creative Commons Attribution License (http://creativecommons.org/licenses/by/3.0/), which permits unrestricted use, distribution, and reproduction in any medium, provided the original work is properly cited. The moral rights of the named author(s) have been asserted.

Introduction/Aim: To explore agreement between healthcare professionals adjudicating eligibility for work disability benefits.

Methods: We reviewed MEDLINE, Embase and PsycINFO up to 16 March 2016. Observational studies investigating the reproducibility between healthcare professionals performing disability evaluations using a global rating of working capacity, and reporting inter-rater reliability by a statistical measure or descriptively were eligible for review. Study conduct may occur in insurance settings where decision on work ability include normative judgements, or in research settings where decisions on work ability disregard normative considerations. Teams of paired reviewers identified eligible studies, appraised their methodological quality and generalizability, and abstracted results, using pretested forms.

Results: Of 4562 references, 16 studies conducted in an insurance setting and 7 in a research setting, performed in 12 countries, met our inclusion criteria. Applicability of findings from studies conducted in an insurance setting to real life evaluations ranged from generalizable (n = 7, 44%), probably generalizable (n = 3, 19%) to probably not generalizable (n = 6, 37%). Most studies (n = 9, 56%) found poor inter-rater reliability, and only 13% (n = 2) reported excellent agreement. This contrasts with studies conducted in a research setting where 71% (5 of 7) achieved excellent inter-rater reliability. Reliability between assessing professionals was higher when the evaluation process was guided by a standardized instrument (23 studies, p = 0.006).

Discussion/Conclusions: Despite their common use and far-reaching consequences for workers claiming disabling injury or illness, research on the reliability of medical evaluations of work disability is very limited and indicates high variation in judgements between assessing professionals.

## Pharmacological treatment of neonatal abstinence syndrome: A decision model

Timothy Disher^a^, Louis Beaubien^b^, and Marsha Campbell-Yeo^a^

^a^School of Nursing, Dalhousie University, Halifax, Nova Scotia, Canada; ^b^Management, Dalhousie University, Halifax, Nova Scotia, Canada

**CONTACT** Timothy Disher tim.disher@dal.ca

© 2017 Timothy Disher, Louis Beaubien, and Marsha Campbell-Yeo. Published with license by Taylor & Francis.

This is an Open Access article distributed under the terms of the Creative Commons Attribution License (http://creativecommons.org/licenses/by/3.0/), which permits unrestricted use, distribution, and reproduction in any medium, provided the original work is properly cited. The moral rights of the named author(s) have been asserted.

Introduction/Aim: Neonatal abstinence syndrome (NAS) defines a constellation of symptoms in neonates exposed to opiates and other drugs intrapartum. While originally relatively rare, recent publications suggest rapidly increasing incidence. Often these infants require pharmacological treatment, there are several options to choose from. Head-to-head comparisons of pharmacological agents are abundant in the literature with some including measures of cost, but a decision analysis considering all possible options is absent. The aim is To create a normative decision model to guide selection of first-line pharmacological agents for neonatal abstinence syndrome, based on the current evidence.

Methods: We used information from existing published primary trials, and publicly available estimates of costs to develop a cost-effectiveness decision model to compare the cost-effectiveness morphine, methadone, buprenorphine, morphine + clonidine, phenobarbital alone, phenobarbital + morphine, and clonidine alone as first line pharmacological treatments for NAS. The analysis took the perspective of the Canadian health payer, and effectiveness was measured in days of exposure to potentially neurotoxic drug avoided.

Results: Under the base-case scenario, morphine + clonidine has an incremental cost-effectiveness ratio (ICER) of $824.16 per day of exposure to a potentially neurotoxic drug avoided compared to buprenorphine monotherapy. All other choices were dominated (more costly and less effective). Results are sensitive to the length of home treatment with adjunct therapy and the efficacy of buprenorphine.

Discussion/Conclusions: Under the base-case scenario, morphine + clonidine has an incremental cost-effectiveness ratio (ICER) of $824.16 per day of exposure to a potentially neurotoxic drug avoided compared to buprenorphine monotherapy. All other choices were dominated (more costly and less effective). Results are sensitive to the length of home treatment with adjunct therapy and the efficacy of buprenorphine.

## Validation of the sensitivity to pain traumatization scale (SPTS) in a sample of post-?cardiac surgery patients with different pain types

Claire Wicks^a^, Gabrielle Pagé^b^, Samantha Fashler^a^, Hance Clarke^c^, Vivek Rao^d^, and Joel Katz^a,^^c^

^a^Department of Psychology, York University, Toronto, Ontario, Canada; ^b^Centre de recherche du Centre hospitalier de l’Université de Montréal (CRCHUM), Montréal, Québec, Canada; ^c^Pain Research Unit, Department of Anesthesia and Pain Management, Toronto General Hospital, Toronto, Ontario, Canada; ^d^Division of Cardiovascular Surgery, Toronto General Hospital, Toronto, Ontario, Canada

**CONTACT** Claire Wicks cwicks@yorku.ca

© 2017 Claire Wicks, Gabrielle Pagé, Samantha Fashler, Hance Clarke, Vivek Rao, and Joel Katz. Published with license by Taylor & Francis.

This is an Open Access article distributed under the terms of the Creative Commons Attribution License (http://creativecommons.org/licenses/by/3.0/), which permits unrestricted use, distribution, and reproduction in any medium, provided the original work is properly cited. The moral rights of the named author(s) have been asserted.

Introduction/Aim: Chronic pain and posttraumatic stress are highly comorbid. The present study aimed to validate the 12-item Sensitivity to Pain Traumatization Scale (SPTS) in a sample of patients who underwent coronary artery bypass graft surgery (CABG) at least 6 months earlier. The SPTS measures a vulnerability to develop anxiety-related somatic, cognitive, emotional, and behavioral responses to pain the resemble features of a traumatic stress reaction.

Methods: The factor structure, reliability, and construct validity of the SPTS was evaluated in 345 CABGS patients (male = 263) and separately for chronic post-surgical pain (CPSP; *n* = 126), other chronic pain (OCP, *n* = 92), or no chronic pain (NCP, *n* = 127) subgroups. Participants completed the SPTS, PTSD Checklist (PCL-C), Hospital Anxiety and Depression Scale – depression subscale (HADS-D), and Leeds Neuropathic Symptoms and Signs scale (SLANSS).

Results: EFAs yielded a 1-factor structure for all groups that explained a large portion of the variance (52.4% for the overall sample, 54.7% for CPSP; 46.1% for OCP, and 52.2% for NCP). SPTS showed excellent reliability (overall: α = .88; CPSP: α = .90; OCP: α = .84; NCP: α = .85) and good preliminary construct validity. Mean SPTS-12 scores were significantly higher in females (*M* = 10.05, *SD* = 7.81) than in males (*M* = 7.19, *SD* = 7.21), *t*(343) = -3.078, *p* = 0.002.

Discussion/Conclusions: The SPTS is reliable and valid in a post-CABG surgery population. Future research should focus on clarifying whether sex interacts with SPTS in relation to pain after surgery.

## Identification of complex regional pain syndrome: Skin temperature asymmetry after cold pressor test

Tara Packham^a^, Joy C. MacDermid^b^, and Norm Buckley^c^

^a^Hand Therapy Clinic, Regional Rehabilitation Program, Hamilton Health Sciences, Hamilton, Ontario, Canada; ^b^School of Rehabilitation Sciences, McMaster University, Hamilton, ON, Canada; ^c^Michael G. DeGroote Institute for Pain Research and Care, McMaster University, Hamilton, ON, Canada

**CONTACT** Tara Packham packhamt@hhsc.ca

© 2017 Tara Packham, Joy C. MacDermid, and Norm Buckley. Published with license by Taylor & Francis.

This is an Open Access article distributed under the terms of the Creative Commons Attribution License (http://creativecommons.org/licenses/by/3.0/), which permits unrestricted use, distribution, and reproduction in any medium, provided the original work is properly cited. The moral rights of the named author(s) have been asserted.

Introduction/Aim: Skin temperature asymmetry (SkTA) may assist in early identification of complex regional pain syndrome (CRPS) but previous work has been limited by methodological shortcomings, including failure to account for the cutaneous nerve distribution where temperature is measured, and reliance on laboratory equipment not clinically available. Pilot work suggested a cold pressor test (CPT) provided a consistent thermoregulatory stress and might increase sensitivity/specificity of SkTA measurements generated reliably by handheld infra-red thermometers. This study investigated the sensitivity, specificity and validity of SkTA in the upper limb to identify CRPS.

Methods: Using infra-red thermometers, we evaluated SkTA over major peripheral nerve distributions in the hands before and after immersing a single foot in 5 °C water for 30 seconds. Participant groups included healthy volunteers, CRPS, known nerve injury, and hand fracture.

Results: SkTA was measured in 63 persons, 16 persons with CRPS (meeting Budapest criteria). Analysis of variance for n = 378 SkTA observations supported diagnosis, CPT and nerve distribution as significant predictors (p < 0.001) explaining 94% of the variance. Sensitivity for a >1.0°C SkTA improved to 68.8% from 43.8% post CPT, while specificity dropped from 85.1% to 76.6%.

Discussion/Conclusions: This study adds further support for the accuracy of SkTA as a diagnostic indicator of CRPS. Further precision in estimates will be gained from larger studies, which should also seek to replicate our findings for SkTA in the lower limbs.

## Cathodal transcranial direct current stimulation over the somatosensory cortex reduces heat pain perception in male healthy participants

Aurore Meugnot^a^, Camille Rouleau^b^, Pierre-Emmanuel Michon^c^, Julien Voisin^d^, and Philip Jackson^a^

^a^École de Psychologie,Université Laval, Centre interdisciplinaire de recherche en réadaptation et intégration sociale (CIRRIS), Centre de recherche de l’institut universitaire en santé mentale de Québec (CRIUSMQ), Québec, Québec, Canada; ^b^École de Psychologie Université Laval, CIRRIS, Québec, Québec, Canada; ^c^CIRRIS, CRIUSMQ, Québec, Québec, Canada; ^d^Département de Réadaptation, Université Laval, CIRRIS, Québec, Québec, Canada

**CONTACT** Aurore Meugnot aurore.meugnot.1@ulaval.ca

© 2017 Aurore Meugnot, Camille Rouleau, Pierre-Emmanuel Michon, Julien Voisin, and Philip Jackson. Published by Taylor & Francis

This is an Open Access article distributed under the terms of the Creative Commons Attribution License (http://creativecommons.org/licenses/by/3.0/), which permits unrestricted use, distribution, and reproduction in any medium, provided the original work is properly cited. The moral rights of the named author(s) have been asserted.

Introduction/Aim: Non-Invasive brain stimulation including transcranial direct current stimulation (tDCS) of the primary motor cortex could be a valuable therapeutic approach to treat neuropathic or chronic pain (Cruccu et al. 2016). Our study examined the effect of tDCS of the somatosensory cortex, a region involved in the sensory processing of pain, to modulate experimentally-induced pain perception, in both males and females as the effect of sex on brain stimulation remains poorly understood (Tommaso et al. 2014).

Methods: 18 healthy adults (10 females) participated to three sessions (at least one week apart) corresponding either to cathodal, anodal (*actives conditions*) and sham (*control condition*) tDCS. In each session, participants rated the pain level of heat stimulations (thermode) on a visual analog scale, before (pre-test) and immediatly after (post-test) receiving a 20 minute session of tDCS with either an intensity of 2 mA (active), or no current except for the first 30 seconds (sham).

Results: The ANOVA (session x sex) of pain ratings changes from pre-post tests showed that cathodal tDCS significantly diminished pain perception following painful stimulations compared to sham tDCS in males, while anodal tDCS showed no significant effect. In females, there were no significant differences between actives tDCS and sham tDCS.

Discussion/Conclusions: These findings confirm that tDCS over the somatosensory cortex significantly diminished subjective pain perception, whereas anodal and sham stimulations had no effect (Antal et al. 2008). Notably, the tDCS induced-effects in the present study were specific to males, showing the influence of sex in the relationship between brain stimulation and pain perception.

## Flurbiprofen 8.75 mg delivered as a lozenge or spray provides relief from difficulty swallowing and swollen throat

Adrian Shephard^a^, Valeria Bychkova^b^, Joanne Hunt^c^, Natalia Burova^d^, and Eugenia Radkova^e^

^a^Reckitt Benckiser Healthcare International Ltd, Slough, Berkshire, UK; ^b^Evidence Generation & Clinical Research, Reckitt Benckiser, Moscow, Russia; ^c^Respiratory Medical Science, Health Relief, Reckitt Benckiser, Slough, Berkshire, UK; ^d^Federal State Establishment Clinical Diagnostic Medical Center Department, Saint Petersburg, Russia; ^e^OCT Clinical Trials, Saint Petersburg, Russia

**CONTACT** Adrian Shephard Adrian.Shephard@rb.com

© 2017 Reckitt Benckiser Healthcare International Ltd. Published with license by Taylor & Francis.

This is an Open Access article distributed under the terms of the Creative Commons Attribution License (http://creativecommons.org/licenses/by/3.0/), which permits unrestricted use, distribution, and reproduction in any medium, provided the original work is properly cited. The moral rights of the named author(s) have been asserted.

Introduction/Aim: Patients describe their sore throat using various descriptors such as difficult to swallow, or the sensation of swollen throat. Flurbiprofen has been developed as a spray and lozenge to treat the symptoms of sore throat. The aim of this study was to investigate the effect of flurbiprofen 8.75 mg, delivered as a spray or lozenge, on difficulty swallowing and swollen throat.

Methods: A randomized, double-blind study was conducted at 16 sites across Russia. Adults with acute sore throat were randomly assigned to take one dose of flurbiprofen 8.75 mg spray plus a placebo lozenge (n = 218), or flurbiprofen 8.75 mg lozenge plus placebo spray (n = 222). Patients rated difficulty swallowing and swollen throat using 100-mm visual analogue scales: Difficulty Swallowing Scale (DSS, ‘not difficult’ to ‘very difficult’) and Swollen Throat Scale (SwoTS, ‘not swollen’ to ‘very swollen’).

Results: In the per-protocol set (n = 417), patients in both groups reported similar reductions in DSS and SwoTS. The LS mean change from baseline in DSS was -33.39 (spray) and -31.69 (lozenge) at 1 hour (p = 0.2694) and -36.53 (spray) and -36.04 (lozenge) at 2 hours (p = 0.7707). The LS mean change from baseline in SwoTS was -26.64 (spray) and -25.57 (lozenge) at 1 hour (p = 0.4431) and -30.51 (spray) and -29.48 (lozenge) at 2 hours (p = 0.4978). Similar results were found in the full analysis set (n = 439).

Discussion/Conclusions: Flurbiprofen lozenge and spray both provide an equivalent and meaningful reduction in difficulty swallowing and swollen throat, providing patients with effective treatment regardless of how they describe their symptoms.

## Predictors of chronic widespread pain or fibromyalgia: A systematic review and meta-analysis of observational studies

Yasir Rehman^a^, Nadia Rehman^b^, Eric Chen^c^, Augustine Toma^c^, Diane Heels-Ansdell^a^, Rachel Couban^d^, Gordon Guyatt^a^, and Jason Busse^d^

^a^Health Research Methods, Evidence and Impact, McMaster University, Hamilton, ON, Canada; ^b^Nursing, McMaster University, Hamilton, Ontario, Canada; ^c^Medicine, McMaster University, Hamilton, Ontario, Canada; ^d^Anesthesia, McMaster University, Hamilton, Ontario, Canada

**CONTACT** Yasir Rehman dry_rehman@yahoo.ca

© 2017 Yasir Rehman, Nadia Rehman, Eric Chen, Augustine Toma, Diane Heels-Ansdell, Rachel Couban, Gordon Guyatt, and Jason Busse. Published with license by Taylor & Francis.

This is an Open Access article distributed under the terms of the Creative Commons Attribution License (http://creativecommons.org/licenses/by/3.0/), which permits unrestricted use, distribution, and reproduction in any medium, provided the original work is properly cited. The moral rights of the named author(s) have been asserted.

Introduction/Aim: To systematically explore factors associated with the development of chronic widespread pain (CWSP) or fibromyalgia (FM).

Methods: We searched MEDLINE, EMBASE, PsychINFO and Pubmed from inception to December 2016, to identify cohort or case-control studies that reported the association between risk factors and CWSP or FM in an adjusted model. Literature screening, data extraction and risk of bias were completed, independently and in duplicate, by teams of reviewers. We pooled estimates of association using random effects models, when possible, for all independent variables reported by >1 study. We report relative measures of association as pooled odds ratios (ORs) and associated 95% confidence intervals (95% CIs).

Results: Our search identified 23 eligible studies with 86,220 participants. High quality evidence demonstrated an increased odds of CWSP or FM with being employed (OR = 3.15, 95%CI 1.52 to 6.54), a family history of pain (OR = 2.66, 95%CI 1.63 to 4.30), illness behavior (OR = 2.96, 95%CI 2.10 to 4.16), multi-site pain not meeting criteria for CWSP or FM (OR = 3.18, 95%CI 1.25 to 8.10), female gender (OR = 1.15, 95%CI 1.01 to 1.32), current smoking (OR = 1.31, 95%CI 1.07 to 1.60), obesity (OR = 1.34, 95%CI 1.15 to 1.56), and anxiety (OR = 1.24, 95%CI 1.07 to 1.44). High quality evidence demonstrated no association with former smoking, and low quality evidence found no association with depression, cognitive deficits, or stressful life events.

Discussion/Conclusions: Our review identified several risk factors for the development of CWSP or FM that may be helpful for targeting high-risk individuals for preventative management strategies.

## Attitudes toward the possibility of an online pain assessment/management training program in rural long-term care facilities

Natasha L. Gallant^a^, Thomas Hadjistavropoulos^a^, and Abigail Wickson-Griffiths^b^

^a^Psychology, University of Regina, Regina, Saskatchewan, Canada; ^b^Nursing, University of Regina, Regina, Saskatchewan, Canada

**CONTACT** Natasha L. Gallant gallanat@uregina.ca

© 2017 Natasha L. Gallant, Thomas Hadjistavropoulos, and Abigail Wickson-Griffiths. Published with license by Taylor & Francis.

This is an Open Access article distributed under the terms of the Creative Commons Attribution License (http://creativecommons.org/licenses/by/3.0/), which permits unrestricted use, distribution, and reproduction in any medium, provided the original work is properly cited. The moral rights of the named author(s) have been asserted.

Introduction/Aim: Underassessment of pain remains a significant issue for residents living in long-term care facilities. In rural settings, pain assessment is complicated by insufficient continuing education opportunities due to geographical isolation. The goal of this project was to evaluate, using qualitative methods, the readiness of rural facilities to address inadequacies in continuing education through online training on state-of-the-art pain assessment practices.

Methods: We conducted semi-structured interviews with 10 nurses, care aides, and administrators from two rural long-term care facilities. They were asked about the extent to which they perceived a need for specialized pain assessment training, barriers that were specific to the facility, and the extent to which they believed that online pain assessment training would address these barriers. Narrative data were analyzed using thematic content analysis.

Results: Staff perceived a need for a standardized pain assessment/management protocol to adequately address pain. Reservations about a standardized clinical approach to pain related to a perceived increase in documentation, time constraints, negative attitudes toward change, and potential for miscommunications among staff. Reactions to the possibility of online training were mixed. Negative reactions were related to unfamiliarity with online training and a preference for face-to-face training, whereas positive reactions were related to the belief that online training would address the lack of training opportunities in rural settings.

Discussion/Conclusions: Findings from this project will guide future implementation of online pain education in these two long-term care facilities to maximize probability of success through collaborative problem-solving.

## Cannabidiol attenuates the activity of joint nociceptors in a rat model of osteoarthritis

M. O’Brien and Jason J. McDougall

Pharmacology, Dalhousie University, Halifax, NS, Canada

**CONTACT** Melissa O’Brien melissa.obrien@dal.ca

© 2017 Melissa O’Brien and Jason J. McDougall. Published with license by Taylor & Francis.

This is an Open Access article distributed under the terms of the Creative Commons Attribution License (http://creativecommons.org/licenses/by/3.0/), which permits unrestricted use, distribution, and reproduction in any medium, provided the original work is properly cited. The moral rights of the named author(s) have been asserted.

Introduction/Aim: Cannabidiol (CBD), a non-psychoactive cannabinoid, has recently been shown to attenuate pain and inflammation in an animal model of rheumatoid arthritis, but its effect on osteoarthritis (OA) pain is unknown. This study investigated whether local administration of CBD to a mono-arthritic knee joint is capable of altering the activity of joint nociceptors.

Methods: A chronic model of OA was induced in male Wistar rats (387-463g) by intra-articular injection of sodium monoiodoacetate (MIA, 3mg/50μl). *In vivo* electrophysiology experiments were conducted in with either moderate (days 14–20) and severe (days 21–27) OA. Single unit recordings of knee joint afferents that responded to mechanical rotation of the joint were carried out. Evoked firing from non-noxious and noxious rotation of the joint before and following close intra-arterial injection of CBD (100, 200, and 300μg in DMSO:Cremophor:Saline, 1:1:8) were compared.

Results: Peripheral administration of CBD reduced noxious movement-evoked firing of knee afferent fibres in a dose-dependent manner (300 μg vs 100 μg, P < 0.005, n = 4-5). The desensitizing effect of CBD was more pronounced in fibres recorded from animals with severe OA. The 300 μg dose of CBD decreased afferent firing rate in response to noxious rotation by 13 and 19% in day 14–20 and 21–27 animals respectively (P < 0.0001, n = 3–5).

Discussion/Conclusions: This study demonstrates for the first time that local administration of CBD can reduce joint mechanonociception and merits further investigation into the potential use of CBD for OA pain.

## Adherence to the consort statement among randomized controlled trials assessing opioids for chronic non-cancer pain

Alka Kaushal^a^, Lucas Gallos^b^, Muhammad (Adeel) Akhtar^c^, Naomi Scott^d^, Mishaal Shahid Umar^e^, Vamana Rajeswaran^b^, Tamana Yousof^f^, Priya Gupta^c^, Li Wang^g^, Rachel Couban^g^, Norman Buckley^g^, Lehana Thabane^a^, and Jason W. Busse^g^

^a^Health Research Methods, Evidence and Impact, McMaster University, Hamilton, Ontario, Canada; ^b^Medicine, McMaster University, Hamilton, ON, Canada; ^c^Faculty of Health Sciences, McMaster University, Hamilton, Ontario, Canada; ^d^Midwifery, McMaster University, Hamilton, ON, Canada; ^e^Health Sciences, McMaster University, Hamilton, Ontario, Canada; ^f^Medical Sciences, McMaster University, Hamilton, Ontario, Canada; ^g^Anesthesia, McMaster University, Hamilton, Ontario, Canada

**CONTACT** Alka Kaushal kausha3@mcmaster.ca

© 2017 Alka Kaushal, Lucas Gallos, Muhammad (Adeel) Akhtar, Naomi Scott, Mishaal Shahid Umar, Vamana Rajeswaran, Tamana Yousof, Priya Gupta, Li Wang, Rachel Couban, Norman Buckley, Lehana Thabane, and Jason W. Busse. Published with license by Taylor & Francis.

This is an Open Access article distributed under the terms of the Creative Commons Attribution License (http://creativecommons.org/licenses/by/3.0/), which permits unrestricted use, distribution, and reproduction in any medium, provided the original work is properly cited. The moral rights of the named author(s) have been asserted.

Introduction/Aim: The CONSORT statement is a highly-endorsed checklist that promotes a standard approach for reporting of trial findings, facilitates complete and transparent reporting, and aids critical appraisal and interpretation. We conducted a systematic review to establish the effect of the original CONSORT statement on reporting quality among randomized controlled trials assessing opioids for chronic non-cancer pain.

Methods: We systematically searched several electronic databases for English-language studies that randomized patients with chronic non-cancer pain to receive an opioid or a non-opioid control. In duplicate and independently, teams of reviewers established the eligibility of each identified study, and recorded all CONSORT recommended items from trials that proved eligible. We conducted adjusted logistic regression analyses to explore the association between independent variables and reporting of CONSORT items. We fit one model per CONSORT item that showed sufficient variability in reporting; specifically, we did not consider items that were reported less than 10% of the time or greater than 90% of the time.

Results: Out of a total of 23,109 citations, 77 trials proved eligible. Data abstraction has been completed and the analysis is ongoing. Results will be available at the CPS Annual Scientific Meeting in May 2017.

Discussion/Conclusions: Our review will establish the concordance of reporting among trials of opioids for chronic non-cancer pain with the CONSORT statement, whether reporting quality improved after publication of the CONSORT statement, and factors associated with reporting of CONSORT items.

## Emotion regulation model of pain, depression, and disability in women with interstitial cystitis/bladder pain syndrome

Alison Crawford^a^, Dean A. Tripp^b^, J. Curtis Nickel^c^, Lesley Carr^d^, Robert Moldwin^e^, Robert Mayer^f^, Laura Katz^g^, and Abi Muere^a^

^a^Psychology, Queen’s University, Kingston, ON, Canada; ^b^Psychology, Urology, & Anesthesiology, Queen’s University, Kingston, ON, Canada; ^c^Urology, Queen’s University, Kingston, ON, Canada; ^d^Department of Surgery, University of Toronto and Sunnybrook Health Science Centre, Toronto, ON, Canada; ^e^Department of Urology, Hofstra University School of Medicine, New Hyde Park, NY, USA; ^f^Asante Physician Partners, Grants Pass, OR, USA; ^g^Michael G. DeGroote Pain Clinic, McMaster University Medical Centre, Hamilton, ON, Canada

**CONTACT** Alison Crawford alison.crawford@queensu.ca

© 2017 Alison Crawford, Dean A. Tripp, J. Curtis Nickel, Lesley Carr, Robert Moldwin, Robert Mayer, Laura Katz, and Abi Muere. Published with license by Taylor & Francis.

This is an Open Access article distributed under the terms of the Creative Commons Attribution License (http://creativecommons.org/licenses/by/3.0/), which permits unrestricted use, distribution, and reproduction in any medium, provided the original work is properly cited. The moral rights of the named author(s) have been asserted.

Introduction/Aim: Interstitial Cystitis/Bladder Pain Syndrome (IC/BPS) is a chronic pelvic pain condition characterized by pain flares localized to the bladder and by urinary urgency, frequency, and dysuria (Nickel et al., 2009). The literature has suggested that pain, catastrophizing, depression, and disability are closely linked, but investigations on exactly how they are associated remain to be conducted. The aim of this study was to test an emotion regulation model of pain, depression, and disability in an IC/BPS sample using serial mediation.

Methods: A total of 225 women diagnosed with IC/BPS recruited from tertiary care clinics in Canada and the U. S. completed questionnaires regarding demographics, pain, catastrophizing, emotion regulation, depression, and pain related disability. We ran serial mediations using Hayes’ (2013) PROCESS macro testing whether difficulties in emotion regulation mediates the indirect effect of catastrophizing on the relationship between pain and depression (model 1), as well as the relationship between pain and pain related disability (model 2).

Results: A bias-corrected bootstrap confidence interval for the indirect effect of model 1 (*b* = .07) based on 10 000 bootstrap samples was entirely above zero (.0456 to .1058) indicating a significant indirect effect. The indirect effect of model 2 (*b* = .07) was also significant (CI = .0314 to .1363).

Discussion/Conclusions: The way pain influences the experience of depression and pain-related disability may operate through one’s ability to regulate emotions, especially those related to pain. Understanding how emotion regulation affects a patient’s experience is an important step toward informing psychological management of pain.

## Does kinesiophobia increase after a spinal fusion surgery in paediatrics: Preliminary results

Diana-Luk Ye^a^, Sheila Bote^b^, Neil Saran^c^, Jean A. Ouellet^d^, and Catherine E. Ferland^e^

^a^Experimental Surgery, McGill University, Montreal, Quebec, Canada; ^b^Clinical Research – Orthopaedic Surgery, Shriners Hospital for Children-Canada, McGill Scoliosis & Spine Group, Montreal, Quebec, Canada; ^c^Pediatric Surgery, Shriners Hospital for Children-Canada, McGill University Health Centre, Montreal, Quebec, Canada; ^d^Orthopedic Surgery, Shriners Hospital for Children-Canada, McGill University Health Centre, Montreal, Quebec, Canada; ^e^Anesthesia, Shriners Hospitals for Children-Canada, McGill University Health Centre, Montreal, Quebec, Canada

**CONTACT** Diana-Luk Ye diana-luk.ye@mail.mcgill.ca

© 2017 Diana-Luk Ye, Sheila Bote, Neil Saran, Jean A. Ouellet, Catherine E. Ferland. Published with license by Taylor & Francis.

This is an Open Access article distributed under the terms of the Creative Commons Attribution License (http://creativecommons.org/licenses/by/3.0/), which permits unrestricted use, distribution, and reproduction in any medium, provided the original work is properly cited. The moral rights of the named author(s) have been asserted.

Introduction/Aim: Adolescents undergoing spinal fusion surgery with instrumentation are at great risk to develop kinesiophobia (fear of movement) and consequent morbidities like disability. Identifying who is at risk of developing kinesiophobia after surgery is of paramount importance to improve recovery after such surgery.

Methods: Twenty-three 10–19 year-old patients with Adolescent Idiopathic Scoliosis (AIS) undergoing spinal fusion surgery with instrumentation at the Shriners Hospital were enrolled. Before surgery, kinesiophobia, pain catastrophizing, anxiety trait and pain intensity were assessed with self-reported questionnaires. After surgery, kinesiophobia was measured with the Tampa scale questionnaire on postoperative day 1, 2, 5 and 6 weeks after surgery. Functional disability was assessed 6 weeks after surgery. Correlation and paired t-tests analyses were performed.

Results: Anxiety trait and pain intensity before surgery were negatively correlated with kinesiophobia six weeks after surgery (r = −0.42, p = 0.07 and r = −0.46, p = 0.04 respectively). Pain catastrophizing and helplessness positively correlated with kinesiophobia during the acute postoperative period: day 1, r = 0.43 and r = 0.42; day 2, r = 0.41 and r = 0.45; and day 5, r = 0.35 and r = 0.44. Pain catastrophizing, helplessness and magnification during postoperative days 2 and 5 were associated with kinesiophobia six weeks later (p < 0.05). Six weeks after surgery, 80% of the patients developed kinesiophobia that was associated with functional disability (r = 0.39, p = 0.06) and helplessness(r = 0.44, p = 0.06).

Discussion/Conclusions: Presence of kinesiophobia after surgery was observed in the majority of patients and was associated with pain catastrophizing and functional disability. There is a clinical need to better characterize the observed fear of movement that could interfere with proper recovery after surgery.

## ECHO Ontario: Impact on health care providers

Andrea Furlan^a^, Jane Zhao^a^, Samah Hassan^a^, Jennifer Voth^b^, Jennifer Stinson^c^, Susan Jaglal^d^, Ralph Fabico^a^, Andrew Smith^e^, Paul Taenzer^f^, John Flannery^a^, and Ruth Dubin^g^

^a^ECHO Ontario, MSK Outpatient Department, Toronto Rehabilitation Institute, University Health Network, Toronto, Ontario, Canada; ^b^ICES, Toronto, Ontario, Canada; ^c^Chronic Pain Program, Hospital for Sick Children, Toronto, Ontario, Canada; ^d^Department of Physical Therapy, University of Toronto, Toronto, Ontario, Canada; ^e^Centre for Addiction and Mental Health (CAMH), Toronto, Ontario, Canada; ^f^Departments of Medicine, Oncology, and Psychiatry, University of Calgary, Calgary, Alberta, Canada; ^g^Department of Family Medicine, Queen’s University, Kingston, Ontario, Canada

**CONTACT** Andrea Furlan andrea.furlan@uhn.ca

© 2017 Andrea Furlan, Jane Zhao, Samah Hassan, Jennifer Voth, Jennifer Stinson, Susan Jaglal, Ralph Fabico, Andrew Smith, Paul Taenzer, John Flannery, Ruth Dubin. Published with license by Taylor & Francis.

This is an Open Access article distributed under the terms of the Creative Commons Attribution License (http://creativecommons.org/licenses/by/3.0/), which permits unrestricted use, distribution, and reproduction in any medium, provided the original work is properly cited. The moral rights of the named author(s) have been asserted.

Introduction and Aim: The **E**xtension for **C**ommunity **H**ealthcare **O**utcomes Ontario Chronic Pain and Opioid Stewardship (ECHO for short) uses telehealth technology to bridge specialists in academic centres to health care providers (HCPs) in remote areas. ECHO aims to disseminate knowledge regarding chronic pain (CP) and enhance HCPs’ capacity for opioid use. This study evaluated the impact of ECHO on HCPs’ self-efficacy, attitudes, and satisfaction, and examined whether these outcomes are influenced by profession, presenting a case, or attendance.

Methods: A pre-post online questionnaire was administered to HCPs who attended ECHO sessions. The questionnaire assessed: 1. Self-efficacy in CP management using a 19-item validated questionnaire; 2. HCP attitudes using 7 items from the KnowPain-12 validated instrument; 3. Satisfaction using an 11-item validated questionnaire, administered only Post-ECHO.

Results: From 2014 to 2016, 215 HCPs attended ECHO. 196 (91.2%) completed the pre-ECHO questionnaire. Of the 176 participants who exited ECHO, 89 (50.6%) of them completed post-ECHO questionnaires. A significant increase in self-efficacy mean scores was found between pre- and post-ECHO scores. 3 items demonstrated a significant change in attitudes from pre- to post-ECHO. As for satisfaction, participants ranged from 67.7% to 96.9% in agreement. A significant change in pre and post-ECHO attitudes scores was detected between physicians, nurses, and pharmacists but not other professions. Case presentation and attendance significantly affected satisfaction scores. Professions, case presentation or attendance influenced neither self-efficacy scores nor satisfaction ratings.

Discussion and Conclusion: ECHO demonstrated an effective and feasible strategy to build capacity and increase access to CP management and safe opioid prescribing.

## Is chronic pain associated to cognitive impairments after moderate-to-severe traumatic brain injury?

Caroline Arbour^a^, Héjar El-Khatib^b^, Bérengère Houzé^c^, Gilles J. Lavigne^d^, and Nadia Gosselin^b^

^a^Faculty of Nursing, Université de Montréal, Montréal, Quebec, Canada; ^b^Department of Psychology, Université de Montréal, Montreal, Quebec, Canada; ^c^Research Center, Hôpital du Sacré-Coeur de Montréal, Montréal, Quebec, Canada; ^d^Faculty of Dental Medicine, Université de Montréal, Montréal, Quebec, Canada

**CONTACT** Caroline Arbour caroline.arbour@umontreal.ca

© 2017 Caroline Arbour, Héjar El-Khatib, Bérengère Houzé, Gilles J. Lavigne, and Nadia Gosselin. Published with license by Taylor & Francis.

This is an Open Access article distributed under the terms of the Creative Commons Attribution License (http://creativecommons.org/licenses/by/3.0/), which permits unrestricted use, distribution, and reproduction in any medium, provided the original work is properly cited. The moral rights of the named author(s) have been asserted.

Introduction/Aim: Memory and attention disturbances are common after a traumatic brain injury (TBI), but chronic pain could accentuate cognitive impairments in these patients. This study examined whether chronic pain is associate to neuropsychological performances in moderate-to-severe TBI survivors.

Methods: Clinically meaningful chronic pain (defined as a persistent and/or recurrent pain ≥3/10 experienced over the last 3 months) was determined with the Brief Pain Inventory. The Digit span, the Color Stroop Task, the Trail-Making Test, and the Mesulam cancellation test were used to assess cognitive function. The Beck Anxiety and Depression Inventories, and the Pain Catastrophizing Scale were also used.

Results: In total (N = 39), n = 19 patients *with* and n = 20 patients *without* chronic pain were investigated 10–36 months post-TBI. Both groups were similar in terms of age (32±16 years), gender (65% male), education (14±7 years), and time elapsed since TBI (22±9 months). Participants with chronic pain reported an average pain intensity of 5±3 in the last 24-hour. Despite presenting more extended brain injuries, no difference in cognitive performances was found between participants with and without chronic pain. Participants with chronic pain exhibited significant higher levels of anxiety (13±9 vs. 7±7) and catastrophizing (15±12 vs. 8±7), compared to those without pain. Linear regression (stepwise) showed that increased depression scores were independent predictors of chronic pain intensity after TBI (Beta = 1.190, *p* = 0.009).

Discussion/Conclusions: Unlike our initial hypothesis, our findings suggest that chronic pain is not associate to cognitive deficits after moderate-to-severe TBI. Chronic pain however could enhance the risk of mood disturbances in these individuals.

## Loss of the molecular brake, STEP_61_, connects BDNF-mediated disinhibition to NMDAR potentiation during pathological pain processing within the dorsal horn

Annemarie Dedek^a^, Jian Xu^b^, Chaya M. Kandegedara^a,^^c^, Amy C. Silver^a^, Eve C. Tsai^c,^^d,^^e^, Paul J. Lombroso^b^, and Michael E. Hildebrand^a^

^a^Department of Neuroscience, Carleton University, Ottawa, Ontario, Canada; ^b^The Child Study Centre, Yale University School of Medicine, Yale University, New Haven, Connecticut, USA; ^c^Department of Neuroscience, Ottawa Health Research Institute, Ottawa, Ontario, Canada; ^d^Division of Neurosurgery, Faculty of Medicine, University of Ottawa, Ottawa, Ontario, Canada; ^e^Division of Neurosurgery, The Ottawa Hospital, Ottawa, Ontario, Canada

**CONTACT** Annemarie Dedek annemariededek@cmail.carleton.ca

© 2017 Annemarie Dedek, Jian Xu, Chaya M. Kandegedara, Amy C. Silver, Eve C. Tsai. Paul J. Lombroso, Michael E. Hildebrand. Published with license by Taylor & Francis.

This is an Open Access article distributed under the terms of the Creative Commons Attribution License (http://creativecommons.org/licenses/by/3.0/), which permits unrestricted use, distribution, and reproduction in any medium, provided the original work is properly cited. The moral rights of the named author(s) have been asserted.

Introduction/Aim: The spinal dorsal horn is an essential network for both physiological and pathological pain processing. We have recently shown that in nerve-injured rats, BDNF-mediated disinhibition gates the potentiation of GluN2B-containing NMDA receptors through Fyn kinase activation at lamina I dorsal horn synapses (*Hildebrand et al, Cell Reports, 2016*). We aim to explore whether loss of an associated phosphatase, STEP_61_ (*Xu et al, J Neurochem, 2015*), mediates this pathological coupling in lamina I neurons of both rodents and humans, including following chronic inflammation.

Methods: We paired patch-clamp electrophysiological recordings with pharmacology, behaviour, and biochemical approaches to explore mechanisms for dysregulation of lamina I NMDARs. An *ex vivo* BDNF model of spinal pathology was used in both rodent and human tissue, and an *in vivo* injection of Freund’s adjuvant into the rodent hindpaw was used to model chronic inflammatory pain.

Results: In all models, we observed a decrease in STEP_61_ and an increase in pGluN2B and pFyn at lamina I synapses. Downregulation of STEP_61_ was both necessary and sufficient to prime subsequent phosphorylation and potentiation of synaptic NMDARs by BDNF. Importantly, we also showed that inflammatory hypersensitivity was reversed by attenuating disinhibition using IP injected acetazolamide, and paired this with biochemical analysis to investigate lamina I synaptic signalling.

Discussion/Conclusions: Our results suggest that STEP_61_ is the molecular brake that is lost to drive the potentiation of excitatory NMDAR responses following BDNF-mediated disinhibition at lamina I synapses of both rodents and humans. Thus, STEP_61_ modulation may be a useful pharmaceutical target for treating pathological pain conditions.

## Management of opioid-induced hypogonadism among patients with chronic non-cancer pain (CNCP): A systematic review

Mahmood AminiLari^a^, Priya Mangoo^b^, Samantha Craigie^c^, Rachel Couban^c^, Li Wang^c^, and Jason W. Busse^d^

^a^Health Research Methods, Evidence, and Impact, McMaster University, Hamilton, Ontario, Canada; ^b^Endocrinology, University of British Columbia, Victoria, British Columbia, Canada; ^c^Michael G. DeGroote Institute for Pain Research and Care, McMaster University, Hamilton, Ontario, Canada; ^d^Anesthesia, McMaster University, Hamilton, Ontario, Canada

**CONTACT** Mahmood AminiLari aminila@mcmaster.ca

© 2017 Mahmood AminiLari, Priya Mangoo, Samantha Craigie, Rachel Couban, Li Wang, and Jason W. Busse. Published with license by Taylor & Francis.

This is an Open Access article distributed under the terms of the Creative Commons Attribution License (http://creativecommons.org/licenses/by/3.0/), which permits unrestricted use, distribution, and reproduction in any medium, provided the original work is properly cited. The moral rights of the named author(s) have been asserted.

Introduction/Aim: Use of opioids for chronic noncancer pain may affect patients’ endocrine function, including opioid induced hypogonadism. We systematically reviewed the literature for evidence regarding management of opioid-induced hypogonadism.

Methods: We searched for studies, in any language, with tailored searches of MEDLINE, CINAHL, AMED, CENTRAL, CINAHL, DARE, EMBASE, and PsycINFO, through to August 2016. Our eligibility criteria were a) randomized controlled trials or controlled/uncontrolled observational studies with ≥ 20 patients, b) enrollment of chronic noncancer pain patients with opioid-induced hypogonadism, c) assessment of any intervention to treat or manage opioid-induced hypogonadism. Pairs of reviewers worked independently to determine eligibility status of all identified citations through screening titles and abstracts, and then full text of all potentially eligible studies. Reviewers abstracted information, independently and in duplicate, using standardized forms. Only effects on patient-important outcomes were collected.

Results: Of 634 abstracts reviewed, 5 studies including one randomized controlled trial (65 patients) and four observational studies (129 patients) were eligible. Very low quality evidence found that testosterone replacement therapy was associated with improvements in pain, and depressive symptoms. Very low quality evidence found no effect of testosterone replacement therapy on sexual function or physical functioning.

Discussion/Conclusions: There is limited evidence, of very low quality, regarding management of opioid-induced hypogonadism. The available evidence suggests that testosterone replacement therapy be helpful; however, large, rigorously conducted, randomized controlled trials, are needed to establish the role of testosterone replacement therapy in the management of opioid-induced hypogonadism.

## Evaluating as if, an interactive body-sensing game to foster empathy towards chronic pain patients

Xin Tong^a^, Weina Jin^a^, Servet Ulas^a^, Diane Gromala^a^, Chris Shaw^a^, and Owen Williamson^b^

^a^School of Interactive Arts and Technology, Simon Fraser University, Surrey, BC, Canada; ^b^School of Public Health and Preventive Medicine, Monash University, Surrey Memorial Pain Clinic, Surrey, BC, Canada

**CONTACT** Xin Tong tongxint@sfu.ca

© 2017 Xin Tong, Weina Jin, Servet Ulas, Diane Gromala, Chris Shaw, and Owen Williamson. Published with license by Taylor & Francis.

This is an Open Access article distributed under the terms of the Creative Commons Attribution License (http://creativecommons.org/licenses/by/3.0/), which permits unrestricted use, distribution, and reproduction in any medium, provided the original work is properly cited. The moral rights of the named author(s) have been asserted.

Introduction/Aim: It can be difficult for healthcare understan/understand the suffering of Chronic Pain (CP) patients, let alone to empathize with them, leading to frustration, anger, stigmatization and social isolation. AS IF is an interactive “serious game”, in which players “embody” an avatar that responds to a player’s actions as if it were their own body. The aim of this study was to determine if playing AS IF improves empathy and Willingness to Help CP (WHCP) patients.

Methods: A mixed-methods study was conducted in a convenience sample of people over 18 years old. Outcomes were measured using the revised Compassion for Others Scale (COS) and the self-reported WHCP patients on a visual analog scale. A semi-structured interview was conducted.

Results: Fifteen people participated in this study aged 20 to 34, 4 female. There was a significant increase in post-intervention WHCP score compared to the pre-intervention score (p < 0.05), with a median effect size. There was no significant increase in the COS (p = 0.081).

Discussion/Conclusions: The results from the study indicate that by inhibiting a virtual body that had some of the limitations CP patients might have and engaging in tasks in the simulation game, players achieved a significant increase in WHCP. The game failed to significantly increase empathy for CP patients, possibly because of flaws in game design, the age demographic, the short duration and insufficient repeated exposure. Further modifications may provide a useful interactive storytelling tool to help foster awareness of CP.

## Factors associated with disability benefits claim duration among Canadian workers: A retrospective cohort study

Sohail M. Mulla^a^, Sun Makosso-Kallyth^b^, Nathalie St-Hilaire^c^, Katrena Munsch^c^, Peter B. Gove^c^, Diane Heels-Ansdell^a^, Gordon H. Guyatt^a^, and Jason W. Busse^b^

^a^Health Research Methods, Evidence, and Impact, McMaster University, Hamilton, Ontario, Canada; ^b^Anesthesia, McMaster University, Hamilton, Ontario, Canada; ^c^Life & Disability, SSQ Life Insurance Company, Toronto, Ontario, Canada

**CONTACT** Sohail M. Mulla mullasm@mcmaster.ca

© 2017 Sohail M. Mulla, Sun Makosso-Kallyth, Nathalie St-Hilaire, Katrena Munsch, Peter B. Gove, Diane Heels-Ansdell, Gordon H. Guyatt, and Jason W. Busse. Published with license by Taylor & Francis.

This is an Open Access article distributed under the terms of the Creative Commons Attribution License (http://creativecommons.org/licenses/by/3.0/), which permits unrestricted use, distribution, and reproduction in any medium, provided the original work is properly cited. The moral rights of the named author(s) have been asserted.

Introduction/Aim: Disability insurance protects workers from total loss of income in case of a disabling injury or illness by providing wage-replacement benefits. To better inform early identification of claims at risk of prolonged recovery, we explored predictors of disability benefits claim duration.

Methods: Using administrative data from SSQ Financial, a private Canadian disability insurer, we evaluated the association between nine variables and short-term disability and long-term disability benefits duration using Cox proportional hazards regression analyses.

Results: We analyzed 70,776 short-term disability and 22,205 long-term disability claims. For both short-term disability and long-term disability claims, and across all disorders, older age, female gender, heavy job demands, presence of comorbidity, attending an independent medical evaluation, receipt of rehabilitation therapy, and longer time to claim approval were associated with longer claim duration. Higher pre-disability salary was associated with longer short-term disability claim duration. Quebec residency was associated with longer short-term disability claim duration among workers with psychological disorders, but shorter short-term disability claim duration among those with musculoskeletal diseases and other illnesses. For long-term disability claims, however, residing in Quebec was associated with shorter claim duration, although the magnitude of the association differed across clinical conditions.

Discussion/Conclusions: The factors we found to be associated with short-term and long-term disability claim duration may be helpful to identify claims at risk of prolonged recovery. Our study has limitations, however, and well-designed prospective studies are needed to confirm our findings and identify other promising predictors.

## Sensitivity to physical activity: A novel indicator of low levels of physical activity in daily life

Arthur Woznowski-Vu^a^, Jordan Miller^b^, Timothy Wideman^a^

^a^School of Physical and Occupational Therapy, McGill University, Montreal, Quebec, Canada; ^b^School of Rehabilitation Therapy, Queen’s University, Kingston, Ontario, Canada

**CONTACT** Arthur Woznowski-Vu arthur.woznowskivu@gmail.com

© 2017 Arthur Woznowski-Vu, Jordan Miller, Timothy Wideman. Published with license by Taylor & Francis.

This is an Open Access article distributed under the terms of the Creative Commons Attribution License (http://creativecommons.org/licenses/by/3.0/), which permits unrestricted use, distribution, and reproduction in any medium, provided the original work is properly cited. The moral rights of the named author(s) have been asserted.

Introduction/Aim: Chronic pain is often disabling and can be associated with reduced participation in physical activity. Sensitivity to physical activity (SPA) is emerging as an important predictor of poor outcomes in pain-related conditions, but it is currently not clear whether SPA is associated with reduced participation in physical activity. The aim of this study is to identify if high levels of SPA are associated with low levels of self-reported physical activity among adults with chronic musculoskeletal pain (>3 months) at 9-day follow-up.

Methods: Preliminary analysis was conducted on 47 participants from a longitudinal study on a cohort of people with chronic musculoskeletal pain (>3months). SPA was measured by calculating the mean difference in self-reported numerical pain rating scale scores (0-100) and the mean difference in pressure pain thresholds (kPa) from before to after a standardized walking (6-minute walk test) and lifting (18 weighted canisters) tasks. Physical activity levels were obtained via the International Physical Activity Questionnaire (IPAQ, long version, telephone format) nine days after the in-person testing session. For data analysis, a median split was used to divide participants into two groups: high or low SPA.

Results: Participants with high SPA were found to have lower total daily physical activity than participants with low SPA. This difference was statistically significant.

Discussion/Conclusions: This study suggests that a patient’s level of sensitivity physical activity is an important indicator of their level of physical activity in daily life.

## Preoperative pregabalin or gabapentin for postoperative acute and chronic pain among patients undergoing breast cancer surgery: A systematic review

Ajit S. Rai^a^, James S. Khan^b^, Jasneet Dhaliwal^c^, Jason W. Busse^d^, Stephen Choi^b^, P.J. Devereaux^e^, and Hance Clarke^b^

^a^Medicine, Wayne State University, Detroit, Michigan, USA; ^b^Anesthesia, University of Toronto, Toronto, Ontario, Canada; ^c^Faculty of Health Sciences, McMaster University, Hamilton, Ontario, Canada; ^d^Anesthesia, McMaster University, Hamilton, Ontario, Canada; ^e^Medicine, McMaster University, Hamilton, Ontario, Canada

**CONTACT** Ajit S. Rai ajitrai99@gmail.com

© 2017 Ajit S. Rai, James S. Khan, Jasneet Dhaliwal, Jason W. Busse, Stephen Choi, P.J. Devereaux, Hance Clarke. Published with license by Taylor & Francis.

This is an Open Access article distributed under the terms of the Creative Commons Attribution License (http://creativecommons.org/licenses/by/3.0/), which permits unrestricted use, distribution, and reproduction in any medium, provided the original work is properly cited. The moral rights of the named author(s) have been asserted.

Introduction/Aim: Breast cancer surgery is associated with acute and chronic post-surgical pain. We conducted a systematic review to evaluate the effect of gabapentin and pregabalin on post-surgical pain among patients undergoing breast cancer surgery.

Methods: We searched MEDLINE, EMBASE, CENTRAL, Web of Science, and ProQuest from inception to November 2015. Studies enrolling adult patients undergoing breast cancer surgery randomly assigned to receive preoperative gabapentin or pregabalin versus placebo or active control, and reported effects on acute (≤24 hours) or chronic (≥2 months) pain, were included. We conducted meta-analyses when possible, and rated quality of evidence (QoE) using the GRADE approach.

Results: Twelve studies were eligible for review. Eight evaluated gabapentin (n=516) and four pregabalin (n = 209). Gabapentin reduced pain scores in the recovery room (mean difference [MD] −1.68 on a 0–10 numeric rating scale, 95% CI −2.59 to −0.77; minimally important difference is 1-point; relative risk [RR] for mild pain (<4/10) 1.71, 95% CI 1.33 to 2.02; moderate QoE) and 24-hours postoperatively (MD −0.52, 95% CI −1.02 to −0.01; RR for mild pain 1.07, 95% CI 1.00 to 1.13; very low QoE). Pregabalin reduced pain in the recovery room, but not at 24-hours (MD −0.38, 95%, CI −0.96 to 0.21; moderate QoE). Neither drug reduced the rate of chronic post-surgical pain.

Discussion/Conclusions: Current evidence suggests that gabapentin and pregabalin reduces pain in the operating room, and that gabapentin, but not pregabalin, reduces pain at 24 hours after breast cancer surgery. Neither gabapentin or pregabalin affect the development of chronic post-surgical pain.

## The efficacy of using cardboard mobile virtual reality for chronic pain patients’ pain distraction in clinical settings

Ashfaq Amin^a^, Xin Tong^a^, Diane Gromala^a^, and Pam Squire^b^

^a^School of Interactive Arts and Technology,Simon Fraser University, Surrey, BC, Canada; ^b^School of Medicine, University of British Columbia, Vancouver, BC, Canada

**CONTACT** Ashfaq Amin aamin@sfu.ca

© Ashfaq Amin, Xin Tong, Diane Gromala, and Pam Squire. Published with license by Taylor & Francis.

This is an Open Access article distributed under the terms of the Creative Commons Attribution License (http://creativecommons.org/licenses/by/4.0/), which permits unrestricted use, distribution, and reproduction in any medium, provided the original work is properly cited.

Introduction/Aim: Immersive Virtual Reality (VR) has been shown to work as a non-pharmacological analgesic by enabling cognitive distraction in acute pain patients.However, little research literature exists on the effectiveness of cardboard VR for Chronic Pain (CP) patients. Therefore, this poster aims at comparing the viability of Cardboard VR and Oculus Rift (OR) desktop VR for CP management.

Methods: Thirty adult participants (17 Males, aged 23–68) were recruited in a pain clinic. The research study was a within-subject study comparing Cardboard VR with OR. The participants experienced both conditions, and filled out the same Pre-VR and Post-VR questionnaires before and after each VR condition. The questionnaires asked them to rate their present pain intensity and retroactive pain intensity on a scale of 1–100.

Results: The pairwise comparison using Bonferroni post hoc analysis showed, the difference between Pre-VR and Oculus Rift was significant, p = 0.017. However, the difference between Pre-VR and Cardboard was not significant, p = 0.216.

Discussion/Conclusions: The study results showed that Cardboard VR, coupled with a smartphone, is capable of reducing the patients’ perceived pain intensity significantly compared to the control (pre-VR) condition. However, despite the early findings from the previous studies, OR was found to be significantly more effective in pain patients than both the Cardboard and the control condition. The results of this study encourage future research inquiries of Mobile VR in management of chronic pain. Mobile VR, because of its affordability and ease of use, shows the potential to become an effective tool for pain management for the patients.

## Is facial coding reliability dependent on observer point of view? Comparing pain expressions from various angles of observation

M. Erin Browne^a^, Thomas Hadjistavropoulos^a^, Kenneth M. Prkachin^b^, Babak Taati^c^, and Ahmed Ashraf^c^

^a^Psychology, University of Regina, Regina, Saskatchewan, Canada; ^b^Psychology, University of Northern British Columbia, Prince George, BC, Canada; ^c^Computer Science, Toronto Rehabilitation Institute, Toronto, Ontario, Canada

**CONTACT** M. Erin Brownebrowne3m@uregina.ca

© 2017 M. Erin Browne, Thomas Hadjistavropoulos, Kenneth M. Prkachin, Babak Taati, and Ahmed Ashraf. Published with license by Taylor & Francis.

This is an Open Access article distributed under the terms of the Creative Commons Attribution License (http://creativecommons.org/licenses/by/4.0/), which permits unrestricted use, distribution, and reproduction in any medium, provided the original work is properly cited.

Introduction/Aim: The facial expression of pain is a reliable non-verbal signal, but may be interpreted differently from different viewing angles. Fine-grained observational approaches (e.g., Facial Action Coding System; FACS), used in the evaluation of pain responses, are based on a full view of the face. The contribution of certain viewing angles to facial coding accuracy is unknown. We compared inter-rater reliability from side view and full view coding of pain responses.

Methods: Videos of older adults (with and without dementia) undergoing a series of physiotherapy movements were coded for facial pain responses based on FACS (incorporating action intensity), and gross pain behaviors using a clinical observation method. Separate coding was done for full (incorporating left, right, and front views), and left side views of the face. A second coder independently coded videos for reliability calculation.

Results: Reliability was calculated for each coding system. Percentage agreement for side coding is 71.18% (κ = 0.69, *p* < .001; ρ = 0.73, *p* < .001). Percentage agreement for full coding is 77.00% (κ = 0.76, *p* < .001; ρ = 0.99, *p* < .001). Comparing side to full view coding, coders achieved 44.72% agreement (κ = 0.48, *p* < .001; ρ = 0.41, *p* < .001).

Discussion/Conclusions: Similar levels of inter-rater agreement can be achieved in coding the full view and the side view of the face during pain. Side and full coding do not provide the same assessment of facial expression, as evidenced by weaker inter-rater reliability between the two. Potential implications for pain sufferers will be discussed.

## What are registered nurses’ experiences in delivering pain care to pediatric patients in rural Northern Ontario?

Carolyn Truskoski 0000-0003-3925-9418^a^, Paula Forgeron 0000-0002-4686-9698^a^, Denise Harrison 0000-0001-7549-7742^a^, and Nancy L. Young 0000-0002-1739-3299^b^

^a^Faculty of Health Sciences, School of Nursing, University of Ottawa, Ottawa, Ontario, Canada; ^b^Faculty of Health, School of Rural and Northern Health, Laurentian University, Sudbury, Ontario, Canada

**CONTACT** Carolyn Truskoski ctrus063@uottawa.ca

© 2017 Carolyn Truskoski, Paula Forgeron, Denise Harrison, and Nancy L. Young. Published with license by Taylor & Francis.

This is an Open Access article distributed under the terms of the Creative Commons Attribution License (http://creativecommons.org/licenses/by/4.0/), which permits unrestricted use, distribution, and reproduction in any medium, provided the original work is properly cited.

Introduction/Aim: To explore registered nurses (RNs) experiences in providing pain care to children in the rural hospital setting and understand the challenges and facilitators.

Methods: An exploratory descriptive qualitative study using semi-structured interviews by Skype or telephone with RNs who work at one of the nine eligible sites in rural Northern Ontario. Eligible sites were those in Northern Ontario with dedicated inpatient pediatric beds. Purposeful and snowball sampling was used. Recruitment was done via a mail out strategy, social media campaign, and community advertisement. Data were analyzed using inductive content analysis. A coding sheet was developed and categories were mapped, and collapsed into main categories.

Results: There were 5 main categories identified. RNs needed to practice as *generalists*, which threaded across all categories. *Resource challenges* included a lack of specialist expertise and educational opportunities. Distance education strategies did not consider the context challenges or preference of learning of the nurses. Pediatric pain was *not formally identified as a priority* within their practice or institution. Most participants stated there were *no explicit standards* for pain. However, topical anaesthetics for skin breaking procedures were commonly used but sucrose for infants was not, illustrating that the adoption of evidenced informed pain strategies may be random. *Moving forward* adoption of preprinted orders was suggested as a possible facilitator for use of evidenced-based pain management.

Discussion/Conclusions: There is opportunity to improve pediatric pain management in rural Northern Ontario. However, strategies above and beyond distance education are needed that consider the challenges of the rural context.

## Early diffusivity alterations in the trigeminal nerve as prognosticators of clinical outcome after gamma knife radiosurgery

Sarasa Tohyama, Peter Shih-Ping Hung, and Mojgan Hodaie

Institute of Medical Science, University of Toronto, Toronto, Ontario, Canada

**CONTACT** Sarasa Tohyama sarasa.tohyama@mail.utoronto.ca

© 2017 Sarasa Tohyama, Peter Shih-Ping Hung, and Mojgan Hodaie. Published with license by Taylor & Francis.

This is an Open Access article distributed under the terms of the Creative Commons Attribution License (http://creativecommons.org/licenses/by/3.0/), which permits unrestricted use, distribution, and reproduction in any medium, provided the original work is properly cited. The moral rights of the named author(s) have been asserted.

Introduction/Aim: Radiosurgery is an important treatment modality for trigeminal neuralgia (TN), a severe neuropathic facial pain disorder. Despite extensive clinical use and high efficacy of Gamma Knife radiosurgery (GKRS) for the treatment of TN, a viable prognostic model has not been established. Using diffusion tensor imaging (DTI), we aimed to determine whether early trigeminal nerve microstructural alterations as a consequence of radiosurgery would predict long-term treatment response.

Methods: 3T-MR data were acquired from 32 TN patients (20F, age 68.8±13.5), 6 months post-GKRS. Tissue microstructure measures of fractional anisotropy (FA), axial, radial, and mean diffusivities (AD, RD, and MD, respectively) were extracted from the radiosurgical target area of the affected trigeminal nerve. The contralateral, asymptomatic nerve served as the control. Early, 6-month trigeminal nerve microstructure was compared with long-term clinical results. Patients were classified as responders if they achieved at least 75% reduction in preoperative pain for 12 months or longer following treatment.

Results: Based on clinical follow-up data, we identified 17 long-term responders and 15 non-responders. Radiosurgical target FA value at 6 months was predictive of long-term clinical results, demonstrating significant lower FA in responders versus non-responders. There was no significant change in FA of the asymptomatic nerve between the two groups.

Discussion/Conclusions: Early trigeminal nerve microstructural alterations as a result of radiosurgery successfully prognosticate long-term treatment response. Specifically, the lower FA of responders, which is indicative of disrupted nerve organization, prognosticates better long-term pain relief. DTI serves as a promising tool to assess the effects and prognosis of radiosurgery on the trigeminal nerve.

## Predictors of suicidal ideation in an ohip-funded interdisciplinary chronic pain management program

Laura Katz^a^, Eleni G.Hapidou^b^

^a^Michael G. DeGroote Pain Clinic, Hamilton Health Sciences, Hamilton, Ontario, Canada; ^b^Psychiatry and Behavioural Neurosciences & Michael G. DeGroote Pain Clinic, McMaster University and Hamilton Health Sciences, Hamilton, Ontario, Canada

**CONTACT** Laura Katz katzl@hhsc.ca

© 2017 Laura Katz and Eleni G. Hapidou. Published with license by Taylor & Francis.

This is an Open Access article distributed under the terms of the Creative Commons Attribution License (http://creativecommons.org/licenses/by/3.0/), which permits unrestricted use, distribution, and reproduction in any medium, provided the original work is properly cited. The moral rights of the named author(s) have been asserted.

Introduction/Aim: Individuals suffering from chronic pain are at an increased risk for suicidality. The aim of this study was to evaluate predictors of suicidal ideation in a sample of OHIP patients in an interdisciplinary chronic pain management program.

Methods: Patients referred to psychological services in an interdisciplinary chronic pain program completed self-report questionnaires as part of the assessment process including demographic information and measures for pain, bothersome symptoms (PHQ-15), depression (PHQ-9) including the item for suicidal ideation, anxiety (GAD-7), disability (PDI), pain-catastrophizing (PCS) and coping (B-CPCI). Data were analyzed using descriptive statistics, and linear regressions were employed to evaluate the predictors of suicidal ideation.

Results: A total of 33 patients completed the questionnaires, and 42% (n=14) patients reported suicidal ideation in the past 2 weeks. Suicidal ideation was best predicted by catastrophizing (β=0.50, *p*=0.05) and disability (β=0.45, *p*=0.02), over and above average pain, somatic symptoms, anxiety and depression (*F*=4.50, *p*<0.01). In terms of coping, suicidal ideation was best predicted by guarding (β=0.71, *p*<0.01), and resting (β=0.50, *p*=0.02), over and above asking for assistance, relaxing, task persistence, exercising/stretching, seeking social support and positive self-talk (*F*=2.93, *p*=0.02).

Discussion/Conclusions: Suicidal ideation was best predicted by passive coping (guarding and resting) deactivation (increased disability) and maladaptive pain appraisals (catastrophizing). Sedentary behaviours and anxiety-related to pain appear to be predictive of suicidal ideation, and as such treatment of patients referred to psychology within the OHIP-funded interdisciplinary chronic pain program should focus on activation and active-coping skills as well as cognitive therapy to improve pain catastrophizing.

## Empirical Validation of the Limitations in Daily Activities Scale (LIDAS) in Chronic Pain

Keith G. Wilson^a^, Dyana Castillo^b^, John Kowal^a^, An Gie Yong^b^, and Lachlan A. McWilliams^c^

^a^Department of Psychology, The Ottawa Hospital Rehabilitation Centre, Ottawa, ON, Canada; ^b^Department of Psychology, The University of Ottawa, Ottawa, ON, Canada; ^c^Department of Psychology, The University of Saskatchewan, Saskatoon, SK, Canada

**CONTACT** Dyana Castillo dcast094@uottawa.ca

© Dyana Castillo and Keith G. Wilson. Published with license by Taylor & Francis.

This is an Open Access article distributed under the terms of the Creative Commons Attribution License (http://creativecommons.org/licenses/by/3.0/), which permits unrestricted use, distribution, and reproduction in any medium, provided the original work is properly cited. The moral rights of the named author(s) have been asserted.

Introduction/Aim: Functional ability represents a core dimension of outcome assessment in chronic pain, both for documenting patient progress and for evaluating program effectiveness. In 1995, the International Association for the Study of Pain (IASP) circulated a self-report measure of functional ability. Although the measure has sometimes been used in research studies, it has never been subjected to a thorough investigation of its psychometric properties.

Methods: In this study, 950 patients with chronic pain completed the 16-item Limitations in Daily Activities Scale (LIDAS) at two pre-treatment and one post-treatment assessment. Individual item analysis was conducted and the results favoured a 13-item scale.

Results: Exploratory Factor Analyses suggest that the LIDAS has a 3-factor structure consisting of Daily Tasks, Global Participation, and Personal Care. Reliability analyses for the total scale (*α* = 0.90) as well as for each factor (*α*s = 0.82 to 0.84) indicated excellent internal consistency, good-excellent test-retest reliability across factors (*r*s = 0.66 to 0.88), and sensitivity to change with treatment. LIDAS scores correlated with measures of pain intensity, pain cognitions, and depression, as well as with other self-report and clinician-administered measures of functional performance. A minimal clinically important difference of 5 points was established for assessing meaningful individual improvement after treatment.

Discussion/Conclusions: In conclusion, the LIDAS is a reliable, valid, and clinically relevant option for assessing functional ability in patients with chronic pain.

## Virtual reality for procedural pain and anxiety in young children with burn injuries: A pilot study

Christelle Khadra^a^, Sylvie Charette^b^, Ariane Ballard^a^, Viviane Tremblay^c^, David Paquin^d^, Jean-Simon Fortin^b^, Johanne Déry^a^, Edith Villeneuve^a^, Isabelle Perreault^a^, Hunter Hoffman^e^, and Sylvie Le May^a^

^a^University of Montreal; ^b^CHU Sainte-Justine; ^c^Centre Hospitalier de l’Université de Montréal; ^d^Université du Québec en Abitibi-Témiscamingue; ^e^University of Washington

**CONTACT** Sylvie Le May sylvie.lemay@umontreal.ca

© 2017 Christelle Khadra, Sylvie Charette, Ariane Ballard, Viviane Tremblay, David Paquin, Jean-Simon Fortin, Johanne Déry, Edith Villeneuve, Isabelle Perreault, Hunter Hoffman, and Sylvie Le May. Published with license by Taylor & Francis.

This is an Open Access article distributed under the terms of the Creative Commons Attribution License (http://creativecommons.org/licenses/by/3.0/), which permits unrestricted use, distribution, and reproduction in any medium, provided the original work is properly cited. The moral rights of the named author(s) have been asserted.

Aims: Hydrotherapy is a painful procedure associated with treatment of burn injuries. Very few studies have used virtual reality (VR) for procedural pain and anxiety in young children with burn injuries. The aim of this study was to assess the feasibility and acceptability of a VR prototype for procedural pain and anxiety in children with burn injuries.

Methods: This prospective pilot study recruited children from 3 months to 10 y.o. with burn injuries requiring hydrotherapy sessions for wound burn care. Pain was assessed using the FLACC (min 0, max10) and anxiety using the PBCL (Pain Behavioral Check List) (min 8, max 40). Satisfaction of healthcare professionals was also documented using a pretested questionnaire (min 8, max 32).

Results: Fifteen participants were included in the analyses. Mean age was 2.2 ± 2.1 years, and the mean TBSA 5 % (±4%). Pain did not significantly vary before, during and after the procedure with mean FLACC scores respectively at: 2.1 (±2.7), 2.9 (±3), 2.6 (±3). Mean PBCL score was 11.4 (±5.2) during the procedure and the mean score for satisfaction of healthcare professionals was 26.3 (±4.2). VR prototype did not interfere with the procedure and was considered very useful by most healthcare professionals for reducing children’s pain and anxiety.

Conclusion: The VR prototype is a feasible and acceptable method of distraction for procedural pain and anxiety in young children with burn injuries. A larger trial with a control group would be required to assess its efficacy.

## Cytotoxic T cells induce pain hypersensitivity in female but not male mice after nerve injury in an interferon-γ-receptor-dependent manner

B. Ham^a,^^b^, S. Rosen^a,^^b^, N. Boachie ^a^, J. S. Austin^a^, and J. S Mogil^a,^^b^

^a^Psychology, McGill University, Montreal, QC, Canada; ^b^The Alan Edwards Centre for Research on Pain, McGill University, Montreal, QC, Canada

**CONTACT** J. S. Mogil jeffrey.mogil@mcgill.ca

© 2017 B. Ham, S. Rosen, N. Boachie, J. S. Austin, and J. S. Mogil. Published with license by Taylor & Francis.

This is an Open Access article distributed under the terms of the Creative Commons Attribution License (http://creativecommons.org/licenses/by/3.0/), which permits unrestricted use, distribution, and reproduction in any medium, provided the original work is properly cited. The moral rights of the named author(s) have been asserted.

**Aim of investigation**: Sex differences in pain processing mechanisms are increasingly recognized. In our previous study, we observed that female mice, unlike males, do not require microglia to produce pain hypersensitivity after neuropathic or inflammatory injury. Using T-cell deficient mice, we found that female mutant mice “switch” to the “male” microglial system. Important open question included whether T cells are important for female pain.

**Methods**: Mechanical allodynia was assessed after spared nerve injury (SNI) and post complete Freund’s adjuvant (CFA) injection using von Frey fibers. After allodynia was confirmed, CD8, CD4 and CD3 Ab or minocycline were injected, and withdrawal thresholds measured. mRNA was from spleen immune cells were analyzed for Granzyme B, TNF-α and IL-4 by qPCR. Also, immune cells extracted from lumbar spinal cord were immunostained with CD45, CD11b, CD4, CD8, and analyzed using flow cytometry.

**Results**: In contrast to male mice, female mice showed: 1) inhibition of CD8^+^ T cells and CD3^+^ T cells reversed allodynia in female mice, 2) CD8+T cells from female spleen showed increased IFNγ production by 4-fold after SNI, and 3) spinal CD8^+^ T cell level did not decrease after SNI. Interestingly, IFNγRKO female mice showed the same pain behavioural response as wild-type males wherein minocycline reversed allodynia. Consistent with this, IFNγR KO female, like wild-type male, mice did show downregulation of CD8^+^ T cells.

**Conclusion**: Our current data further suggest that female mice, unlike males, are using CD8^+^ T cells to induce neuropathic pain, and that this mechanism appears to be dependent on IFNγRs.

## Toll-like receptor 4 (TLR4) involvement in nociceptive processes within rat medullary dorsal horn in dental inflammatory pain model

Helena Fetter Filippini^a,^^b^, Graziella Molska^a^, Limor Avivi-Arber^a^, Yamini Yarudchelvan^a^, Siew-Ging Gong^a^, Maria Martha Campos^b^, and Barry John Sessle^a^

^a^Faculty of Dentistry, University of Toronto, Toronto, ON, Canada; ^b^Faculty of Dentistry, Pontifícia Universidade Católica do Rio Grande do Sul, Porto Alegre, RS, Brazil

**CONTACT** Helena Fetter Filippini helenafilippini@gmail.com

© 2017 Helena Fetter Filippini, Graziella Molska, Limor Avivi-Arber, Yamini Yarudchelvan, Siew-Ging Gong, Maria Martha Campos, and Barry John Sessle. Published with license by Taylor & Francis.

This is an Open Access article distributed under the terms of the Creative Commons Attribution License (http://creativecommons.org/licenses/by/4.0/), which permits unrestricted use, distribution, and reproduction in any medium, provided the original work is properly cited.

Introduction/Aim: Mustard oil (MO) application to the rat tooth pulp induces trigeminal central sensitization in the medullary dorsal horn (MDH) and sensorimotor nociceptive responses in jaw muscles reflected in increased electromyographic (EMG) activities. Central sensitization has been shown to depend on MDH microglia activation, and TLR4 is involved in microglial activation in pain states. However, the role of TLR4 in CNS mechanisms of dental pain is unclear. Therefore, the aims of this study were to test if TLR-4 expression occurs in MDH, and if bilateral EMG activities in the anterior digastric (AD) muscles evoked by MO application (0.2 μL/95%) to the pulp are dependent on TLR4 processes in MDH

Methods: The first maxillary molar pulp was exposed in adult male Sprague-Dawley rats, and TLR4 antagonist (LPS-RS, 25 μg/10 μl) or isotonic saline (as vehicle control; 8-13 rats/group) was applied to MDH 10 min before pulpal MO application. AD EMG activities were recorded from 15 min prior to LPS-RS or vehicle application, until 15 min after MO application. The ipsi- and contralateral MDH regions were removed after euthanasia for western blot analysis of TLR4 expression in MDH.

Results: TLR4 expression was apparent in MDH, and MO application increased EMG activities in the AD muscles that were significantly reduced following MDH administration of LPS-RS (2-way ANOVA, post-hoc Bonferroni, p=0.0004).

Discussion/Conclusions: TLR4 activation in the MDH may be a mechanism mediating dental inflammatory pain.

## Factors influencing orthopedic nurses’ pain management: A focused ethnography

Kayla Denness^a^, Eloise Carr^b^, Cydnee Seneviratne^b^, and J. M. Rae^a^

^a^Acute Pain Service, Alberta Health Services, Calgary, Alberta, Canada; ^b^Faculty of Nursing, University of Calgary, Calgary, Alberta, Canada

**CONTACT** Kayla Denness kayla.denness@ahs.ca

© 2017 Kayla Denness, Eloise Carr, Cydnee Seneviratne, and Janice M. Rae. Published with license by Taylor & Francis.

This is an Open Access article distributed under the terms of the Creative Commons Attribution License (http://creativecommons.org/licenses/by/4.0/), which permits unrestricted use, distribution, and reproduction in any medium, provided the original work is properly cited.

Aim: Fast-track surgery programs reduce length of stay by identifying and addressing factors that cause patients to remain in hospital, including pain (Kehlet, 2013). The assessment and management of acute pain is an important component of quality care for patients after total knee arthroplasty. The aim of this focused ethnography was to explore the factors influencing orthopedic surgery nurses’ decisions to administer prn opioid analgesia for acute postoperative pain.

Methods: Semi-structured interviews began with a vignette of a patient who received a nerve block for analgesia following total knee arthroplasty, and proceeded to examine factors that influence participants’ pain management. Interviews were transcribed and analyzed using thematic analysis and constant comparison.

Results: The ten nurses who participated described a complex clinical environment where the interplay of several factors informed their decision to administer prn opioid analgesia. The unit’s culture and physical space influenced nurses’ assessment of pain and their decision whether to treat the pain with prn opioids. Each nurse’s self-concept affected pain management decisions because of the perceived importance of pain control and perceived duty to provide analgesics. The subjectivity of pain added another layer of complexity as nurses responded to the patient’s expression of pain from within the milieu of the unit culture and their unique self-concept.

Conclusions: Understanding the complexity of factors that influence nurses’ postoperative pain management provides clinical nurses and nursing leaders with directions for future education and research, guided by the goal of continued improvement in pain management in the setting of fast-track surgeries.

Kehlet, H. (2013). **Fast-track hip and knee arthroplasty**. *The Lancet*, *381*(9878), 1600–1602. doi:10.1016/S0140-6736(13)61003-X

## Myofascial pain syndrome and fibromyalgia: A scoping review of current literature

Sheryl Bourgaize, Genevieve Newton, and John Srbely

Human Health and Nutritional Sciences, University of Guelph, Guelph, Ontario, Canada

**CONTACT** Sheryl Bourgaize sbourgai@uoguelph.ca

© 2017 Sheryl Bourgaize, Genevieve Newton, and John Srbely. Published with license by Taylor & Francis.

This is an Open Access article distributed under the terms of the Creative Commons Attribution License (http://creativecommons.org/licenses/by/4.0/), which permits unrestricted use, distribution, and reproduction in any medium, provided the original work is properly cited.

Introduction/Aim: Fibromyalgia (FM) and Myofascial Pain Syndrome (MPS) are common forms of chronic musculoskeletal pain disorders. Management of these conditions relies on accurate diagnosis. The primary distinction between FM and MPS relies on identification of Tender Points versus Trigger Points (TrPs), respectively. Given the poor inter-rater reliability of identification of TrPs (Myburgh et al. 2008), a novel set of criteria to increase the sensitivity/specificity of diagnosis for MPS is needed. The aims of this scoping review are to i) outline the clinical features of FM and MPS, ii) assess the current diagnostic protocols, iii) address inconsistency among health care practitioners’ diagnoses, iv) address alternative diagnostic tools and v) discuss the future application of a clinical decision rule for MPS.

Methods: The PubMed database was searched for the following key terms; ‘Myofascial Pain Syndrome’, ‘Fibromyalgia’, ‘Trigger Points’, and ‘Tender Points’. These terms were further combined with; ‘Classification’, ‘Diagnosis’, ‘Prevalence’, and ‘Epidemiology’. Inclusion was determined based on relevance to the 5 aims of the scoping review.

Results: Sixty-three articles were included in the analysis. Significant overlap exists between FM and MPS for both clinical presentation and diagnostic criteria. Two-thirds of pain patients are misdiagnosed as having FM (Fitzcharles & Boulos, 2003), however the proportion of those patients who exhibit MPS is unknown.

Discussion/Conclusions: The development of a clinical decision rule for the diagnosis of MPS will enable practitioners to reliably and consistently distinguish between MPS vs FM. This is urgently needed given that the treatment protocols for MPS and FM differ significantly.

## Dissociation between morphine-induced spinal gliosis and analgesic tolerance by ultra-low dose α_2_-adrenergic and cannabinoid CB_1_ receptor antagonists

Patrick Grenier^a^, David Wiercigroch^a^, Mary C Olmstead^b^, and Catherine M Cahill^c^

^a^Biomedical & Molecular Sciences, Queen’s University, Kingston, Ontario, Canada; ^b^Psychology, Queen’s University, Kingston, Ontario, Canada; ^c^Anesthesiology & Perioperative Care, Pharmacology, University of California, Irvine, Irvine, CA, USA

**CONTACT** Patrick Grenier 8pg3@queensu.ca

© 2017 Patrick Grenier, David Wiercigroch, Mary C Olmstead, and Catherine M Cahill. Published with license by Taylor & Francis.

This is an Open Access article distributed under the terms of the Creative Commons Attribution License (http://creativecommons.org/licenses/by/4.0/), which permits unrestricted use, distribution, and reproduction in any medium, provided the original work is properly cited.

Introduction/Aim: Opioid analgesic use is limited by development of tolerance and increasing the dose exacerbates side effects. Spinal glial activation contributes to tolerance as astrocytes and microglia shift to a pro-inflammatory phenotype. Spinal administration of ultra-low dose (ULD) G-protein-coupled receptor (GPCR) antagonists including opioid and α_2_-adrenergic receptor (AR) antagonists paradoxically enhance morphine effectiveness and attenuate loss of potency. Here, we determined whether *systemic* ULD α_2_-AR antagonists atipamezole or efaroxan attenuate tolerance development, whether these paradoxical effects extend to another GPCR family: the cannabinoid (CB_1_) receptor, and if changes in spinal gliosis explain their mechanism of action.

Methods: Male rats were treated daily with morphine (5mg/kg) alone or in combination with ULD α_2_-AR (atipamezole, efaroxan) or CB_1_ (rimonabant) antagonists (5ng/kg); control groups received ULD injections only. Thermal tail flick latencies were assessed across seven days, before and 30 minutes post-injection. On day eight, spinal cords were isolated and changes in spinal gliosis were assessed through fluorescent immunohistochemistry.

Results: Both ULD α_2_-AR antagonists attenuated morphine tolerance, whereas the ULD CB_1_ antagonist did not. In contrast, both ULD atipamezole and ULD rimonabant attenuated morphine-induced micro- and astrogliosis in deep and superficial spinal dorsal horn.

Discussion/Conclusions: While both ULD atipamezole and ULD rimonabant attenuated chronic morphine-induced spinal gliosis, only the α_2_-AR antagonists attenuated tolerance. While the paradoxical effects of ULD GPCR antagonists are common to several receptor systems associated with pain and reward, their mechanisms may differ and effects on spinal glia alone may not be the main mechanism through which tolerance is modulated.

## A genetic polymorphism in the dopa-decarboxylase gene associated with somatic symptoms in chronic pain reduces its enzymatic activity

Marjo Piltonen^a^, Samar Khoury^a^, Alexander Samoshkin^a^, Roger B. Fillingim^b^, J. D. Greenspan^c^, Richard Ohrbach^d^, Gary D. Slade^e^, Shad B. Smith^f^, William Maixner^f^, and Luda Diatchenko^a^

^a^The Alan Edwards Centre for Research on Pain and Faculty of Dentistry, McGill University, Montreal, QC, Canada; ^b^Department of Community Dentistry and Behavioral Science, University of Florida, Gainsville, FL, USA; ^c^Department of Neural and Pain Sciences, University of Maryland School of Dentistry, Baltimore, MD, USA; ^d^Department of Oral Diagnostic Services, University at Buffalo, Buffalo, NY, USA; ^e^Center for Pain Research and Innovation, University of North Carolina at Chapel Hill, Chapel Hill, NC, USA; ^f^Center for Translational Pain Medicine, Duke University, Durham, NC, USA

**CONTACT** Marjo Piltonen marjo.piltonen@mail.mcgill.ca

© 2017 Marjo Piltonen, Samar Khoury, Alexander Samoshkin, Roger B. Fillingim, Joel D. Greenspan, Richard Ohrbach, Gary D. Slade, Shad B. Smith, William Maixner, and Luda Diatchenko. Published with license by Taylor & Francis.

This is an Open Access article distributed under the terms of the Creative Commons Attribution License (http://creativecommons.org/licenses/by/3.0/), which permits unrestricted use, distribution, and reproduction in any medium, provided the original work is properly cited. The moral rights of the named author(s) have been asserted.

Introduction/Aim: Somatic symptoms aside from pain are elevated in chronic pain patients relative to pain-free controls. We identified SNP rs11575542 associated with frequency of somatic symptoms assessed with the Pennebaker Inventory of Limbic Languidness (PILL) questionnaire in a community-based cohort of temporomandidular disorder (Orofacial Pain Prospective Evaluation and Risk Assessment, OPPERA). This polymorphism is a non-synonymous missense variant (1385G>A, arginine to glutamine) located in the dopa-decarboxylase (DDC) gene. The current study was designed to identify the molecular functional effect of variant allele on the enzymatic activity of DDC.

Methods: DDC variants (DDCwt=allele G/arginine; DDCmut=allele A/glutamine) were cloned into pcDNA3.1-vectors and transfected to HEK293-cells. Lysates were prepared for western immunoblotting and enzymatic activity assays. 5-HTP and L-DOPA were used as substrates and the decarboxylation products were determined with UHPLC with a coulometric array detector. Michaelis-Menten parameters (Km, Vmax) were obtained from full kinetic curves.

Results: Expression levels of DDCwt and DDCmut were consistently similar. We observed a significant 20-23% reduction in the Vmax of the DDCmut for both 5-HTP and L-DOPA, but Km values did not differ significantly.

Discussion/Conclusions: Our results show that the functional effect of rs11575542 is reflected in the DDC enzymatic activity such that the mutant enzyme has a lower maximum kinetic velocity. This finding is likely very relevant to somatic symptoms as this mutation, associated with higher somatic scores, also reduces enzymatic efficacy.

## Comparison of treatment outcomes between morphine and concomitant morphine and clonidine regimens for neonatal abstinence syndrome

Courtney Gullickson^a^, Marsha Campbell-Yeo^b^, and Stefan Kuhle^c^

^a^Faculty of Medicine, Dalhousie University, Halifax, Nova Scotia, Canada; ^b^Department of Pediatrics, School of Nursing, Dalhousie University, Halifax, Nova Scotia, Canada; ^c^Department of Obstetrics & Gynecology, Dalhousie University, Halifax, Nova Scotia, Canada

**CONTACT** Courtney Gullickson courtney.gullickson@dal.ca

© 2017 Courtney Gullickson, Marsha Campbell-Yeo, and Stefan Kuhle. Published with license by Taylor & Francis.

This is an Open Access article distributed under the terms of the Creative Commons Attribution License (http://creativecommons.org/licenses/by/3.0/), which permits unrestricted use, distribution, and reproduction in any medium, provided the original work is properly cited. The moral rights of the named author(s) have been asserted.

Introduction/Aim: In 2010, the tertiary-care center involved in this study adjusted the treatment guidelines for neonatal abstinence syndrome (NAS) to include concomitant clonidine and morphine administration as first-line therapy. This study is the first evaluation of this practice change and aimed to compare treatment outcomes between the morphine alone and morphine + clonidine regimen for NAS in a neonatal intensive care unit from 2006-2015.

Methods: Using a retrospective population-based cohort design, infants treated pharmacologically for NAS delivered between 2006-2015, were identified using the Nova Scotia Atlee Perinatal Database. Treatment information was collected using chart review.

Results: The incidence of NAS increased from 0.79 per 1000 live births in 2007 to 4.00 per 1000 live births in 2015. The expected change over time due to adjusted guidelines was observed with 91% of infants from 2010-2015 being treated with morphine + clonidine. Of the 188 infants identified, a significantly longer length of treatment (*p*=0.004) and higher peak morphine dose (*p*=0.045) was observed in the morphine + clonidine group. Higher cumulative morphine exposure was also observed with combination therapy (*p*=0.228). The clinical factors of gestational age, weight, sex, and maternal smoking did not control for the differences seen between groups.

Discussion/Conclusions: The increase in length of treatment and morphine dose seen in the morphine + clonidine group was unexpected, as our findings contrasted with previous work on this treatment combination. Further exploration examining the impact of additional clinical characteristics is warranted such as maternal methadone and antidepressant exposure.

## The role of *TRPV1* single nucleotide polymorphisms in acute and chronic pain

Katerina Zorina-Lichtenwalter^a^, Marc Parisien^a^, Gary D. Slade^b^, Ronald Dubner^c^, Roger B. Fillingim^d^, Joel Greenspan^e^, Richard Ohrbach^f^, Charlie Knott^g^, William Maixner^h^, Man-Kyo Chung^i^, and Luda Diatchenko^a^

^a^Alan Edwards Centre for Research on Pain, McGill University, Montreal, Quebec, Canada; ^b^Regional Centre for Neurosensory Disorders, University of North Carolina, Chapel Hill, North Carolina, USA; ^c^Departments of Oral and Maxillofacial Surgery and Neural and Pain Sciences, University of Maryland, Baltimore, Maryland, USA; ^d^Department of Community Dentistry and Behavioral Science, University of Florida, Gainesville, Florida, USA; ^e^Department of Neural and Pain Sciences, University of Baltimore, Baltimore, Maryland, USA; ^f^Department of Oral Diagnostic Sciences, University of Buffalo, Buffalo, New York, USA; ^g^International, Social, Statistical,and Environment Sciences Survey Research Division, RTI, Durham, North Carolina, USA; ^h^Anesthesiology, Duke University, Durham, North Carolina, USA; ^i^Neural and Pain Sciences, University of Maryland, Baltimore, Maryland, USA

**CONTACT** Katerina Zorina-Lichtenwalter katerina.lichtenwalter@mail.mcgill.ca

© 2017 Katerina Zorina-Lichtenwalter, Marc Parisien, Gary D. Slade, Ronald Dubner, Roger B. Fillingim, Joel Greenspan, Richard Ohrbach, Charlie Knott, William Maixner, Man-Kyo Chung, and Luda Diatchenko. Published with license by Taylor & Francis.

This is an Open Access article distributed under the terms of the Creative Commons Attribution License (http://creativecommons.org/licenses/by/3.0/), which permits unrestricted use, distribution, and reproduction in any medium, provided the original work is properly cited. The moral rights of the named author(s) have been asserted.

Introduction/Aim: Transient receptor potential vanilloid-1, encoded by *TRPV1*, responds to a variety of danger signals, including heat and acidity, and initiates nociception. The genetic variants of *TRPV1* modulate pain perception in a multi-faceted manner. Previous reports have suggested divergent effects of a *TRPV1* variant in acute pain and osteoarthritis. Here we test common nonsynonymous single nucleotide polymorphisms (SNPs) in *TRPV1* for association with acute and chronic pain conditions in a discovery cohort and two replication cohorts.

Methods: We genotyped five common nonsynonymous *TRPV1* SNPs in a prospective cohort of 3200 participants as part of the Orofacial Pain: Prospective Evaluation and Risk Assessment (OPPERA) study. Participants underwent a clinical exam and quantitative sensory testing (QST), and incident TMD cases were identified. We attempted replication of initial association results in (1) a TMD case-control cohort of 400 genotyped Caucasian females and (2) 250,000 participants of the U.K. BioBank (UKBB) cohort. *TRPV1* SNPs were analysed for association with TMD and QST measures in the OPPERA and TMD cohorts and with chronic pain phenotypes in OPPERA and UKBB.

Results: Allele G of SNP rs55916885 (Q85R) is associated with increased sensitivity to heat in both OPPERA and TMD cohorts. By contrast, it is not associated with any tested chronic pain phenotype in OPPERA or UKBB.

Discussion/Conclusions: The minor allele of rs55916885 appears to increase the risk of heat sensitivity in *TRPV1* but not modulate the risk of chronic pain conditions in the tested cohorts.

## Mechanical pain threshold assessments: How many flights of stairs do we need to climb?

Lukas D. Linde, Tess E. Kruspe, Sasha V. Monteiro, and John Z. Srbely

Human Health and Nutritional Sciences, University of Guelph, Guelph, Ontario, Canada

**CONTACT** Lukas D. Linde llinde@uoguelph.ca

© 2017 Lukas D. Linde, Tess E. Kruspe, Sasha V. Monteiro, and John Z. Srbely. Published with license by Taylor & Francis.

This is an Open Access article distributed under the terms of the Creative Commons Attribution License (http://creativecommons.org/licenses/by/4.0/), which permits unrestricted use, distribution, and reproduction in any medium, provided the original work is properly cited.

Introduction/Aim: Mechanical pain threshold (MPT) is a valuable tool in the assessment of chronic pain (Rolke et al. 2007). A MPT assessment involves calculating a geometric mean of 5 ascending/descending staircases of weighed pinprick stimuli (Rolke et al. 2007), however, this can be time consuming. Reducing the number of staircases within MPT assessments would improve clinical feasibility of the technique. Our purpose was to determine the fewest number of staircases in a MPT assessment necessary to reproduce the MPT reading from the 5-staircase method. We hypothesized that fewer than 5 staircases were needed to provide an accurate and reproducible MPT.

Methods: Thirty-nine young healthy participants (21.82±2.27years) were exposed to one MPT assessment during one session. Five staircases were used in all MPT assessments. Nineteen participants completed a second MPT assessment following a 30-minute break and a third MPT assessment one week later to assess within and between day repeatability respectively. MPT was calculated using 5, 4, 3, 2, and 1 set(s) of ascending/descending pinprick staircases. Differences were compared using a one-way ANOVA. Within and between day intra-class correlation coefficients (ICC) assessed repeatability for MPTs calculated from respective staircase numbers.

Results: No significant differences between MPT values were observed when using different staircase numbers (F.Stat: 0.017, p-value: 0.999; 193.10± 22.66mN, 187.23±22.75mN, 185.21±23.21mN, 186.65±23.99mN, 186.49±24.34mN, 5 to 1 staircases respectively). Within and between-day ICCs revealed excellent reproducibility across all MPT staircase numbers (Range: 0.902-0.974).

Discussion/Conclusions: MPTs calculated from one staircase of pinprick stimuli provides the same accuracy and reproducibility as 5 staircases.

## Pain in severe dementia: A comparison of a fine-grained assessment approach to an observational checklist designed for clinical settings

Thomas Hadjistavropoulos^a^, Erin Browne^a^, Kenneth Prkachin^b^, Babak Taati^c^, and Ahmed Ashraf^c^

^a^Psychology and Centre on Aging and Health, University of Regina, Regina, SK, Canada; ^b^Psychology, University of Northern British Columbia, Prince George, BC, Canada; ^c^Toronto Rehabilitation Institute, University Health Network, Toronto, Ontario, Canada

CONTACT Thomas Hadjistavropoulos hadjistt@uregina.ca

© Thomas Hadjistavropoulos, Erin Browne, Kenneth Prkachin, Babak Taati, and Ahmed Ashraf. Published with license by Taylor & Francis.

This is an Open Access article distributed under the terms of the Creative Commons Attribution License (http://creativecommons.org/licenses/by/4.0/), which permits unrestricted use, distribution, and reproduction in any medium, provided the original work is properly cited.

Introduction/Aim: Fine grained observational approaches to pain assessment (e.g., the Facial Action Coding System; FACS) have been used extensively in research to evaluate pain responses in individuals with and without dementia. Such fine grained approaches are difficult to use in clinical settings as they require specialized training and equipment. For this reason, easy-to-use observational approaches (e.g., the Pain Assessment Checklist for Limited Ability to Communicate-II; PACSLAC-II) that do not require specialized training have been developed for clinical settings. Our goal was to directly compare the utility of a FACS-based fine grained system to the PACSLAC-II in differentiating painful from non-painful states in older adults with and without dementia.

Methods: We video-recorded 52 older adults with dementia (residing in long-term care) and 48 older adult outpatients without dementia, attending a physiotherapy clinic, during baseline conditions and while they took part in a standardized physiotherapy examination designed to identify painful areas. Videos were reliably coded using well established pain-related facial responses based on the FACS. They were also coded using the pain behaviours of the PACSLAC-II.

Results: Both tools reliably differentiated between painful and non-painful states, but the PACSLAC-II accounted for a greater portion of the variance than the fine-grained FACS-based approach. Participants with dementia obtained higher scores on the PACSLAC-II than participants without dementia.

Discussion/Conclusions: The results suggest that easy-to-use observational approaches for clinical settings are valid and that there may not be any clinically important advantages to using more resource intensive coding approaches based on FACS.

## Chronic pain in couples: Contextual influences on pain responses

Michelle M. Gagnon^a^, Thomas Hadjistavropoulos^b^, and Ying C. MacNab^c^

^a^Department of Psychology, University of Saskatchewan, Saskatoon, Saskatchewan, Canada; ^b^Department of Psychology, University of Regina, Regina, Saskatchewan, Canada; ^c^School of Population and Public Health, University of British Columbia, Vancouver, British Columbia, Canada

**CONTACT** Michelle M. Gagnon michelle.gagnon@usask.ca

© Michelle M. Gagnon, Thomas Hadjistavropoulos, and Ying C. MacNab. Published with license by Taylor & Francis.

This is an Open Access article distributed under the terms of the Creative Commons Attribution License (http://creativecommons.org/licenses/by/4.0/), which permits unrestricted use, distribution, and reproduction in any medium, provided the original work is properly cited.

Introduction/Aim: This is an experimental study of pain communication in couples. Despite evidence that chronic pain in one partner impacts both members of the dyad, dyadic influences on pain communication have not been sufficiently examined and are typically based on retrospective report. Our goal was to directly study contextual influences (i.e., presence of chronic pain, relationship quality, and pain catastrophizing) on self-reported and non-verbal (i.e., facial expressions) pain responses.

Methods: Couples with (*n* = 66) and without (*n* = 65) an individual with chronic pain (ICP) completed relationship and pain catastrophizing questionnaires. Subsequently, one partner underwent a pain task (pain target, PT), while the other partner observed (pain observer, PO). In couples with an ICP, the ICP was assigned to be the PT. Pain intensity and perceived pain intensity ratings were recorded at multiple intervals and facial expressions were video-recorded throughout the pain task. Pain-related facial expression was quantified using the Facial Action Coding System. Facial expressions of emotions were measured using specialized computer vision software.

Results: Relationship variables in POs and catastrophizing in PTs interacted with the presence/absence of chronic pain to influence pain-related facial expressions, but not self-reported pain. PTs provided higher pain ratings than POs and female PTs reported and showed more pain, regardless of chronic pain status. Facial expressions of emotions occurring between partners differed by group.

Discussion/Conclusions: Contextual variables influence pain communication in couples, and PTs and POs are influenced by distinct variables. Self-report and non-verbal responses are not displayed in parallel manners.

## The role of meaning in life in adjustment to a chronic medical condition

Umair Majid^a^, Jeffrey Ennis^b^, Malika Bhola^c^

^a^Department of Health Research Methods, Evidence and Impact (HEI) - formerly known as Clinical Epidemiology and Biostatistics, McMaster University, Hamilton, Ontario, Canada; ^b^Department of Physical Rehabilitation Medicine, McMaster University & Ennis Centre for Pain Management, Hamilton, Ontario, Canada; ^c^School of Interdisciplinary Science, McMaster University, Hamilton, Ontario, Canada

**CONTACT** Umair Majid majidua@mcmaster.ca

© Umair Majid, Jeffrey Ennis, and Malika Bhola. Published with license by Taylor & Francis.

This is an Open Access article distributed under the terms of the Creative Commons Attribution License (http://creativecommons.org/licenses/by/4.0/), which permits unrestricted use, distribution, and reproduction in any medium, provided the original work is properly cited.

Introduction/Aim: Being diagnosed with a chronic disease can cause significant distress. Such an event can disrupt an individual’s understanding of their ‘meaning in life’; their purpose. This can initiate an active search for new purpose in order to reduce the distress, and improve adjustment to medical disease. This paper reviews the literature to describe the multiple life changes associated with the diagnosis of a chronic medical disease and to create a framework for understanding the relationship between Meaning in Life and these changes.

Methods: A database search using keywords and subject headings was performed in 5 databases. After sorting according to predetermined criteria, 46 papers were included in the present review.

Results: *Adjustment* consists of the psychological, physical and social changes that occur after the diagnosis of a chronic medical condition or trauma. This causes an *active search for meaning* in an attempt to restore the patient’s sense of purpose. The act of searching and finding new meaning in life is associated with positive adjustment outcomes. However, searching without success can have a negative impact on adjustment and quality of life.

Discussion/Conclusions: A diagnosis of a chronic medical condition causes psychological, physical and/or social distress, which can have a negative impact on an individual’s sense of purpose, or Meaning in Life. This preliminary review of the literature describes the process of searching for Meaning in Life, and explores its consequences on adjustment to chronic medical condition after diagnosis. Future recommendations for the direction of research in this area will be provided.

## The influence of parent-child reminiscing about post-surgical pain on children’s pain memory development: A longitudinal examination

Maria Pavlova^a^, Lauren McCallum^a^, Jill Vinall^b^, and Melanie Noel^c^

^a^Psychology, University of Calgary, Calgary, Alberta, Canada; ^b^Anaesthesia, University of Calgary, Calgary, Alberta, Canada; ^c^Alberta Children’s Hospital Research Institute, Calgary, Alberta, Canada

**CONTACT** Maria Pavlova mpavlova@ucalgary.ca

© 2017 Maria Pavlova, Lauren McCallum, Jill Vinall, and Melanie Noel. Published with license by Taylor & Francis.

This is an Open Access article distributed under the terms of the Creative Commons Attribution License (http://creativecommons.org/licenses/by/4.0/), which permits unrestricted use, distribution, and reproduction in any medium, provided the original work is properly cited.

Introduction/Aim: Children’s memories for pain are a powerful mechanism underlying pain trajectories. Parent-child interactions have been posited to play an important role in shaping pain memory biases, particularly among young children. Parent-child reminiscing about past negative events creates a powerful sociolinguistic context that shapes children’s memory. Previous studies have identified adaptive ways to reminisce with children: using elaborative questions and emotion-laden words is associated with more accurate recall. However, no studies have yet examined how parents and children reminisce about post-surgical pain and how it may influence subsequent pain memories. Using a longitudinal design, we investigated the reminiscing style of parent-child surgery-related narratives and their influence on children’s post-surgical pain recall.

Methods: To date, 45, 4- to 7-year old children (20 girls, mean age = 5.31) and their parents reported pain (the Faces Pain Scale-Revised, FPS-R) after tonsillectomy, a common outpatient pediatric surgery associated with high levels of post-surgical pain. Two weeks post-surgery, children and parents came to the lab and engaged in a narrative elicitation task wherein they reminisced about the surgery experience. Narratives were coded using an established coding scheme drawn from the developmental literature. One month post-surgery, children recalled their post-surgical pain using the FPS-R.

Results: Findings revealed that more elaborative parental reminiscing (*r* = -.30) and more frequent use of explanations (*r* = ‒.26) and emotion-laden words (*r* = ‒.34) were associated with less distressing children’s pain memories.

Discussion/Conclusions: Reminiscing with children about post-surgical pain using elaborations and emotion-rich language might help them to develop less distressing pain memories, thus positively altering pain trajectories.

## Pain management interventions in the pediatric intensive care unit (picu): A scoping review

Ahmad Ismail^a^, Paula Forgeron^a^, Viola Polomeno^a^, Huda Gharaibeh^b^, and Denise Harrison 0000-0001-7549-7742^a^

^a^Nursing, University of Ottawa, Ottawa, ON, Canada; ^b^Nursing, Jordan University of Science and Technology, Irbid, Jordan

**CONTACT** Ahmad Ismail aisma089@uottawa.ca

© 2017 Ahmad Ismail, Paula Forgeron, Viola Polomeno, Huda Gharaibeh, and Denise Harrison. Published with license by Taylor & Francis.

This is an Open Access article distributed under the terms of the Creative Commons Attribution License (http://creativecommons.org/licenses/by/4.0/), which permits unrestricted use, distribution, and reproduction in any medium, provided the original work is properly cited.

Introduction/Aim: A synthesis of research focused on pain management interventions in the Pediatric Intensive Care Unit (PICU) is needed. We aim to identify research based pain management interventions used in the PICU.

Methods: Arksey and O’Malley (2005) framework guided this review. We searched CINAHL, EMBASE, MEDLINE, PsychINFO, and ProQuest Dissertations & Theses Global (from the date of inception to 2015) and reference lists. Primary research articles focused on pain management interventions used in the PICU were included. Two independent reviewers conducted titles and abstracts screening, full text screening, and data extraction. A third reviewer resolved any disagreement.

Results: 7047 articles were identified, 100 underwent full text screening and 27 studies were included in the final review (25 from the search and two from reference lists). Sixteen articles (59%) were non-experimental studies, and 11 (41%) were experimental, of which 8 were randomized controlled trials (RCTs). Interventions were categorized into: Pharmacological, physical, psychological, and others. The majority of the articles solely focused on pharmacological interventions (n= 21, 78%), one on physical, and one on psychological interventions. Four studies included more than one category of interventions. The majority of the studies focused on post-operative pain management (n=18, 67%), two (7%) on analgesia and sedation management, and seven (26%) on other pain management for different conditions.

Discussion/Conclusions: The majority were non clinical trials focusing on medications and post-operative pain management. More research, especially clinical trials, is warranted to determine the most effective non-pharmacological (physical and psychological) interventions.

## Associations between benefit finding and pain-related constructs in adolescents with chronic pain

Sabine Soltani, Maria Pavlova, and Melanie Noel

Psychology, University of Calgary, Calgary, Alberta, Canada

**CONTACT** Sabine Soltani ssoltani@ucalgary.ca

© Sabine Soltani, Maria Pavlova, and Melanie Noel. Published with license by Taylor & Francis.

This is an Open Access article distributed under the terms of the Creative Commons Attribution License (http://creativecommons.org/licenses/by/4.0/), which permits unrestricted use, distribution, and reproduction in any medium, provided the original work is properly cited.

Introduction/Aim: In contrast to burgeoning literature on risk factors and negative outcomes associated with pediatric chronic pain, there is a paucity of research examining resilience factors implicated in adaptive functioning and positive outcomes^1,2^. Resilience factors that have been examined include self-efficacy^3,4^, acceptance^5,6^, and optimism^7^. Benefit finding (BF) is characterized by positive changes or gains in the face of significant adversity^7,8^ and has been identified as a potential resilience outcome in risk-resilience conceptual models of chronic pain^9,10^. Much of the existing research examining the role of BF in pediatric health conditions has assessed children diagnosed with cancer, revealing positive relationships with adaptive outcomes^11^. This is the first study to examine associations between BF and pain-related variables in a sample of adolescents with chronic pain.

Methods: Youth (*n* = 118, 65% = girls, *M*_age_ = 13.43) were assessed at the point of entry into a tertiary, interdisciplinary pediatric chronic pain program and completed psychometrically sound measures assessing pain characteristics, pain catastrophizing, and BF (adapted for the chronic pain context) as part of a multi-wave outcome initiative.

Results: Hierarchical regression analyses revealed that higher BF predicted higher pain intensity (*β* = .32, *R^2^* = .10), pain interference (*β* = .32, *R^2^* = .20), and pain catastrophizing (*β* = .35, *R^2^* = .16) when controlling for age and sex (*p*s < .01).

Discussion/Conclusions: In contrast to other chronic illness populations, BF was related to worse outcomes, suggesting that while benefit is perceived among youth who are severely affected by pain, this construct operates differently in this population. Future research is needed to examine BF as a predictor of treatment response and long-term outcomes.

### References

1)Hilliard et al., 2015

2)Huguet et al., 2011

3)Carpino, Segal, Logan, Lebel, & Simons, 2014

4)Kalapurakkel et al., 2014

5)McCracken & Morley, 2014

6)Weiss et al., 2013

7)Cousins et al., 2015

8)Phipps, Long, & Ogden, 2007

9)Cousins et al., 2010

10)Sturgeon & Zautra, 2013

11)Currier, Hermes, & Phipps, 2009

## Parental emotional availability while reminiscing about post-surgical pain contributes to children’s pain memory development

Jillian Vinall^a^, Lauren McCallum^b^, Maria Pavolva^b^, Nivez Rasic^a^, and Melanie Noel^b^

^a^Department of Anesthesiology; ^b^Department of Psychology, University of Calgary, Calgary, AB, Canada

**CONTACT** Jillian Vinall, PhDjillian.vinall@ahs.ca

© Jillian Vinall, PhD, Lauren McCallum, BA, Maria Pavolva, MSc, Nivez Rasic, MD, FRCPC, Melanie Noel, PhD. Published with license by Taylor & Francis.

This is an Open Access article distributed under the terms of the Creative Commons Attribution License (http://creativecommons.org/licenses/by/3.0/), which permits unrestricted use, distribution, and reproduction in any medium, provided the original work is properly cited. The moral rights of the named author(s) have been asserted.

Introduction/Aim: Memory for pain is a robust predictor of worse pain and distress during subsequent pain experiences. Parent anxiety has been linked to child pain memory, and parent emotional availability (EA) is the strongest predictor of child anticipatory distress for future painful procedures. Parental involvement has been hypothesized as being important to pain memories, however, the extent to which parent-child EA contributes to child pain memory is unknown. We examined whether parent-child EA is associated with child memories for pain 1-month post-surgery.

Methods: To-date, we recruited 27 children aged 4-7 years (mean=5.5), undergoing a tonsillectomy procedure. Parents filled out the State-Trait Anxiety Questionnaire to assess their own anxiety. Children reported their post-surgical pain using the Faces Pain Scale-Revised (FPS-R). Two weeks following the surgery, parent-child dyads came to the lab and reminisced about the surgical experience using a structured elicitation task. EA was assessed during this reminiscing task using the EA Scale. One-month post-surgery, children recalled their pain using the FPS-R.

Results: Children of dyads who had greater ratings of parent-child EA (p=0.02) developed less distressing pain memories 1-month post-surgery (adjusted R^2^=0.22) compared to children of dyads with lower parent-child EA, after accounting for child age and sex, post-surgical pain, socio-economic status, and parent state-trait anxiety (all n.s.).

Discussion/Conclusions: Parents who are EA to their child, may have the opportunity to reframe their child’s memory for pain, so that they remember it more accurately/positively; thereby, potentially decreasing the likelihood that their child will be distressed and avoidant of future painful procedures.

## Interprofessional graduate level pain education offered through a university certificate in pain management program

Shawn Drefs, Judith Hunter, and Bernadette Martin

Faculty of Rehabilitation Medicine, University of Alberta, Edmonton, AB, Canada

**CONTACT** Shawn Drefs sdrefs@ualberta.ca

© Shawn Drefs, Judith Hunter, and Bernadette Martin. Published with license by Taylor & Francis.

This is an Open Access article distributed under the terms of the Creative Commons Attribution License (http://creativecommons.org/licenses/by/4.0/), which permits unrestricted use, distribution, and reproduction in any medium, provided the original work is properly cited.

Introduction/Aim: In 2010 the University of Alberta’s Faculty of Rehabilitation Medicine developed a Certificate in Pain Management program. Guiding principles for development were; graduate level, easily accessible, focus on adult and IPE learning strategies, use of latest in online learning technologies, incorporation of synchronous learning strategies, interdisciplinary focus, evidence based and high clinical relevance.

Methods: A Certificate Development Committee (CDC) consisting of a mix of academics, public health administrators, and clinicians were responsible for initial curriculum development. In addition to instruction by a pain expert, a recorded lecture format has been used to include leaders in pain research, management, and education.

Results: The certificate consists of three courses offered in a distance based format. Courses consist of asynchronous and synchronous learning activities. Graduate level credit earned may be used as elective credit for clinicians pursuing graduate studies. Physician participants may obtain MAINPRO Cert+ credits through the College of Family Physicians of Canada (CFPC) and courses may be used towards credentialing through the Canadian Academy of Pain Management (CAPM). Pain certificate program registrants represent a diverse mix of clinicians including physicians, pharmacists, physical therapists, occupational therapists, psychologists, nurses, social workers, and others. Since May 2010, over 150 clinicians have registered for this certificate program.

Discussion/Conclusions: Post-course quantitative and qualitative participant surveys reveal high levels of satisfaction and applicability to practice. To date post-course evaluation has consisted of student evaluations and a post (1.5 years) program completion evaluation. Data collected has helped determine level of knowledge translation in the clinical setting.

## Cannabis for management of chronic pain within the expanded medical marihuana for medical purposes program of health canada: Results of a survey

Mary E. Lynch^a^, and Myrna Yazer^b^

^a^Anesthesiology, Pain Management and Perioperative Medicine, Dalhousie University, Halifax, Nova Scotia, Canada; ^b^Anesthesiology, Pain Management and Perioperative Medicine, Queen Elizabeth II Health Sciences Center, Halifax, Nova Scotia, Canada

**CONTACT** Mary E Lynch mary.lynch@dal.ca

© Mary E Lynch and Myrna Yaze. Published with license by Taylor & Francis.

This is an Open Access article distributed under the terms of the Creative Commons Attribution License (http://creativecommons.org/licenses/by/4.0/), which permits unrestricted use, distribution, and reproduction in any medium, provided the original work is properly cited.

Introduction/Aim: The objective of this survey was to collect information regarding patient experience with using medical cannabis for chronic pain under the expanded program for medical cannabis which allowed patients to purchase varying strains of cannabis from a variety of producers (launched in June 2013).

Methods: This was a pragmatic survey study of a consecutive group of patients who were using cannabis as a part of their pain management in the context of a tertiary care pain clinic. The survey was given to patients known to be using medical cannabis who presented for follow-up between April and September of 2015. Patients were asked to complete the survey and return it to the clinic. Information regarding type of pain or other symptoms as well as dose, route of administration, side effects and products used was collected

Results: Fifty percent (N = 12) of surveys were returned completed. 10 participants reported pain to be slightly or much improved with additional improvements in sleep and other symptoms. All reported side effects, the most common were dry mouth and drowsiness. Most were using a smoked or vaporized route of delivery, the majority a dose of 3-4 puffs and an average daily dose of 1.75 grams.

Discussion/Conclusions: This survey supports that some patients using marihuana for chronic pain conditions report benefit in pain, sleep and other symptoms side effects were similar to those reported with other types of analgesic medications. There was not enough information to report on strain specific differences for specific pain diagnoses. Further research is needed.

## Dynamic biomechanics of epidural insertion

William P. McKay^a^, Andrew Frost^a^, Brendan Kushneriuk^a^, Jayden Cowan^a^, Andrew Wang^a^, Christopher Durr^a^, Tanner Lange^a^, and Rachel Guo^a^

Anesthesia, University of Saskatchewan, Saskatoon, Saskatchewan, Canada

**CONTACT** William P. McKay bill.mckay@usask.ca

© 2017 William P. McKay, Andrew Frost, Brendan Kushneriuk, Jayden Cowan, Andrew Wang, Christopher Durr, Tanner Lange, and Rachel Guo. Published with license by Taylor & Francis.

This is an Open Access article distributed under the terms of the Creative Commons Attribution License (http://creativecommons.org/licenses/by/3.0/), which permits unrestricted use, distribution, and reproduction in any medium, provided the original work is properly cited. The moral rights of the named author(s) have been asserted.

Introduction: Epidural analgesia is frequently used with loss of resistance (LOR) to injection of air or saline for insertion. Dynamic biomechanics have not been studied. We measured pressure and flow during clinical epidural insertion.

Methods: Pressure was recorded with a sterile line attached between LOR syringe and epidural needle. Flow and injection times were measured every 0.1s from a high-resolution video recording.

Results: With Ethics approval, 19 patients having epidurals for postoperative pain control were recruited. Two declined and one had technical difficulties, leaving 16 receiving thoracic epidurals; 11 women and 5 men aged (mean ± SD) [minimum – maximum] 67 ± 9 years, 164 ± 10cm tall, and weighing 78 ± 15kg. Twelve had LOR of saline; 4 air. LOR measurements were highly variable: maximum injection pressures 394 ± 104torr [160-500]; flows 2.3 ± 1.4ml/s [0.4–5.4]; volume injected 3.25 ± 1.25ml [0.4-7.6]; injection duration 1.51 ± 0.68s [0.4-2.6]. Dispersion time 1.28 ± 0.71s was significantly longer than injecting into a saline bag 0.05 ± 0.006s (p = 0.0001).

Discussion/Conclusions: This technique of studying dynamic epidural biomechanics proved acceptable in this small pilot study, maintaining sterility while interfering minimally with epidural insertion. There is high inter-individual variability in LOR biomechanics, while demographic variability was small. Health quality experts reason that highly variable methods cannot all be best practice.. Pressure dispersion time (the time that pressure is elevated above baseline after injection) is a function of epidural tissue properties. Dispersion times suggest that the epidural space behaves as a poroelastic tissue. Further dynamic biomechanic research may suggest an optimal LOR volume and force that may lead to safer epidural insertion.

## Confirmatory factor analysis of the Sensitivity to Pain Traumatization Scale in postsurgical patients seen by the Transitional Pain Service

Samantha R. Fashler^a^, Janice Montbriand^b^, Aliza Weinrib^a,^^b^, Hance Clarke^b^, and Joel Katz^a,^^b^

^a^Department of Psychology, York University, Toronto, Ontario, Canada; ^b^Pain Research Unit, Department of Anesthesia and Pain Management, Toronto General Hospital, Toronto, Ontario, Canada

**CONTACT** Samantha R. Fashler sfashler@yorku.ca

© 2017 Samantha R. Fashler, Janice Montbriand, Aliza Weinrib, Hance Clarke, and Joel Katz. Published with license by Taylor & Francis.

This is an Open Access article distributed under the terms of the Creative Commons Attribution License (http://creativecommons.org/licenses/by/3.0/), which permits unrestricted use, distribution, and reproduction in any medium, provided the original work is properly cited. The moral rights of the named author(s) have been asserted.

Introduction/Aim: The aim of the present study was to examine the factor structure and psychometric properties of the 12-item Sensitivity to Pain Traumatization Scale (SPTS), a measure of the anxiety-related cognitive, emotional, and behavioral responses to pain that resemble a traumatic stress response.

Methods: A sample of 134 adults (50% female; *M_age_* = 50.06, *SD_age_* = 14.05) completed a questionnaire assessing symptoms of pain, anxiety, depression, and trauma at their first outpatient visit to the Transitional Pain Service after surgery. Confirmatory factor analysis was used to estimate a non-hierarchical, one-factor model of the SPTS.

Results: The one-factor model showed a good fit to the data, *χ*^2^(54) = 89.636, CFI = 0.943, TLI = 0.930, SRMR = 0.051, RMSEA = 0.070 (90% CI: 0.043-0.096). The completely standardized factor loadings ranged from .461 to .882 and items accounted for between 21.3%–77.7% of variance explained by the model. Internal consistency was excellent (α = 0.92). Construct validity was evaluated by examining the correlations of the total SPTS score with a similar construct [post-traumatic stress scores; *r*(117) = .620, *p* < .001]. Divergent validity was evaluated by examining the correlations of the total SPTS score and a dissimilar construct [depressive symptoms score; *r*(132) = .551, *p* < .001].

Discussion/Conclusions: The findings support the one-factor model and validity of the SPTS in postsurgical patients seen by the Transitional Pain Service.

## Uterine axon density is increased in the sub-serosal myometrium of women with endometriosis

Jane E. Girling^a^, Martin Healey^a^, Peter A.W. Rogers^a^, and Janet Keast^b^

^a^Gynaecology Research Centre, Department of Obstetrics and Gynaecology, The University of Melbourne and The Royal Women’s Hospital, Melbourne, Victoria, Australia; ^b^Department of Anatomy and Neuroscience, The University of Melbourne, Melbourne, Victoria, Australia

**CONTACT** Jane E. Girling jgirling@unimelb.edu.au

© 2017 Jane E. Girling, Martin Healey, Peter A.W. Rogers, and Janet Keast. Published with license by Taylor & Francis.

This is an Open Access article distributed under the terms of the Creative Commons Attribution License (http://creativecommons.org/licenses/by/3.0/), which permits unrestricted use, distribution, and reproduction in any medium, provided the original work is properly cited. The moral rights of the named author(s) have been asserted.

Introduction/Aim: We examined whether patterns of uterine innervation were related to pain specifically in women with endometriosis or more generally in women with menstrual pain.

Methods: Full thickness uterine samples were collected (n = 10) from women undergoing hysterectomy and classified based on presence/absence of self-reported endometriosis and pelvic pain severity. Tissues were fixed (4% paraformaldehyde), cryoprotected and frozen. Fluorescent immunostaining (16μm sections) was undertaken using CD31 (pan-endothelial) and either PGP9.5 (pan-neuronal) or tyrosine hydroxylase (TH, sympathetic axons) antibodies. Axon density was determined in different uterine regions by counting axon crossing points from an overlying grid (researcher blinded to patient details).

Results: PGP9.5 and TH-positive axons were concentrated in *sub-serosal* myometrium and their density was highly variable between and within samples. Endometrial axons were rare (observed in 4 samples, only one from a women reporting endometriosis) and largely restricted to the basalis. Axon density in the sub-serosal myometrium was increased in women with self-reported endometriosis relative to those without the disorder (No endometriosis vs. endometriosis: PGP9.5: median crossing points/grid [range] = 0[0,2] vs. 9[2,17], p = 0.02; TH: 1[0,2] vs. 3[2,9], p = 0.01). In this cohort, 9 of 10 women reported pain (visual analogue pain scores: 64-100); therefore, we were unable to correlate pain and axon density.

Discussion/Conclusions: Using optimised protocols, we demonstrate that axon density is increased in sub-serosal myometrium of women with endometriosis; future studies should consider how these patterns relate to pain symptoms. Unlike previous studies that suggested using endometrial biopsies for endometriosis diagnosis, we rarely observed endometrial axons.

## Speaking up about painful sex: Goals for disclosing pain are associated with women’s sexual functioning, relationship satisfaction, and depressive symptoms

Kathleen E. Merwin 0000-0002-9349-8009 ^a^, Lucia F. O’Sullivan^b^, Natalie O. Rosen ^a^

^a^Department of Psychology & Neuroscience, Dalhousie University, Halifax, Nova Scotia, Canada; ^b^Department of Psychology, University of New Brunswick, Fredericton, New Brunswick, Canada

**CONTACT** Kathleen E. Merwin kathleen.merwin@dal.ca

© 2017 Kathleen E. Merwin, Lucia F. O’Sullivan, and Natalie O. Rosen. Published with license by Taylor & Francis.

This is an Open Access article distributed under the terms of the Creative Commons Attribution License (http://creativecommons.org/licenses/by/3.0/), which permits unrestricted use, distribution, and reproduction in any medium, provided the original work is properly cited. The moral rights of the named author(s) have been asserted.

Introduction/Aim: A significant minority (14–34%) of young women experience pain during intercourse (van Lankveld et al., 2010), which is associated with higher depression, and lower sexual functioning and relationship satisfaction. For women who experience this pain, more open sexual communication is associated with greater psychological, sexual, and relationship well-being (Rancourt et al., 2016). However, little is known about the correlates of disclosing painful sex to romantic partners. Further, disclosure motivated by approach goals (seeking desirable outcomes, such as intimacy) may be beneficial, whereas avoidance goals (escaping undesirable outcomes, such as further sexual problems) may be detrimental (Chaudoir & Fisher, 2010). We examined whether disclosure (vs. non-disclosure) of painful sexual intercourse, as well as the goals for disclosure, were associated with women’s psychological, sexual, and relationship well-being.

Methods: Women (*N* = 272) completed standardized online measures assessing sexual problems, relationship satisfaction, sexual functioning, depressive symptoms, and goals for disclosing sexual problems.

Results: Women reporting pain during intercourse who had disclosed this problem to their current partner reported fewer depressive symptoms, and greater sexual functioning and relationship satisfaction compared to non-disclosers. When women endorsed stronger approach goals for disclosure, they also reported greater sexual functioning and relationship satisfaction, and fewer depressive symptoms, whereas when women had stronger avoidance goals they reported poorer sexual functioning and relationship satisfaction, and more depressive symptoms.

Discussion/Conclusions: Findings may inform clinical interventions by suggesting that disclosing painful sex, particularly when motivated by approach goals, may benefit women’s well-being by enhancing intimacy or allowing couples to adapt sexual activities to accommodate the pain.

## Taking care of complex regional pain syndrome: Overview of clinical reality in a tertiary pain clinic

Julie Steele^a^, and Anne M. Pinard^b^

^a^Anesthesiology and critical care department, Université Laval, Québec, Canada; ^b^Anesthesiology and critical care department, Université Laval, Chronic Pain Clinic, CHUL du CHU de Québec, Québec, Canada

**CONTACT** Julie Steele julie.steele.1@ulaval.ca

© 2017 Julie Steele and Anne Marie Pinard. Published with license by Taylor & Francis.

This is an Open Access article distributed under the terms of the Creative Commons Attribution License (http://creativecommons.org/licenses/by/3.0/), which permits unrestricted use, distribution, and reproduction in any medium, provided the original work is properly cited. The moral rights of the named author(s) have been asserted.

Introduction/Aim: The aim of the present study was to describe the progression of a cohort of patient with clinical diagnosis of CRPS followed in a tertiary pain clinic, and compare the evolution of our patients to the literature.

Methods: After approval of Research Ethics Board, 65 patients with clinical upper limb CRPS, followed between January 1^st^ 2014 and April 15^th^ 2016, were retrospectively studied. Using existing charts, patients were classified according to Budapest criteria. Demographic data, medical history, medication tried and interventional therapies (IT) received were assessed. Using bivariate logistic regression, these data were compared with outcome referring to pain reduction, hand function improvement and return to work.

Results: 92% suffered of type 1 CRPS. 26% were classified Not Otherwise Specified (NOS), but had similar evolution then the rest of the group. Patients had an average of 10 IT, most often intravenous regional anesthesia (IVRA) with passive mobilization given by occupational therapist (OT) (97%). 79% had subjective pain reduction, 81% had hand function improvement and 49% returned to work. Patients taking opioids returned less to work [O.R. 0.12, 95% C.I. [0.02–0.63] p 0.01]. Social security indemnity was associated with less pain reduction [O.R. 0.26, 95% C.I. [0.07–0.97] p 0.04].

Discussion/Conclusions: This study is significant for clinicians; it describes evolution of patients we treat everyday. Our cohort’s evolution is similar to the literature. We can infer the type of treatment (IVRA + OT) is as least as effective as other techniques; it is the first time this technique is described.

## Metacognitions may account for the relationship between chronic pain and health anxiety

Geoffrey S. Rachor and Alexander M. Penney

Psychology, MacEwan University, Edmonton, AB, Canada

**CONTACT** Geoffrey S. Rachor rachorg2@mymacewan.ca

© 2017 Geoffrey S. Rachor and Alexander M. Penney. Published with license by Taylor & Francis.

This is an Open Access article distributed under the terms of the Creative Commons Attribution License (http://creativecommons.org/licenses/by/3.0/), which permits unrestricted use, distribution, and reproduction in any medium, provided the original work is properly cited. The moral rights of the named author(s) have been asserted.

Introduction/Aim: Research has shown that health anxiety (HA) significantly contributes to disability in chronic pain populations. The aim of the current study was to examine if metacognitive beliefs may account for the relationship between chronic pain and HA.

Methods: University students who reported experiencing (*n* = 157), or not experiencing (*n* = 128), chronic or recurring pain completed the Chronic Pain Grade Scale (CPGS), Short Health Anxiety Inventory (SHAI), and Metacognitions about Health Questionnaire (MCQ-HA). Bivariate correlations were conducted to determine whether CPGS intensity and disability subscale scores correlated with SHAI scores. Partial correlations were conducted to examine whether these correlations remained significant when controlling for MCQ-HA scores.

Results: In the pain sample, bivariate correlations revealed that HA significantly correlated with disability, *r* = .20, but not with intensity, *r* = .13. Partial correlations controlling for metacognitive beliefs revealed that the relationship between HA and disability was no longer significant, *r* = .04. In the non-pain sample, bivariate correlations revealed that HA significantly correlated with both disability, *r* = .19, and intensity, *r* = .26. Partial correlations revealed that the relationship between HA and both intensity, *r* = .16, and disability, *r* = .02, were no longer significant when controlling for metacognitions.

Discussion/Conclusions: Metacognitions appear to contribute to the relationship between chronic pain and HA. These results have implications for the treatment of HA in chronic pain populations. Future research should examine the contributions of metacognitive beliefs in delineating the relationship between chronic pain and HA.

## Incorporating pain experiences into personal identity: Implications for romantic relationships and securing social support

Lyndsay Crump and D. L. LaChapelle

Psychology, University of New Brunswick, Fredericton, New Brunswick, Canada

**CONTACT** Lyndsay Crump lcrump@unb.ca

© 2017 Lyndsay Crump and Diane L. LaChapelle. Published with license by Taylor & Francis.

This is an Open Access article distributed under the terms of the Creative Commons Attribution License (http://creativecommons.org/licenses/by/3.0/), which permits unrestricted use, distribution, and reproduction in any medium, provided the original work is properly cited. The moral rights of the named author(s) have been asserted.

Introduction/Aim: Supportive romantic relationships can improve persons with chronic pains’ (CP) well-being; however, those affected are less likely to have a partner. This study explored barriers persons with CP experience while initiating/developing new romantic relationships. Methods: Twenty-two women and 18 men who were single or newly partnered (<6 months, non-cohabitating) provided demographic/health information then participated in a semi-structured interview. Participants were asked whether/how CP affected their dating experiences, expectations, and goals. Verbatim interview transcripts were analyzed using thematic analysis.

Results: Participants’ responses indicate identity reformation (a two-component process) is an overarching theme influencing how relationship barriers (e.g., CP stereotypes) are experienced. Dating barriers forced participants to recognize their inability to be the person they were before CP or who they ‘should’ be (*realizing I can’t be me)*. Participants subsequently began formulating a with-CP identity and assessing its value against prior identities (*evaluating with-CP me*). Some participants equated having CP to being shamefully flawed and unworthy of love and acceptance. These participants struggled to maintain their pre-CP selves or adopted a pain-focused identity. Alternatively, those who held neutral/positive beliefs about persons with CP were more willing and able to integrate pain as a part of their identity without shame, and described more success pursuing romantic relationships.

Discussion/Conclusions: When people view their with-CP identity as shameful, they are less willing and able to work towards initiating/developing a loving romantic relationship - a cherished life goal for many. These findings suggest positive with-CP identity development may be an important clinical target for helping-professionals.

## Competitiveness in facebook peer support groups for Fibromyalgia

Samantha Landry, Lyndsay Crump, and Diane LaChapelle^a^

Psychology, University of New Brunswick, Fredericton, New Brunswick, Canada

**CONTACT** Samantha Landry samantha.landry@unb.ca

© 2017 Samantha Landry, Lyndsay Crump, and Diane LaChapelle. Published with license by Taylor & Francis.

This is an Open Access article distributed under the terms of the Creative Commons Attribution License (http://creativecommons.org/licenses/by/3.0/), which permits unrestricted use, distribution, and reproduction in any medium, provided the original work is properly cited. The moral rights of the named author(s) have been asserted.

Introduction/Aim: Many people with FM participate in online support groups (OSG): they are accessible from home, facilitate information sharing, and ideally provide a safe space to seek/offer support. Peer-to-peer Fibromyalgia OSGs have become prolific, but existing research suggests they may be harmful. Further examination of the content is therefore warranted.

Methods: This project is part of a longitudinal study examining Facebook OSGs for FM. Posts from three separate Fibromyalgia Facebook groups were collected for one week per month for three consecutive months and analyzed according to the six steps of thematic analysis.

Results: Preliminary analyses identified *competitiveness* as a key response to support-seeking posts. It had two main components: (1) “I’m sicker than you” where participants describe experiencing more symptoms than previous posters which they equate with greater suffering; and (2) “I’m the Expert” where participants used the length of time they had experienced symptoms to invalidate differing opinions. A secondary theme, *responder usurping*, where participants attempted to redirect the groups’ focus from the original support-seeking post to themselves, was noted in our latent analysis of competitive excerpts.

Discussion/Conclusions: The results of this analysis suggest patients, in trying to prove the severity of their own suffering, further isolate themselves and potentially miss opportunities to experience emotional support, a sense of belonging, and to offer support for others. These results also suggest that even in a safe space, participants continue struggling to legitimize their suffering.

## Unravelling the relationship between parental PTSD and pediatric chronic pain: The mediating role of pain catastrophizing

Alexandra Neville and Melanie Noel

Psychology, University of Calgary, Calgary, Alberta, Canada

**CONTACT** Alexandra Neville alexandra.neville@ucalgary.ca

© 2017 Alexandra Neville and Melanie Noel. Published with license by Taylor & Francis.

This is an Open Access article distributed under the terms of the Creative Commons Attribution License (http://creativecommons.org/licenses/by/3.0/), which permits unrestricted use, distribution, and reproduction in any medium, provided the original work is properly cited. The moral rights of the named author(s) have been asserted.

Introduction/Aim: High rates of post-traumatic stress disorder (PTSD) symptoms have recently been found among parents of youth with chronic pain. Moreover, higher parental PTSD symptoms have been linked to worse child pain outcomes. Children’s and parents’ catastrophic thinking about child pain are robust predictors of child pain symptoms and have been proposed as potential mechanisms through which parental PTSD influences children’s pain. This study is the first to examine whether child and parent pain catastrophizing mediates the relationship between parental PTSD symptoms and child pain among a cohort of youth with chronic pain.

Methods: Eighty-nine children diagnosed with chronic pain (65% female, *M_age_* = 13.43, Range = 8–17 years) in a tertiary level chronic pain program and their parents (92.5% mothers) completed data collection. Parents completed self-report measures of PTSD symptoms and catastrophizing about child pain. Children completed self-report measures of pain catastrophizing and pain interference.

Results: Analyses using the Preacher and Hayes bootstrapping macro showed that the relationship between parental PTSD symptoms and child pain interference was mediated by higher levels of parent pain catastrophizing (95% CI Lower to Upper = .023–.234) and child pain catastrophizing (95% CI Lower to Upper = .074–.240). Parent and child pain catastrophizing accounted for 11% and 14% of the variance in the relationship between parental PTSD symptoms and child pain interference, respectively.

Discussion/Conclusions: Parent *and* child catastrophic thinking about pain play important roles in understanding how parental PTSD symptoms influence children’s chronic pain experience and may inform the refinement of family-based interventions to reduce children’s pain.

## Is sex worth the pain? Willingness to engage in sexual activity among partnered women with Fibromyalgia

Kirsten M. Gullickson^a^, Lyndsay Crump^a^, Diane L. LaChapelle^a^, Pablo Santos-Iglesias^b^, and E. Sandra Byers^a^

^a^Psychology, University of New Brunswick, Fredericton, New Brunswick, Canada; ^b^Oncology, University of Calgary, Calgary, Alberta, Canada

**CONTACT** Kirsten M. Gullickson kgullick@unb.ca

© 2017 Kirsten M. Gullickson, Lyndsay Crump, Diane L. LaChapelle, Pablo Santos-Iglesias, and E. Sandra Byers. Published with license by Taylor & Francis.

This is an Open Access article distributed under the terms of the Creative Commons Attribution License (http://creativecommons.org/licenses/by/3.0/), which permits unrestricted use, distribution, and reproduction in any medium, provided the original work is properly cited. The moral rights of the named author(s) have been asserted.

Aim: Fibromyalgia (FM) negatively impacts sexual functioning although little is known about the nature of its impact, therefore we qualitatively explored how and why FM impacts sexual well-being among women in committed relationships.

Methods: Seventeen women with FM, who reported being in a relationship for at least 12 months, provided demographic/health information then participated in a semi-structured interview. Participants were asked to describe the impact of FM on various aspects of their sexual functioning (e.g., frequency, desire, arousal, satisfaction). Interview audio recordings were transcribed verbatim and the content was coded using Thematic Analysis.

Results: The most commonly reported impacts of FM on sexual well-being were decreased sexual frequency and reduced sexual satisfaction. Participants identified numerous barriers to sex, including muscle pain/soreness, fatigue, depression, decreased arousal, and negative body image. The majority of participants described making few attempts to engage in sexual activity, but a minority prioritized their sexual relationship and described efforts to adjust their sexual scripts to accommodate FM (e.g., changing the timing, duration, location, or nature of their sexual encounters). Participants reported varying levels of emotional distress and relationship conflict as a result of the sexual impacts of FM.

Conclusions: In women with FM, willingness to work toward a positive sexual relationship and degree of associated emotional distress may depend on whether the individual and their partner consider sex to be a valued activity to be prioritized. Encouraging clarification of sexual and relationship values may facilitate chronic pain acceptance and aid individual and dyadic adjustment to FM.

## Chiropractors’ screening and management of psychosocial factors for patients with low back pain: A qualitative study

Peter Stilwell^a^, Piaf Des Rosiers^b^, Jill A. Hayden^c^, Katherine Harman^b^, Simon D. French^d^, Janet A. Curran^e^, and Warren Hefford^f^

^a^Health Professions, Dalhousie University, Halifax, Nova Scotia, Canada; ^b^Physiotherapy, Dalhousie University, Halifax, Nova Scotia, Canada; ^c^Community Health and Epidemiology, Dalhousie University, Halifax, Nova Scotia, Canada; ^d^School of Rehabilitation Therapy, Queens University, Kingston, Ontario, Canada; ^e^Nursing, Dalhousie University, Halifax, Nova Scotia, Canada; ^f^School of Health and Human Performance, Dalhousie University, Halifax, Nova Scotia, Canada

**CONTACT** Peter Stilwell peterstilwell@dal.ca

© 2017 Peter Stilwell, Piaf Des Rosiers, Jill A. Hayden, Katherine Harman, Simon D. French, Janet A. Curran, and Warren Hefford. Published with license by Taylor & Francis.

This is an Open Access article distributed under the terms of the Creative Commons Attribution License (http://creativecommons.org/licenses/by/3.0/), which permits unrestricted use, distribution, and reproduction in any medium, provided the original work is properly cited. The moral rights of the named author(s) have been asserted.

Introduction/Aim: Psychosocial factors are consistently linked to low back pain chronicity and poor outcomes. Low back pain guidelines recommend screening for psychosocial factors and appropriately managing them to improve patient outcomes. This study aimed to assess chiropractors’ awareness of low back pain clinical practice guidelines and to identify barriers and facilitators to the screening and management of psychosocial factors for patients with low back pain.

Methods: This qualitative study used semi-structured interviews informed by the Theoretical Domains Framework with Nova Scotian chiropractors.

Results: None of the 10 participants interviewed could name specific low back pain clinical practice guidelines. However, they correctly described what the guidelines generally recommend. We identified six themes related to barriers and facilitators for chiropractors screening and managing psychosocial factors. The themes revolved around the participants’ desire to fulfill patients’ anatomy-focused treatment expectations and a perceived lack of training on managing psychosocial factors. This was compounded by concerns about going beyond the chiropractic scope of practice and a perceived lack of practical psychosocial screening and management resources. Furthermore, social factors, such as the influence of other healthcare practitioners, were reported to act as barriers or facilitators to screening and managing psychosocial factors.

Discussion/Conclusions: Most chiropractors in our study treated with a biomedical approach and reported that they do not always address psychosocial factors identified in their patients with low back pain. Many of the barriers identified appeared to be modifiable with relatively low-cost interventions, such as continuing education using evidence-informed behaviour change techniques.

## Osteoarthritis pain and neuropathy in mice is caused by neutrophil elastase through activation of proteinase-activated receptor-2

Milind M. Muley^a^, Allison R. Reid^a^, and Jason J. McDougall^b^

^a^Department of Pharmacology, Dalhousie University, Halifax, Nova Scotia, Canada; ^b^Department of Pharmacology, Department of Anesthesia, Pain Management & Perioperative Medicine, Dalhousie University, Halifax, Nova Scotia, Canada

**CONTACT** Milind M. Muley milind.muley@dal.ca

© 2017 Milind M. Muley, Allison R. Reid, and Jason J. McDougall. Published with license by Taylor & Francis.

This is an Open Access article distributed under the terms of the Creative Commons Attribution License (http://creativecommons.org/licenses/by/3.0/), which permits unrestricted use, distribution, and reproduction in any medium, provided the original work is properly cited. The moral rights of the named author(s) have been asserted.

This is an Open Access article distributed under the terms of the Creative Commons Attribution License (http://creativecommons.org/licenses/by/3.0/), which permits unrestricted use, distribution, and reproduction in any medium, provided the original work is properly cited. The moral rights of the named author(s) have been asserted.

Introduction/Aim: The aim of this study was to examine the contribution of neutrophil elastase and proteinase-activated receptor-2 (PAR2) to the development of joint pain associated with monoiodoacetate (MIA)-induced osteoarthritis (OA).

Methods: Experimental OA was induced in male C57BL/6 mice (22-36g) by injecting MIA (0.3mg/10µl) into the right knee joint. One cohort of animals received a synthetic inhibitor of neutrophil elastase (Sivelestat: 50 mg/kg i.p.) 10 min before and 240 min after MIA injection, and once on days 1 to 3. A second cohort received an endogenous inhibitor of neutrophil elastase (alpha-1 antitrypsin: 10 µg i.p.) 15 min before and 12 hours after MIA injection. The role of PAR2 receptors was investigated by comparing the development of MIA-induced OA in wild-type and PAR2-knockout mice. Pain was measured using von Frey hair algesiometry at several time points over two weeks. Joint nerve damage was also assessed on day 14 by measurement of saphenous nerve fibre myelin thickness using G-ratio values.

Results: Joint pain appeared on day 1 and persisted to day 14 post-MIA (P<0.0001). Early blockade of neutrophil elastase using Sivelestat or alpha-1 antitrypsin produced significant improvement in joint pain throughout the time course (P<0.0001). PAR2 knock-out animals injected with MIA showed reduced joint pain (P<0.0001) over the two week time course and less saphenous nerve demyelination (P<0.05).

Discussion/Conclusions: Early inhibition of neutrophil elastase improves later pain responses in MIA-induced OA. Knock out of PAR2 prevents the development of MIA-induced tactile allodynia and demyelination, suggesting these effects are mediated by a PAR2 mechanism.

### Acknowledgements

Funding provided by CIHR, The Arthritis Society, Nova Scotia Health Research Foundation, Nova Scotia Graduate Innovation Fund.

## Outcome domains to evaluate paediatric chronic pain rehabiliation program success: What is meaningful to youth, parents, and other important stakeholders?

Karen Hurtubise^a^, Melanie Noel^b^, Lauren McCallum^c^, Astrid Brousselle^d^, and Chantal Camden^e^

^a^Faculty of Medicine and Health Sciences, School of Rehabilitation, University of Sherbrooke, Sherbrooke, Quebec, Canada; ^b^Department of Psychology, Faculty of Arts, University of Calgary, Calgary, Alberta, Canada; ^c^Vi Riddell Pain and Rehabilitation Centre, Alberta Health Services, Calgary, Alberta, Canada; ^d^Community Health, University of Sherbrooke (Longueil Campus), Longueil, Quebec, Canada; ^e^School of Rehabilitation, University of Sherbrooke, Sherbrooke, Quebec, Canada

**CONTACT** Karen Hurtubise karen.hurtubise@usherbrooke.ca

© 2017 Karen Hurtubise, Melanie Noel, Lauren McCallum, Astrid Brousselle, and Chantal Camden. Published with license by Taylor & Francis.

This is an Open Access article distributed under the terms of the Creative Commons Attribution License (http://creativecommons.org/licenses/by/3.0/), which permits unrestricted use, distribution, and reproduction in any medium, provided the original work is properly cited. The moral rights of the named author(s) have been asserted.

Introduction/Aim: Paediatric chronic pain rehabilitation programs for youth with high levels of pain-related disability require the collaboration of multiple stakeholders, thus creating challenges in establishing common outcomes upon which to evaluate these interventions. Previous outcome recommendations (PedIMMPACT) have not formally integrated the perspectives of youth with chronic pain, their parents, and other important stakeholders (e.g., teachers). This study aimed to identify outcome domains sensitive to the perspectives and needs of these various stakeholders.

Methods: An exploratory qualitative design guided this study. Data was gathered from a 12-member advisory group, composed of youth with chronic pain, their parents, clinicians, healthcare managers, and teachers, using a combination of electronic questionnaires and audio-recorded focus groups. A thematic content analysis process was used to analyse the data.

Results: All participants described the intervention goal as improving function despite the pain. Four primary outcome domains emerged: 1) independence in self-management of pain condition, daily activities, and health needs; 2) participation in meaningful activities with family, school, friends, and in the community; 3) quality of life of the youth and their family members, including sleep, mood and affect, and self-efficacy; and 4) costs to the family and the healthcare system. Pain intensity and frequency were seen as secondary outcome domains.

Discussion/Conclusions: Stakeholder–identified do-mains provide valuable information about the expectations of various stakeholders involved in the complex rehabilitation interventions for youth with pain-related disability and can create a foundation upon which effectiveness evaluation studies of these programs can be based.

## Siblings in pain: The relationship between siblings’ behaviours and children’s pain outcomes

Meghan Schinkel, Christine Chambers, Penny Corkum, and Sophie Jacques

Department of Psychology & Neuroscience, Dalhousie University, Halifax, NS, Canada

**CONTACT** Meghan Schinkel meghan.schinkel@dal.ca

© 2017 Meghan Schinkel, Christine Chambers, Penny Corkum, and Sophie Jacques. Published with license by Taylor & Francis.

This is an Open Access article distributed under the terms of the Creative Commons Attribution License (http://creativecommons.org/licenses/by/3.0/), which permits unrestricted use, distribution, and reproduction in any medium, provided the original work is properly cited. The moral rights of the named author(s) have been asserted.

Introduction/Aim: The study explored the relationship between siblings’ behaviours and children’s pain outcomes during cold-pressor pain.

Methods: 92 sibling dyads between the ages of 8–12 took turns participating in the cold pressor task (CPT) in a counterbalanced order with their sibling present. The behaviour of the observing sibling was coded as attending (e.g., symptom talk), non-attending (e.g., distraction) and coping/encouragement behaviour. Pain tolerance, as assessed by immersion duration, was recorded. Following the CPT, children rated their pain intensity and pain-related fear.

Results: A series of actor-partner interdependence models using structural equation modelling was conducted. The child who participated in the CPT first was considered Sibling 1, and the child who participated second was considered Sibling 2. Greater levels of coping behaviours by Sibling 2 was related to higher pain intensity (b* = .27, *p* < .01) and fear (b* = .17, *p* < .05) reported by their participating sibling. Further, greater coping behaviours by Sibling 1 was related to their own greater fear (b* = .22, *p* < .05), and greater levels of coping behaviours by Sibling 2 was related to their own greater pain intensity ratings (b* = .19, *p* < .05). Greater attending behaviours by Siblings 1 (b* = −.15, *p* < .05) and 2 (b* = −.26, *p* < .01) was related to lower pain tolerance for their participating siblings. Greater non-attending behaviours by Sibling 2 was related to higher fear ratings by their participating sibling (b* = .25, *p* < .05).

Discussion/Conclusions: The findings suggest that the behaviours of a present sibling related to children’s pain experiences, with behaviours that drew attention to the pain/experience generally being associated with poorer outcomes.

## Pulsed radiofrequency for the treatment of chronic post-herniorraphy inguinal pain

Y. Lemay Lachance^a^, Jean-François Canuel^b^, Maria Luz Padilla Del Rey^c^

^a^Department of Anesthesiology, CHU de Québec, Université Laval, Québec City, Québec, Canada; ^b^Department of Anesthesiology, CHU de Québec, Université Laval, Québec City, Québec, Canada; ^c^Department of Anesthesiology, Hospital General Universitario Morales, Murcia, Murcia, Spain

**CONTACT** Yves Lemay Lachance yveslachance@gmail.com

© 2017 Yves Lemay Lachance, Jean-François Canuel, and Maria Luz Padilla Del Rey. Published with license by Taylor & Francis.

This is an Open Access article distributed under the terms of the Creative Commons Attribution License (http://creativecommons.org/licenses/by/3.0/), which permits unrestricted use, distribution, and reproduction in any medium, provided the original work is properly cited. The moral rights of the named author(s) have been asserted.

Introduction/Aim: Inguinal hernia repair is one of the most common surgical procedures in the world. Chronic pain following herniorraphy is now recognized as the most significant complication of this procedure and is due to primary or secondary injuries to nerve tissue. Multiple pharmacologic, non-pharmacologic and surgical treatment modalities have been used, and have varied success rates. Pulsed radiofrequency (PRF) is fairly new in the treatment of chronic pain, and has been used successfully in the treatment of groin pain. It is safe, non-neurodestructive and is less painful than continuous radiofrequency. The purpose of our study is to examine a case series of three patients with chronic ilioinguinal neuropathy following herniorraphy that were treated with PRF at our institution.

Methods: Three patients diagnosed with chronic ilioinguinal neuropathy secondary to herniorraphy were treated with ultrasound-guided ilioinguinal PRF at 42°C between 4 to 10 minutes, with a 20G 100mm needle (10mm active tip). All patients treated with PRF have had initial positive responses to ilioinguinal nerve blocks. All techniques were executed by the same algologist (JFC).

Results: After the PRF, 2/3 patients reported almost complete pain relief for at least 1 month. Past 1 month, all patients experienced chronic pain, but at a decreased level compared to the period before the PRF. No complications were observed.

Discussion/Conclusions: In the three patients studied, PRF was successful in diminishing chronic ilioinguinal pain following herniorraphy without complications. Further studies are needed to confirm our results.

## Effectiveness of live zoster vaccine in preventing postherpetic neuralgia (PHN)

Morgan Marks^a^, Joan Bartlett^b^, Bruce Fireman^b^, John Hansen^b^, Edwin Lewis^b^, Laurie Aukes^b^, Yong Chen^a^, Nicola Klein^b^, Patricia Saddier^a^, and Roger Baxter^b^

^a^Pharmacoepidemiology Department, Merck & Co., Inc., Kenilworth, New Jersey, United States; ^b^Kaiser Permanente Vaccine Study Center Kaiser Permanente Northern California, Oakland, California, United States

**CONTACT** Morgan Marksmorgan.marks@merck.com

© 2017 Morgan Marks, Joan Bartlett, Bruce Fireman, John Hansen, Edwin Lewis, Yong Chen, Nicola Klein, Patricia Saddier, and Roger Baxter. Published with license by Taylor & Francis.

This is an Open Access article distributed under the terms of the Creative Commons Attribution License (http://creativecommons.org/licenses/by/3.0/), which permits unrestricted use, distribution, and reproduction in any medium, provided the original work is properly cited. The moral rights of the named author(s) have been asserted.

Introduction/Aim: A single dose, live attenuated zoster vaccine, is licensed in >50 countries for the prevention of herpes zoster and PHN. Duration of protection is evaluated in a long-term observational study. We previously reported that vaccine effectiveness (VE) to prevent zoster tended to decline over time and was on average 45–50% over 5 years in people ≥60 (60+) years. We present here the results of VE against PHN.

Methods: The study is conducted in a US healthcare plan as an open cohort that members enter unvaccinated when they become age-eligible for vaccination. PHN cases among vaccinated and unvaccinated zoster cases were identified as having a PHN-specific diagnosis code ≥90 days after first zoster code. VE against PHN was estimated using Cox regression adjusting for sex, birth year, race/ethnicity, healthcare use, comorbidities and immunocompromise status.

Results: In 2007–2014, ~400,000 subjects were vaccinated (coverage >50% in 60+) and ~50,000 zoster episodes with >3000 PHN cases occurred. VE against PHN was 82% (95% CI 76–87) in the first year, decreased in the second year, and then remained relatively stable through year 5, with an average VE over the first 5 years following vaccination of 72%, 69% and 61% in people vaccinated at age 60–69, 70–79, and 80+ years, respectively.

Discussion/Conclusions: Overall VE against PHN was ~70% in all 60+ age groups. VE against zoster and PHN in people 80+ was similar to younger 60+ groups, supporting vaccination of all eligible people, including the elderly who are at increased risk of zoster and PHN.

## A qualitative analysis of low back pain patient interviews in a tertiary care center emergency department

Alexander Stathakis^a^, Andrea Smith^b^, Rachel Ogilvie^b^, and Jill Hayden^b^

^a^Faculty of Medicine, Dalhousie University, Halifax, Nova Scotia, Canada; ^b^Community Health and Epidemiology, Dalhousie University, Halifax, Nova Scotia, Canada

**CONTACT** Alexander Stathakis a.stathakis@dal.ca

© 2017 Alexander Stathakis, Andrea Smith, Rachel Ogilvie, and Jill Hayden. Published with license by Taylor & Francis.

This is an Open Access article distributed under the terms of the Creative Commons Attribution License (http://creativecommons.org/licenses/by/3.0/), which permits unrestricted use, distribution, and reproduction in any medium, provided the original work is properly cited. The moral rights of the named author(s) have been asserted.

Introduction/Aim: Low back pain is a leading cause of disability globally. The majority seek medical care from their family physician, however, some visit emergency departments (ED). Our research aims to confirm why patients choose to seek emergency care for their low back pain, and what expectations of care they hold.

Methods: We interviewed 13 low back pain patients in our local ED before and after seeing a physician. Transcripts were analyzed using a theoretical framework (“a typology of reasons”) developed based on existing qualitative literature on the topic. Thematic results are presented.

Results: We confirmed many themes motivating patients’ decisions to visit the ED that have been suggested from previous literature, including intensity/change in pain, and access to diagnostics tools. Counter to previous research, patients did not report issues accessing their family physician, and overall expressed satisfaction with their family physician. While many voiced their reluctance to present to the ED, they also conveyed satisfaction with the care they received.

Discussion/Conclusions: Patients’ motivations were highly varied, though many fell within the typology of reasons developed through our comprehensive search of the literature. Furthermore, most patients did not receive any specialized interventions, such as diagnostic imaging, even if that had motivated them to present to the ED. Despite this, the majority of patients reported being satisfied with the care they received and felt they had made the right decision in visiting the ED for their low back pain.

## Pain acceptance mediates the relationship between pain intensity and pain-related disability in patients with chronic pain after cardiac surgery

Muhammad Abid Azam^a,^^b^, Aliza Weinrib^a,^^b^, Janice Montbriand^a,^^b^, Lindsay C. Burns^b^, Claire Wicks^b^, Hance Clarke^a,^^b^, and Joel Katz^a,^^b^

^a^Department of Anesthesia & Pain Management, Toronto General Hospital, Toronto, Ontario, Canada; ^b^Department of Psychology, York University, Toronto, Ontario, Canada

**CONTACT** Muhammad Abid Azam abidazam@yorku.ca

© 2017 Muhammad Abid Azam, Aliza Weinrib, Janice Montbriand, Lindsay C. Wall, Claire Wicks, Hance Clarke, and Joel Katz. Published with license by Taylor & Francis.

This is an Open Access article distributed under the terms of the Creative Commons Attribution License (http://creativecommons.org/licenses/by/3.0/), which permits unrestricted use, distribution, and reproduction in any medium, provided the original work is properly cited. The moral rights of the named author(s) have been asserted.

Introduction/Aim: Chronic post-surgical pain (CPSP) leads to significant disability. Research is needed on psychological processes that support people with CPSP in improving pain-related disability. One proposed process is pain acceptance, characterized as the experience of ongoing pain without attempts to avoid, reduce, or control it. For people with CPSP, pain acceptance may reduce disability by fostering greater re-engagement with valued life activities. The purpose of this study was to test the mediating role of pain acceptance in the relationship between pain intensity and disability in patients who had undergone coronary artery bypass graft surgery (CABGS) at least 6 months earlier.

Methods: N **=** 133 patients (Age_M_ = 62.67 years, SD = 11.74; 72% Male) reported CPSP. Pain intensity was measured using a 0-10 scale. Disability was measured using the Pain Disability Index. Pain acceptance was measured using the Chronic Pain Acceptance Questionnaire. Mediation with pain acceptance (mediator), pain intensity (predictor), and disability (outcome) was tested according to Hayes (2016) specifications.

Results: Pain intensity was positively correlated with disability (*r* = .44, *p <* .001) and inversely correlated with pain acceptance (*r* = -.45, *p* < .001). The mediation model revealed pain acceptance was a significant inverse predictor of disability (β = -.73, *p* < .001), while the direct effect of pain intensity on disability was not significant (β = .11, *p* = .22), supporting a full mediation.

Discussion/Conclusions: Results demonstrate that pain acceptance mediates the relationship between pain and disability in CPSP patients. Acceptance-based chronic pain interventions may assist sufferers in engaging in flexible and persistent patterns of valued activities.

## Quantitative sensory testing indicates differential pain thresholds and pain after-effects in patients with chronic pain after cardiac surgery

M. Abid Azam^a,^^b^, Aliza Weinrib^a,^^b^, Janice Montbriand^a,^^b^, Lindsay C. Wall Burns^b^, Claire Wicks^b^, Joel Katz^a,^^b^, and Hance Clarke^a,^^b^

^a^Department of Anesthesia & Pain Management, Toronto General Hospital, Toronto, Ontario, Canada; ^b^Department of Psychology, York University, Toronto, Ontario, Canada

**CONTACT** Muhammad Abid Azam abidazam@yorku.ca

© 2017 Muhammad Abid Azam, Aliza Weinrib, Janice Montbriand, Lindsay C. Wall, Claire Wicks, Joel Katz, and Hance Clarke. Published with license by Taylor & Francis.

This is an Open Access article distributed under the terms of the Creative Commons Attribution License (http://creativecommons.org/licenses/by/3.0/), which permits unrestricted use, distribution, and reproduction in any medium, provided the original work is properly cited. The moral rights of the named author(s) have been asserted.

Introduction/Aim: Chronic pain occurs in ~10% of patients following cardiac surgery. Little is known about sensory processing changes related to chronic post-surgical pain (CPSP). Quantitative sensory testing (QST) is comprised of methods to assess differential responses to noxious/non-noxious stimulation. This cross-sectional study examined QST measures in patients who had undergone coronary artery bypass graft surgery (CABGS) at least 6 months earlier.

Methods: 367 patients (Age_M_ = 66.05 years, SD = 11.58; 75% Male) underwent CPSP assessment and were grouped for presence of chronic pain at CABGS sites (CPSP; n = 133), other chronic pain (OCP; n = 97), or no pain (NP; n = 137). Pain detection thermal (cold/heat pain) thresholds (TPT) were measured at the chest and right forearm. Cold pressor task (CPT) was conducted with one arm submerged in cold water (M = 1.47°C, SD = .77) until pain was intolerable. Pain intensity and unpleasantness ratings were obtained using a 0-10 numeric rating scale 30 seconds after CPT arm withdrawal. One-way ANOVAs were conducted followed by Bonferroni post-hoc comparisons (α<.017).

Results: Group differences were found for heat (F_(2, 197)_ = 6.81, p = .001) and cold (F_(2, 197)_ = 3.19, p < .05) TPT-forearm, and CPT pain intensity (F_(2, 199)_ = 6.42, p < .005) and unpleasantness (F_(2, 199)_ = 5.60, p < .005). CPSP group had lower heat TPT-forearm pain thresholds compared to OCP (p = .016) and NP (p = .001) groups, and lower cold TPT-forearm thresholds compared to NP group (p = .01). CPSP group had higher CPT pain intensity (p < .001) and unpleasantness (p = .001) compared to NP group.

Discussion/Conclusions: Results suggest CPSP alters pain-modulatory processing leading to lower heat and higher cold pain thresholds, and prolonged cold pain effects after CPT arm withdrawal.

## Allosteric modulation of cannabinoid receptor 1 reduces ocular pain

Dinesh Thapa^a^, J. Thomas Toguri^a^, Elizabeth A. Cairns^a^, Ganesh A. Thakur^d^, and Melanie E.M. Kelly^a,^^b,^^c^

^a^Departments of Pharmacology, Anesthesia, Pain Management & Perioperative Medicine, and; ^b^Ophthalmology & Visual Sciences; ^c^Dalhousie University, Halifax, NS; ^d^Department of Pharmaceutical Sciences, Northeastern University, Boston, MA, United States.

**CONTACT** Dinesh ThapaDinesh.Thapa@Dal.Ca

© 2017 Dinesh Thapa, J. Thomas Toguri, Elizabeth A. Cairns, Ganesh A. Thakur, Melanie E.M. Kelly. Published with license by Taylor & Francis.

This is an Open Access article distributed under the terms of the Creative Commons Attribution License (http://creativecommons.org/licenses/by/3.0/), which permits unrestricted use, distribution, and reproduction in any medium, provided the original work is properly cited. The moral rights of the named author(s) have been asserted.

Aim: Current treatments for corneal pain are frequently ineffective. Cannabinoid receptor 1 (CB1R) orthosteric activation has antinociceptive effects, however, therapeutic applications are limited due to behavioral side-effects. Allosteric modulators bind to distinct sites at CB1R, and may offer an alternative approach to reduce pain with reduced side-effects. Therefore, the purpose of this research was to investigate the antinociceptive properties of the CB1R allosteric modulators GAT229 and GAT228 in a mouse model of corneal injury.

Methods: Corneal hyperalgesia was generated using chemical cauterization of the cornea in wildtype (WT) and CB2R knockout (CB2R-/-) mice. Cauterized eyes were treated topically with GAT229 and GAT228 (0.5-2%, w/v) alone or in combination with orthosteric CB1R agonist, ∆^8^- tetrahydrocannabinol (∆^8^THC), in the presence/absence of the CB1R antagonist AM251 (2mg/kg, ip). Increased number of eye blinks, squints and wipes, recorded 6 hours post-injury using capsaicin-stimulation were collectively termed as corneal hyperalgesia.

Results: GAT228 (1 & 2%), but not GAT229 (0.5 & 1%), reduced corneal hyperalgesia. The combination of 0.5% GAT229 with subthreshold ∆^8^THC (0.4%) significantly reduced hyperalgesia. The antinociceptive effects of both GAT229 and GAT228 were blocked by AM251, but remained unaffected in CB2R-/- mice.

Conclusion: The CB_1_R allosteric modulators GAT 229 and GAT 228 reduce corneal hyperalgesia either through potentiating the orthosteric activity or independently through allosteric activation. Allosteric modulation of CB1R could offer a novel approach for treating corneal pain while reducing the side-effects produced from CB1R orthosteric activation.

## Overview of reviews: relevant treatment modalities for management of low back pain in the emergency department

Bernard Burgesson^a^, Jill Hayden^b^, and Kirk Magee^c^

^a^Dalhousie Medical School, Dalhousie University, Halifax, Nova Scotia, Canada; ^b^Department of Community Health & Epidemiology, Dalhousie University, Halifax, Nova Scotia, Canada; ^c^Department of Emergency Medicine, Dalhousie University, Charles V. Keating Emergency & Trauma Centre, Halifax, Nova Scotia, Canada

**CONTACT** Bernard Burgesson Bernard.burgesson@dal.ca

© 2017 Bernard Burgesson, Jill Hayden, and Kirk Magee. Published with license by Taylor & Francis.

This is an Open Access article distributed under the terms of the Creative Commons Attribution License (http://creativecommons.org/licenses/by/3.0/), which permits unrestricted use, distribution, and reproduction in any medium, provided the original work is properly cited. The moral rights of the named author(s) have been asserted.

Introduction/Aim: Low back pain remains a condition with relatively high incidence and prevalence, affecting 70–85% of people at some point in their lives. Low back pain management may be best suited for primary care, yet it is one of the most common reasons for presentation to emergency departments.

Methods: The objective of this study was to provide a summary of the evidence on effectiveness of treatments relevant to the emergency department for adults with low back pain. We conducted an overview of systematic reviews following robust methods advocated by Cochrane. We searched the Cochrane Library (including Cochrane Database of Systematic Reviews and Database of Abstracts of Reviews), and included systematic reviews of randomized controlled trials for treatments for low back pain appropriate for delivery in the emergency department setting.

Results: Salicylate-containing rubefacients and superficial heat provide a statistically significant reduction in pain compared to placebo for acute low back pain. For chronic low back pain, buprenorphine (opioid) provided both a statistically significant and clinically important improvement in pain. We will provide results on other key outcome measures: function/disability and adverse events.

Discussion/Conclusions: Majority of the interventions included in this study provided a small but statistically significant reduction in pain for both acute and chronic low back pain. Buprenorphine (opioid) produced a clinically important improvement in pain for chronic low back pain. Our study is the first overview of reviews to summarize the evidence on the effectiveness of treatments in emergency department settings for reducing pain and disability in patients with low back pain.

## It Doesn’t Have to Hurt: An analysis of earned media coverage and public engagement

Tracy Moniz^a^, Christine Chambers^b^, Erica Ehm^c^, and Justine Dol^d^

^a^Department of Communication Studies, Mount Saint Vincent University; ^b^Canada Research Chair in Children’s Pain (Tier 1), Professor of Pediatrics and Psychology & Neuroscience, Dalhousie University and IWK Health Centre; ^c^Ehm & Co, Founder, YummyMummyClub.ca; ^d^Department of Health Professions, Dalhousie University, Centre for Pediatric Pain Research, IWK Health Centre

**CONTACT** Tracy Moniz Tracy.Moniz@msvu.ca Mount Saint Vincent University, 166 Bedford Hwy., Halifax, Nova Scotia, B3M 2J6, Canada.

© 2017 Tracy Moniz, Christine Chambers, Erica Ehm, Justine Dol. Published with license by Taylor & Francis.

This is an Open Access article distributed under the terms of the Creative Commons Attribution License (http://creativecommons.org/licenses/by/3.0/), which permits unrestricted use, distribution, and reproduction in any medium, provided the original work is properly cited. The moral rights of the named author(s) have been asserted.

Aims: “It Doesn’t Have to Hurt” (IDHTH) is a social media campaign that ran for one year (September 2015-16) to increase parental awareness and use of evidence-based knowledge about children’s pain. This study documents and analyzes earned media coverage (traditional and online) of IDHTH, along with the public engagement it generated.

Methods: This study involved quantifying and qualifying via content analysis earned media coverage of the IDHTH campaign, drawing on the methods of Kornfield et al. (2015) for measuring and analyzing earned media and ensuing public engagement.

Results: The search yielded 61 pieces mentioning the IDHTH campaign, of which all but one focused specifically on the campaign. Online media comprised 73.7% of coverage and traditional media accounted for 26%. Of total coverage, 55.7% of pieces included campaign-produced multimedia, most notably hyperlinks (37%), videos (31%) and social media images (18%). Fifty-nine per cent of coverage generated social media engagement (4,523 social media ‘hits’), notably general shares where the platform was not specified (67.8%) and Facebook shares (11.5%) and likes (10.1%). The online pieces where audience commentary was enabled generated 138 comments. Earned media coverage and audience commentary are being analyzed for content themes.

Conclusions: The findings indicate that earned media extended the reach and impact of the IDHTH campaign and offers a window into audience reception of and engagement with public communication about children’s pain. This study serves as one measure of the effectiveness of a science-media partnership in promoting knowledge-translation efforts to bridge the critical knowledge-to-action gap in children’s pain.

## Acute and prophylactic treatment with cannabidiol attenuates pain in a rat model of osteoarthritis

H. T. Philpott, and J. J. McDougall

Departments of Pharmacology and Anesthesia, Pain Management & Perioperative Medicine, Dalhousie University, Halifax, NS, Canada

**CONTACT** Holly T. Philpott holly.philpott@dal.ca

© 2017 Holly T. Philpott and Jason J. McDougall. Published with license by Taylor & Francis.

This is an Open Access article distributed under the terms of the Creative Commons Attribution License (http://creativecommons.org/licenses/by/3.0/), which permits unrestricted use, distribution, and reproduction in any medium, provided the original work is properly cited. The moral rights of the named author(s) have been asserted.

Introduction/Aim: Cannabidiol (CBD), a non-psychoactive component of the Cannabis plant, is believed to have therapeutic potential for the treatment of pain. The aim of the present study was to determine whether CBD could decrease pain in a chronic pre-clinical model of osteoarthritis (OA). The effect of early treatment with CBD on OA pain development was also investigated.

Methods: OA was induced in male Wistar rats (232–309g) by intra-articular (i.artic.) injection of sodium monoiodoacetate (MIA 3mg/50µl). On day 14 after OA induction, joint pain was measured by hindlimb weight bearing and hindpaw tactile allodynia. CBD (300µg/50µl, n = 8) or vehicle (50µl DMSO:cremophor:0.9% NaCl, 1:1:8, n = 8) was locally administered (i.artic.) and joint pain was assessed over 4 hours. Prophylactic use of CBD was assessed in a separate cohort of rats, where CBD (300µg/50µl, n = 8) or vehicle (50µl, n = 8) was administered subcutaneously (s.c.) over the ipsilateral knee joint 30 min prior to the induction of OA and every 24 hours for the first three days thereafter. Joint pain was then measured over the course of MIA development on days 0, 1, 2, 3, 7, 10, and 14.

Results: Acute administration of CBD on day 14 significantly improved hindpaw withdrawal threshold (p < 0.0001) and hindlimb weight bearing (p = 0.0013). Prophylactic administration of CBD attenuated hindpaw withdrawal threshold (p < 0.0001) over the course of 14 days, but did not significantly improve hindlimb weight bearing (p > 0.05).

Discussion/Conclusions: This study suggests for the first time that *local* CBD administration attenuates pain in established OA, and early prophylactic CBD treatment prevented the later development of pain in OA.

## A preliminary evaluation of an interdisciplinary group promoting activity pacing in indiviuals with chronic pain

Douglas Cane, Julie Sheppard, and Lindsay Edwards

Pain Management Unit, Nova Scotia Health Authority, Halifax, Nova Scotia, Canada

**CONTACT** Douglas Cane douglas.cane@nshealth.ca

© 2017 Douglas Cane, Julie Sheppard, and Lindsay Edwards. Published with license by Taylor & Francis Published by Taylor & Francis

This is an Open Access article distributed under the terms of the Creative Commons Attribution License (http://creativecommons.org/licenses/by/3.0/), which permits unrestricted use, distribution, and reproduction in any medium, provided the original work is properly cited. The moral rights of the named author(s) have been asserted.

Introduction/Aim: Instruction in activity pacing is frequently included in the treatment of individuals with chronic pain. Research suggests, however, that individuals with chronic pain experience difficulty employing this strategy and that several factors limit their ability to do so. This study examined the effectiveness of an interdisciplinary group program addressing these issues, and examined the relationship between changes in beliefs about pacing and changes in psychosocial functioning.

Methods: Fifteen individuals with chronic pain with ongoing difficulties employing activity pacing completed a five-week interdisciplinary group program. The group incorporated discussion and homework to provide opportunities to challenge beliefs that limit activity pacing (e.g., concerns about maintaining productivity, completing activities once started, and concerns about the responses of others). At the beginning and end of group, individuals completed the Pacing Obstacles Questionnaire (POQ) which measures beliefs about pacing, and measures of affect, activity, pain acceptance, and perceived disability. Correlations between changes in beliefs and changes in the outcome measures were examined to explore the relationship between beliefs and functioning, and to evaluate the effectiveness of treatment.

Results: A significant decrease in perceived obstacles to activity pacing was observed. Decreased concerns about pacing were related to increased use of pacing, improved affect, and greater acceptance of pain.

Discussion/Conclusions: Participation in an interdisciplinary group program designed to address issues and concerns about activity pacing successfully increased acceptance and use of this strategy to manage pain. Greater willingness to use activity pacing was also associated with increased use of pacing, improved affect, and increased acceptance of pain.

## Past month prevalence of chewing, snorting, smoking and injecting prescription opioid tablets and capsules among individuals entering substance abuse treatment

Stevan Geoffrey Severtson^a^, Scott Kreider^a^, Becki Bucher Bartelson^a^, Matthew Ellis^b^, Colleen M. Haynes^a^, Theodore Cicero^b^, Jody L. Green^a,^^c^, and Richard C. Dart^a,^^c^

^a^Rocky Mountain Poison and Drug Center, Denver Health and Hospital Authority, Denver CO, United States; ^b^Washington University, St Louis MO, United States; ^c^Canadian Consumer Product and Pharmaceutical Safety Inc., Toronto, Ontario

**CONTACT** Colleen M. Haynes Colleen.Haynes@rmpdc.org

© 2017 Stevan Geoffrey Severtson, Scott Kreider, Becki Bucher Bartelson, Matthew Ellis, Colleen M. Haynes, Theodore Cicero, Jody L. Green, and Richard C. Dart. Published with license by Taylor & Francis.

This is an Open Access article distributed under the terms of the Creative Commons Attribution License (http://creativecommons.org/licenses/by/3.0/), which permits unrestricted use, distribution, and reproduction in any medium, provided the original work is properly cited. The moral rights of the named author(s) have been asserted.

Aims: Assess past month prevalence for routes of abuse in commonly prescribed opioid analgesics among individuals entering treatment for substance abuse.

Methods: The Survey of Key Informants’ Patients Program is comprised of 30 Key Informants in Ontario (clinicians, epidemiologists, treatment centre counselors, and others in positions to report on drug problems). Patients of Key Informants complete an anonymous questionnaire on prescription and illicit drug use, including source of acquisition, route of administration and basic demographic information upon admission.

Results: From January 2016 through September 2016, 302 respondents completed the questionnaire; 212 (70.2%) endorsed past month abuse of a prescription opioid tablet/capsule, and 185 (87.3%) of these chewed, snorted, smoked or injected. The most endorsed products were oxycodone (n = 137, 45.4% of respondents), hydromorphone (n = 107, 35.4%), codeine (n = 64, 21.2%), and morphine (n = 51, 16.9%). Among oxycodone abusers, the route of abuse was chewing (n = 67, 48.9%), snorting (n = 39, 28.5%), smoking (n = 4, 2.9%) and injection (n = 20, 14.6%). For hydromorphone, the route of abuse was chewing (n = 21, 19.6%), snorting (n = 32, 29.9%), smoking (n = 2, 1.9%) and injection (n = 56, 52.3%). For codeine, the route of abuse was chewing (n = 49, 76.6%), snorting (n = 7, 10.9%), smoking (n = 1, 1.6%) and injection (n = 1, 1.6%). For morphine, the route of abuse was chewing (n = 13, 25.5%), snorting (n = 12, 23.5%), smoking (n = 0, 0%) and injection (n = 28, 54.9%).

Conclusions: Most respondents endorsed chewing, snorting, smoking or injecting a prescription opioid tablet/capsule in the month prior to entering treatment. The most endorsed route of administration varied within each active pharmaceutical ingredient.

## What institutional resources are available to support registered nurses in delivering pediatric pain care in rural northern Ontario?

Carolyn Truskoski 0000-0003-3925-9418 ^a^, Paula Forgeron 0000-0002-4686-9698 ^a^, Denise Harrison 0000-0001-7549-7742^a^, Young Nancy L. 0000-0002-1739-3299^b^

^a^Faculty of Health Sciences, School of Nursing, University of Ottawa, Ottawa, Ontario, Canada; ^b^Faculty of Health Sciences, School of Rural and Northern Health, Laurentian University, Sudbury, Ontario, Canada

**CONTACT** Carolyn Truskoski ctrus063@uottawa.ca

© 2017 Carolyn Truskoski, Paula Forgeron, Denise Harrison, and Nancy L. Young. Published with license by Taylor & Francis.

This is an Open Access article distributed under the terms of the Creative Commons Attribution License (http://creativecommons.org/licenses/by/3.0/), which permits unrestricted use, distribution, and reproduction in any medium, provided the original work is properly cited. The moral rights of the named author(s) have been asserted.

Introduction/Aim: To identify the availability of institutional resources (e.g. policies, continuing education) to support nurses’ pediatric pain management practices in rural northern Ontario hospitals.

Methods: An exploratory descriptive online survey was conducted using a pilot tested study specific questionnaire. A purposeful sampling method was used to recruit nurse administrators from eligible northern rural hospitals with dedicated inpatient pediatric beds. Descriptive statistics were used to quantify the types of institutional resources.

Results: Representatives from eight of nine eligible sites responded to the survey. On average hospitals admitted 50 (SD = 48) pediatric patients per year and had an average of 3392 (SD = 3625) pediatric visits (i.e. emergency and outpatient clinics) per year. Most respondents reported that nurses used pain assessment tools, but not all sites had a range of tools to meet the developmental differences across the pediatric population. Most sites (n = 6, 75%) had several policies or procedures to support pediatric pain care. Sucrose and kangaroo care were not commonly used for infants, despite their effectiveness, low cost and ease of use, yet all sites identified consistent use of topical anesthetic for needle pokes. Not all sites offered introductory or continuing education on pediatric pain. Self-identified scarcity of resources (i.e. no dedicated funding for education) was identified as a challenge. Guidelines or order sets were suggested as potential strategies to improve pain care in the rural context.

Discussion/Conclusions: The findings from this study provide insights into how best to improve tailored strategies to improve pediatric pain care in the rural Northern Ontario.

## Cancer treatment-related pain is inadequately managed 1 year after breast cancer surgery

Lynn R. Gauthier^a^, Vanja Cabric^b^, Rebecca Harrison^b^, A. M. Easson^c^, Vincent Chan^d^, Madeline Li^e^, and Lucia Gagliese^e,^^f^

^a^Département de médecine familiale et de médecine d’urgence, Université Laval, Québec, QC, Canada; ^b^Michael G. DeGroote School of Medicine, McMaster University, Hamilton, ON, Canada; ^c^Department of Surgical Oncology, University Health Network, Toronto, ON, Canada; ^d^Department of Anesthesia and Pain Management, University Health Network, Toronto ON, Canada; ^e^Department of Supportive Care, University Health Network, Toronto, ON, Canada; ^f^School of Kinesiology and Health Science, York University, Toronto, ON, Canada

**CONTACT** Lucia Gagliese gagliese@yorku.ca

© 2017 Lynn R. Gauthier, Vanja Cabric, Rebecca Harrison, Alexandra M. Easson, Vincent Chan, Madeline Li, and Lucia Gagliese. Published with license by Taylor & Francis.

This is an Open Access article distributed under the terms of the Creative Commons Attribution License (http://creativecommons.org/licenses/by/3.0/), which permits unrestricted use, distribution, and reproduction in any medium, provided the original work is properly cited. The moral rights of the named author(s) have been asserted.

Introduction/Aim: 20–60% of women experience chronic postoperative pain after breast cancer surgery (BCS). They may also undergo several other potentially painful treatments in the year following BCS. It is unclear whether they receive adequate pain management and the factors associated with analgesic adequacy are unknown. This study aimed to describe analgesic adequacy in women reporting cancer treatment-related pain (CTP) 1 year after BCS and identify its correlates.

Methods: 161 women who underwent unilateral or bilateral lumpectomy or mastectomy completed measures of pain and physical and psychosocial wellbeing 1 year after BCS. Sociodemographic and clinical data were collected. Analgesic adequacy was measured with the Pain Management Index (PMI). Descriptive statistics characterized women reporting CTP daily or most days of the week. Backwards linear regression identified correlates of CTP analgesic adequacy.

Results: 66 (41%) women reported CTP 1 year after BCS. They were 52.3±10.7 years old. Average pain was mild (2.5 ± 2.2) but 12% reported moderate-to-severe pain (≥5/10). The majority (n = 39; 59%) had inadequate analgesia (PMI <0). Of these, most (76.7%) were not prescribed any analgesics and 23.3% were prescribed an NSAID or adjuvant analgesic. Correlates of adequate analgesia were older age (B = .22, p = .09), lower pain intensity (B = -.63, p = .001), higher pain interference (B = .40, p = .05), and lower pain-related stoicism (B = −.29, p = .04) and pain catastrophizing (B = −.38, p = .02).

Discussion/Conclusions: Most women with CTP 1 year after BCS received inadequate analgesia. Inadequate analgesia was associated with pain, impact, and psychological factors, highlighting the multifactorial nature of analgesic adequacy. Future research is needed to further elucidate CTP management.

## Preschool children’s coping responses in the vaccination context: Caregiver and child predictors from infancy and preschool

Lauren Campbell^a^, R. Pillai Riddell^a^, Robert Cribbie^a^, Hartley Garfield^b^, and Saul Greenberg^b^

^a^Psychology, York University, Toronto, ON, Canada; ^b^Pediatrics, University of Toronto, Toronto, ON, Canada

**CONTACT** Lauren Campbell Lc15@yorku.ca

© 2017 Lauren Campbell, Rebecca Pillai Riddell, Robert Cribbie, Hartley Garfield, and Saul Greenberg. Published with license by Taylor & Francis.

This is an Open Access article distributed under the terms of the Creative Commons Attribution License (http://creativecommons.org/licenses/by/3.0/), which permits unrestricted use, distribution, and reproduction in any medium, provided the original work is properly cited. The moral rights of the named author(s) have been asserted.

Introduction/Aim: To model the prediction of preschooler coping responses during vaccinations. Both caregiver and child variables from the 12-month and preschool vaccinations were included.

Methods: The data is part of an ongoing longitudinal study that followed caregivers and children from infancy to preschool. Two separate path models (Minute 1 and Minute 2 post-needle) were estimated using data from the 12-month vaccination (n = 548), the preschool vaccination (n = 302), and a preschool psychological assessment (n = 172). Predictors of a preschooler coping response composite were examined as well as the interrelationships between predictors.

Results: Caregiver sensitivity and verbal reassurance from the 12-month vaccination predicted children’s language abilities at preschool (B = .18, p = .024; B = .19, p = .020, respectively). Child language ability predicted child coping responses during Minute 1 (B = .23, p = .015). Caregiver proximal soothing at the 12-month vaccination predicted more optimal child executive functioning at preschool (B = −.24, p = .002). Infant regulatory capacity at the 12-month vaccination predicted caregiver worry pre-needle at the preschool vaccination (B = .15, p = .016). Caregiver worry pre-needle at the preschool vaccination predicted preschooler coping responses at Minute 2 (B = −.15, p = .016).

Discussion/Conclusions: For the first time in the literature, longitudinal dynamic infant-caregiver pathways predicting preschooler coping responses have been elucidated. Caregiver behaviors during vaccinations are not only critical to both child pain coping responses in the short and long-term but also relate to child cognitive abilities outside the vaccination context.

## Poor internal consistency and item redundancy on the modified beahviour pain scale and face, legs, activity, cry, and consolability scale

Miranda G. DiLorenzo, David B. Flora, and Rebecca Pillai Riddell

Psychology, York University, Toronto, ON, Canada

**CONTACT** Miranda G. DiLorenzo mgdilo@yorku.ca

© 2017 Miranda G. DiLorenzo, David B. Flora, and Rebecca Pillai Riddell. Published with license by Taylor & Francis.

This is an Open Access article distributed under the terms of the Creative Commons Attribution License (http://creativecommons.org/licenses/by/3.0/), which permits unrestricted use, distribution, and reproduction in any medium, provided the original work is properly cited. The moral rights of the named author(s) have been asserted.

Introduction/Aim: A major barrier to providing adequate pain relief to infants and young children is accurately measuring how much pain they are feeling. A number of observational pain instruments have been developed to provide objective measures of pain. However, to move the field further, construct validation and refinement of these tools is warranted. The objective of this study was to evaluate basic psychometric properties of two behavioral pain scales, MBPS (Taddio et al., 1995) and FLACC (Merkel et al., 1997), used to assess acute pain in infants and young children.

Methods: Caregivers and their children were videotaped during routine immunization appointments. The videotapes were coded for pain-related distress behaviours. Polychoric correlations between pain behaviours were examined immediately after the needle and 1-minute post-needle at 2-month (MBPS; n = 499), 12-month (MBPS; n = 548) and preschool immunizations (FLACC; n = 302).

Results: Very weak inter-item correlations were revealed on MBPS (e.g. face and movement at 2-months immediately after needle; *r* = .12), and FLACC (e.g. cry and activity at preschool immediately after needle, *r* = .04). Very high correlations were also found between items on both scales (e.g. cry and consolability on FLACC immediately after needle, *r* = .96).

Discussion/Conclusions: Weakly correlated items suggest poor internal consistency on both FLACC and MBPS. Very high correlations suggest redundancy that will bias internal consistency estimates. Given strong content validity of these measures, redefinition of core items appears warranted.

## A survey of canadian physicians on the knowledge of fibromyalgia diagnostic criteria and adherence to guidelines

Dinesh Kumbhare^a^, Sara Ahmad^b^, Tori Sander^c^, John Flannery^a^, Liza Grosman-Rimon^a^, and John Srbely^c^

^a^Department of Medicine, Division of Physical Medicine and Rehabilitation, Toronto Rehabilitation Institute, Toronto, Ontario, Canada; ^b^Department of Psychology, McMaster University, Hamilton, Ontario, Canada; ^c^Human Health and Nutritional Science, University of Guelph, Guelph, Ontario, Canada

**CONTACT** Dinesh Kumbhare dinesh.kumbhare@uhn.ca

© 2017 Dinesh Kumbhare, Sara Ahmad, Tori Sander, John Flannery, Liza Grosman-Rimon, and John Srbely. Published with license by Taylor & Francis.

This is an Open Access article distributed under the terms of the Creative Commons Attribution License (http://creativecommons.org/licenses/by/3.0/), which permits unrestricted use, distribution, and reproduction in any medium, provided the original work is properly cited. The moral rights of the named author(s) have been asserted.

Introduction/Aim: In 2010, Wolfe et al demonstrated poor physician use of the 1990 fibromyalgia diagnostic criteria, and amended the criteria to address physician shortcomings. It is unknown whether physicians are using these criteria. The objective of this study was to investigate physician knowledge and use of the FM diagnostic criteria and treatment modalities.

Methods: A questionnaire was distributed to a convenience sample of Canadian physicians who diagnose chronic pain conditions. We divided participant data into three groups based on their FM education and the proportion of FM patients encountered in their field of practice to accurately determine knowledge and experience differences. Physician responses were scored and analyzed for their association with education and experience in FM.

Results: Physician knowledge of the 1990 and 2010 criteria was associated with their education and experience with FM. Physicians with the most FM education and experience did not indicate comprehensive knowledge of the criteria, scoring a mean of 91.5 from 133. Physicians had adequate knowledge of the FM treatments. Fifty-one percent of physicians used a set of criteria in their practice and only 38% used the 2010 criteria.

Discussion/Conclusions: Many physicians lack adequate knowledge of the FM diagnostic criteria and did not adhere to them. This is concerning since it is not congruent with evidence based medicine. Poor knowledge of criteria may increase time for diagnosis and chances of misdiagnosis. Knowledge translation strategies should be implemented to address this problem. Future studies should aim to develop reliable biomarkers diagnostic of FM, rather that relying on solely symptom-based criteria.

## Localization of rat masticatory muscle mechano-nociceptors to tender points

Hayes Wong^a^, and B. E. Cairns^a,^^b^

^a^Faculty of Pharmaceutical Sciences, University of British Columbia, Vancouver, BC, Canada; ^b^Center for Neuroplasticity and Pain, SMI, Department of Health Science and Technology, Faculty of Medicine, Aalborg University, Aalborg, Denmark

**CONTACT** Hayes Wong hayes.wong@ubc.ca

© 2017 Hayes Wong and Brian E. Cairns. Published with license by Taylor & Francis.

This is an Open Access article distributed under the terms of the Creative Commons Attribution License (http://creativecommons.org/licenses/by/3.0/), which permits unrestricted use, distribution, and reproduction in any medium, provided the original work is properly cited. The moral rights of the named author(s) have been asserted.

Introduction/Aim: Tender points are muscle regions of increased pain sensitivity and occur at muscle-tendon junctions and around joints. We investigated whether mechano-nociceptors are localized to these areas in the masticatory muscles.

Methods: Electrophysiology: Female rats were anesthetized and placed in stereotaxic frame. A recording electrode was lowered into the trigeminal ganglion. Temporalis and masseter muscle mechano-nociceptors were identified (n = 64). Their receptive fields (RFs) were mapped and their mechanical thresholds (MTs) were measured with an electronic von Frey hair (EVF).

Behavior: MTs were measured with an EVF in 6 female rats

Results: Electrophysiology: The RFs of mechanonociceptors were found primarily near the muscle attachment areas to the jaw bone and the zygomatic arch. Localization was greatest near the three muscle insertion sites of the temporalis, anterior and posterior masseter muscles. The MTs of the mechano-nociceptors at these spots were 39.6 ± 8.0g (n = 10), 47.9 ± 12.2g (n = 11) and 41.1 ± 8.8g (n = 9) respectively. MTs of the other mechano-nociceptors was 36.7 ± 4.5g (n = 34). Conduction velocities (CV) of fibers from the three sites combined was 8.2 ± 0.7m/s, compared to 9.8 ± 0.8m/s from the rest of the fibers.

Behavior: Compared to the belly of the masseter muscle (MT = 32.6 ± 2.4g), muscle insertion sites of the temporalis, anterior and posterior masseter muscles were more sensitive with MTs of 23.1 ± 1.7g, 19.2 ± 1.3g and 25.4 ± 1.4g, respectively.

Discussion/Conclusions: Masticatory muscle mechano-nociceptor innervation appears to be increased at muscle insertion sites. This may explain the increased sensitivity of female rats to mechanical stimulation in these areas. This finding may be useful in investigating the physiological characteristics of tender points.

## Establishing the psychometric properties of the OUCHI tool during two-month vaccinations

Hannah Gennis^a^, Shaylea Badovinac^a^, Rebecca Pillai Riddell^a,^^b^, Saul Greenberg^c,^^e^, and Hartley Garfield^d^

^a^Psychology, York University, Toronto, Ontario, Canada; ^b^Psychiatry, University of Toronto, Toronto, Ontario, Canada; ^c^Psychiatry Research The Hospital for Sick Children, Toronto, Ontario, Canada; ^d^Paediatrics, University of Toronto, Toronto, Ontario, Canada; ^e^The Hospital for Sick Children, Paediatric Medicine, Toronto, Ontario, Canada

**CONTACT** Hannah Gennis hgennis@yorku.ca

© 2017 Hannah Gennis, Shaylea Badovinac, Rebecca Pillai Riddell, Saul Greenberg, and Hartley Garfield. Published with license by Taylor & Francis.

This is an Open Access article distributed under the terms of the Creative Commons Attribution License (http://creativecommons.org/licenses/by/3.0/), which permits unrestricted use, distribution, and reproduction in any medium, provided the original work is properly cited. The moral rights of the named author(s) have been asserted.

Introduction/Aim: The OUCHI Tool is a checklist of eight suboptimal parent behaviours that increase infant distress during vaccination. While the OUCHI Tool has been shown to be reliable and valid at 12 months (Pillai Riddell, Gennis, et al., submitted), our goal was to establish its basic psychometric properties during 2-month vaccinations.

Methods: Using archival footage (OUCH Cohort: 760 infants followed over vaccinations between 2 months and 5 years), 81 two-month infant-parent dyads were coded that had also been coded at 6- and 12-month vaccination appointments. Divergent and convergent validity relationships were assessed using EAS (Biringen, 2008), and NFCS (Grunau & Craig, 1987) and MBPS (Taddio et al., 1995), respectively. Test-retest reliability with the 6- and 12-month OUCHI scores was also assessed.

Results: The OUCHI Tool coded on parents during the 2-month vaccination showed interrater reliability (*r’s* = 0.73 to 0.98), test-retest reliability with parent OUCHI scores at 6-month (*r* = 0.24) and 12-month (*r* = 0.23) vaccinations, divergent validity with EAS (*r* = −0.17, *d* = 0.35), and convergent validity with NFCS one minute (*r* = 0.32, *d* = 0.68), two minutes, (*r* = 0.42, *d* = 0.93), and three minutes post-needle (*r* = 0.18, *d* = 0.37), and MBPS one minute (*r* = 0.51, *d* = 1.19), two minutes (*r =* 0.32, *d* = 0.68), and three minutes (*r* = 0.32, *d* = 0.68) post-needle.

Discussion/Conclusions: The OUCHI Tool appears to show promising reliability and validity to assess suboptimal parent behaviours with infants who are undergoing the 2-month vaccinations.

## Suppression of inflammatory responses in lipoteichoic acid- and peptidoglycan-induced human tonsil epithelial cells by phytochemical-rich medicinal plant extracts

Niluni M. Wijesundara^a^, Satvir Sekhon-Loodu^b^, and H.P. Vasantha Rupasinghe 0000-0003-3435-0052^b,^^c^

^a^Department of Biology, Dalhousie University, Halifax, Nova Scotia, Canada; ^b^Department of Plant, Food, and Environmental Sciences, Dalhousie University, Truro, Nova Scotia, Canada; ^c^Department of Biology, and Department of Pathology, Dalhousie University, Truro and Halifax, Nova Scotia, Canada

**CONTACT** Niluni M. Wijesundara niluniw@dal.ca

© 2017 Niluni M. Wijesundara, Satvir Sekhon-Loodu, and H. P. Vasantha Rupasinghe. Published with license by Taylor & Francis.

This is an Open Access article distributed under the terms of the Creative Commons Attribution License (http://creativecommons.org/licenses/by/3.0/), which permits unrestricted use, distribution, and reproduction in any medium, provided the original work is properly cited. The moral rights of the named author(s) have been asserted.

Introduction/Aim: There is a growing interest in discovering plant-based anti-inflammatory compounds as potential alternatives to conventional drugs. The aim of this study was to evaluate anti-inflammatory activity of phytochemical-rich extracts prepared from 12 herbal plants, often cited in Canadian traditional medicine for the treatment of pain associated with streptococcal pharyngitis, using human tonsil epithelial cells (HTonEpiC) *in vitro*.

Methods: Twenty-seven phytochemical-rich extracts were evaluated for the anti-inflammatory effects using HTonEpiC model. The HTonEpiC were triggered by a mixture of lipoteichoic acid (LTA) and peptidoglycan (PGN) (10 µg/mL) for 4 h and then were incubated with ethanol extracts (EE) or aqueous extracts (AE) for 20 h. The secretion of four pro-inflammatory cytokines in cell-free supernatants were measured using enzyme-linked immunosorbent assays (ELISA). Total phenolic and total flavonoid contents of the extracts were determined using spectrophotometric methods.

Results: The herbal plant extracts (≤ 5 μg/mL) were not cytotoxic to HTonEpiC. The extracts exhibited a broad range of reduction (1.2% to 92.6%) of secretion of interleukin-8 (IL-8), human beta defensin-2 (hBD-2), epithelial-derived neutrophil activating protein-78 (ENA-78) and granulocyte chemotactic protein-2 (GCP-2). The danshen root EE and both EE and AE of clove, ginger and echinacea flower exhibited the greatest anti-inflammatory activity *in vitro* where they were signiﬁcantly suppressed the secretion of all tested pro-inflammatory cytokines.

Discussion/Conclusions: The most efficacious, danshen root, clove, ginger and echinacea flowers extracts exhibits therapeutic potential for developing natural health products in the management of painful inflammation due to streptococcal pharyngitis.

## Associations between infant heart rate variability during vaccination and temperament at 12 months of age

Jordana A. Waxman^a^, Miranda G. DiLorenzo^a^, Rebecca Pillai Riddell^a^, and Hartley Garfield^b^

^a^Clinical-Developmental Psychology, York University, Toronto, Ontario, Canada; ^b^Pediatrics, University of Toronto, Toronto, Ontario, Canada

**CONTACT** Jordana A. Waxman waxmanja@yorku.ca

© 2017 Jordana A. Waxman, Miranda G. DiLorenzo, Rebecca Pillai Riddell, and Hartley Garfield. Published with license by Taylor & Francis.

This is an Open Access article distributed under the terms of the Creative Commons Attribution License (http://creativecommons.org/licenses/by/3.0/), which permits unrestricted use, distribution, and reproduction in any medium, provided the original work is properly cited. The moral rights of the named author(s) have been asserted.

Introduction/Aim: To provide preliminary data on the relationship between healthy 12-month old infants’ heart rate variability during vaccination and infant temperament.

Methods: The data is part of an ongoing longitudinal study that follows caregivers and infants through the second year of life (12, 18 and 24 months; *N=* 51) during their well baby visits. A measure of infant temperament (Early Childhood Behaviour Questionnaire) was collected prior to the vaccination. Cardiac data was collected for 1-minute baseline and 2-minutes post-vaccination. Respiratory sinus arrhythmia (RSA) values were calculated on sequential 30-s epochs for the baseline and post-vaccination periods. Bivariate Pearson’s correlations were used to test the relationship between infant temperament and RSA during the vaccination.

Results: Infant RSA increased from baseline (*M =* 3.83, SD = 1.13) to 1-minute (*M =* 4.3, *SD* = 1.45) and 2-minutes (M = 4.37, SD = .96) post-vaccination. Infant RSA at 1-minute post-vaccination was associated with the Negative Affect subscale (*r* = -.664, *p* = .01), while infant RSA at 2-minutes post-vaccination was associated with the Surgency subscale, a measure of positive emotionality, activity level, impulsivity and risk-taking (*r* = −.543 = .04).

Discussion/Conclusions: For the first time in the literature, correlation data are provided on infant physiological response to immunization pain and temperamental measures at 12 months of age. Additionally, this study provides preliminary evidence that physiological response to vaccination pain may be reflective of parental report of infant temperament outside of the pain context.

## Do I get by with a little help from my friend? Attending and non-attending behaviours in response to pain

Paula Forgeron 0000-0002-4686-9698^a^, Christine Chambers^b^, Janice Cohen^c^, Bruce Dick^d^, Christine Lamontagne^e^, and G. Allen Finely^f^

^a^School of Nursing, University of Ottawa, Ottawa, ON, Canada; ^b^Pediatrics and Psychology & Neuroscience, IWK Health Centre/Dalhousie University, Halifax, NS, Canada; ^c^Behavioural Neurosciences and Consultation Liaison Team, Children’s Hospital of Eastern Ontario, Ottawa, ON, Canada; ^d^Anesthesiology and Pain Medicine, Psychiatry & Pediatrics, Stollery Children’s Hospital/University of Alberta, Edmonton, Alberta, Canada; ^e^Anesthesiology, CHEO/University of Ottawa, Ottawa, ON, Canada; ^f^Anesthesia and Psychology, IWK Health Centre/Dalhousie University, Halifax, NS, Canada

**CONTACT** Paula Forgeron paula.forgeron@uottawa.ca

© 2017 Paula Forgeron, Christine Chambers, Janice Cohen, Bruce Dick, Christine Lamontagne, and G. Allen Finely. Published with license by Taylor & Francis.

This is an Open Access article distributed under the terms of the Creative Commons Attribution License (http://creativecommons.org/licenses/by/3.0/), which permits unrestricted use, distribution, and reproduction in any medium, provided the original work is properly cited. The moral rights of the named author(s) have been asserted.

Introduction/Aim: To determine the use of verbal behaviors during pain between teen friendship dyads when a member has chronic pain compared to healthy friendship dyads.

Methods: An observational multisite study using the cold pressor task was conducted with 61 dyads (30 with a chronic pain teen). Data consisted of self-report friendship features (e.g. satisfaction), pain (e.g. intensity, duration), and observed behaviors. Measure and task completion order were controlled. Videos were coded for attending (e.g. symptom related talk, reassurance) and non-attending (e.g. distraction) verbal behaviors for both dyad members as cold pressor participant and observing friend.

Results: Friendship satisfaction significantly correlated with the friends’ use of attending behaviors (−.217) and non-attending (.218). Regression 1: controlling for age and sex 12.5% of the variance in pain intensity was predicted by participants’ use of attending behaviors. Regression 2: controlling for age and sex 16.9% of the variance in pain duration was predicted by the participants’ use of non- attending behaviors. Analysis of variance revealed teens in the chronic pain dyad used significantly fewer non-attending behaviors during the cold pressor compared healthy teen dyads.

Discussion/Conclusions: Teens with chronic pain and their friends use fewer non-attending behaviors when experiencing pain compared to healthy teens. Perhaps friends of teens with chronic pain are influenced by their friend’s reaction to pain over time, which may change their natural behaviors from more non-attending to attending. Determining ways to help teens with chronic pain use more non-attending behaviors when spending time with their friends may increase their friends’ use of non-attending behaviors.

## Chronic disruptive pain in emerging adults with and without chronic health conditions: The moderating role of psychiatric disorders

Rana Qadeer^a^, Lilly Shanahan^b^, and Mark Ferro^c^

^a^Health Research Methods, Evidence, and Impact, McMaster University, Hamilton, Ontario, Canada; ^b^Psychology, University of Zürich, Zürich, Canton of Zürich, Switzerland; ^c^School of Public Health and Health Systems, University of Waterloo, Waterloo, Ontario, Canada

**CONTACT** Rana Qadeer qadeerrm@gmail.com

© 2017 Rana Qadeer, Lilly Shanahan, and Mark Ferro. Published with license by Taylor & Francis.

This is an Open Access article distributed under the terms of the Creative Commons Attribution License (http://creativecommons.org/licenses/by/3.0/), which permits unrestricted use, distribution, and reproduction in any medium, provided the original work is properly cited. The moral rights of the named author(s) have been asserted.

Introduction/Aim: There has been a growth in the proportion of emerging adults vulnerable to pain-related sequelae of chronic health conditions (CHCs). Given the lack of research during this important developmental period, this study investigated the association between CHCs and chronic disruptive pain (CDP) among emerging adults and the extent to which psychiatric disorders moderate this association.

Methods: The data come from the 2012 Canadian Community Health Survey – Mental Health. Respondents were 15–30 years of age (n = 5987). Odds ratios (OR) and 95% confidence intervals (CI) were computed from ordinal logistic regression models adjusting for sociodemographic covariates. Product-term interactions between CHCs and psychiatric disorders were included in the models to explore the moderating effects.

Results: Compared to controls, participants with CHCs had greater odds of reporting CDP (OR = 4.94, 95% CI = 4.08–5.99). Alcohol (β = −0.66; p = 0.025) and drug abuse/dependence disorders (β = −1.24; p = 0.012) were found to moderate the association between CHCs and CDP. Specifically, the probability of CDP was higher for emerging adults without CHCs and with alcohol or drug disorders; however, among participants with CHCs, risk was higher for respondents without these disorders.

Discussion/Conclusions: Conclusively, there is a robust association between CHCs and CDP. The moderating effects suggest that alcohol or drug disorders are especially harmful for young adults without CHCs and contribute to higher levels of CDP; however, among those with CHCs, alcohol and illicit drugs may be used as a numbing agent to blunt CDP. Therefore, healthcare providers should be proactive in screening for psychiatric disorders and should support young people with CHCs to minimize pain-related impact.

## Pain self-efficacy mediates the relation between perceived injustice, depressive symptoms, pain, disability and occupational outcomes in individuals with whiplash injuries

Esther Yakobov^a^, Junie S. Carriere^a^, Whitney Scott^b^, and Michael J.L. Sullivan^a^

^a^Psychology, McGill University, Montreal, Quebec, Canada; ^b^Psychology, King’s College London, Institute of Psychiatry, Psychology, and Neuroscience, London, United Kingdom

**CONTACT** Esther Yakobov estheryakob@gmail.com

© 2017 Esther Yakobov, Junie S. Carriere, Whitney Scott, and Michael J.L. Sullivan. Published with license by Taylor & Francis.

This is an Open Access article distributed under the terms of the Creative Commons Attribution License (http://creativecommons.org/licenses/by/3.0/), which permits unrestricted use, distribution, and reproduction in any medium, provided the original work is properly cited. The moral rights of the named author(s) have been asserted.

Introduction/Aim: Extant research indicates that perceived injustice is a risk factor for complicated trajectories of recovery and lower rates of return to work in individuals with whiplash injuries. The mechanisms by which perceived injustice contributes to poor outcomes however are not well understood. Couched within the model of perceived unfairness discussed in psychological and epidemiological research, is the assumption that the experience of unfairness might impact on poor health outcomes via compromised beliefs in the ability to cope with adversity. No empirical research to date tested the relation between these variables in the context of chronic pain. The aim of the present study was to investigate whether reduced perception of pain self-efficacy mediated the relation between perceived injustice, pain, disability, depressive symptoms, and work related outcomes in individuals with whiplash injuries.

Methods: The study sample consisted of 104 individuals enrolled in a multidisciplinary treatment program designed to promote functional recovery following whiplash injury. They completed questionnaires prior to treatment, and provided information pertaining to pain and occupational outcomes at one year follow-up.

Results: The results of regression analyses revealed that reduced self-efficacy mediated the relation between perceived injustice, disability and depressive symptomatology before treatment. Reduced self-efficacy also mediated the relation between perceived injustice, pain and hours spent at work at one year follow-up.

Discussion/Conclusions: The results of the present study suggest that reduced self-efficacy might be one pathway by which perceived injustice contributes to poor health and employment outcomes in individuals with pain conditions. Clinical and theoretical implications are discussed.

## High pain catastrophizers are more accurate in estimating the duration of exposure to painful stimulation than low pain catastrophizers

Esther Yakobov, Jessica Dorval, and M. J.L. Sullivan

Psychology, McGill University, Montreal, Quebec, Canada

**CONTACT** Esther Yakobov estheryakob@gmail.com

© 2017 Esther Yakobov, Jessica Dorval, and Michael J.L. Sullivan. Published with license by Taylor & Francis.

This is an Open Access article distributed under the terms of the Creative Commons Attribution License (http://creativecommons.org/licenses/by/3.0/), which permits unrestricted use, distribution, and reproduction in any medium, provided the original work is properly cited. The moral rights of the named author(s) have been asserted.

Introduction/Aim: Pain catastrophizing has been conceptualized as a negative cognitive–affective response to anticipated or actual pain. Pain catastrophizing emerged as the most robust predictor of pain outcomes in clinical and experimental research. One well researched pathway by which catastrophizing is thought to augment pain intensity is excessive focus to pain symptoms, and impaired ability to divert attention from painful sensation. Attentional mechanisms have also been implicated in the construction of time during a painful experience. The relation between catastrophic cognitions and perceived duration of a painful event remains unexplored.

Methods: The aim of the present study was to explore the relation between pain catastrophizing, other pain coping strategies and perceived duration of time when in pain.

The study sample consisted of 135 undergraduate students. They completed questionnaires before and after painful stimulation and a neutral task of equal duration.

Results: The results revealed that participants perceived the duration of painful exposure to be shorter than the duration of a neutral task. Compared to low catastrophizers high catastrophizers perceived the duration of a painful procedure to be significantly longer. However, high catastrophizers were more accurate than low catastrophizers when estimating the duration of the painful procedure. During exposure to pain, high catastrophizers focused more on their pain, and were less able to suppress pain related thoughts.

Discussion/Conclusions: The results of the present study support the involvement of attentional mechanisms in perception of duration of painful experience, and suggest that high catastrophizers are more accurate in estimating time when in pain.

## The relationship between rate of algometer application and pain pressure threshold in the assessment of latent myofascial trigger points

Lukas D. Linde^a^, Maneil Joshi^b^, Dinesh A. Kumbhare^c^, and John Z. Srbely^d^

^a^Human Health and Nutritional Sciences, University of Guelph, Guelph, Ontario, Canada; ^b^School of Human Kinetics, University of Ottawa, Ottawa, Ontario, Canada; ^c^Department of Medicine, University of Toronto, Toronto, Ontario, Canda; ^d^Human Health and Nutritional Sciences, University of Guelph, Guelph, Ontario, Canada

**CONTACT** Lukas D. Linde llinde@uoguelph.ca

© 2017 Lukas D. Linde, Maneil Joshi, Dinesh A. Kumbhare, and John Z. Srbely. Published with license by Taylor & Francis.

This is an Open Access article distributed under the terms of the Creative Commons Attribution License (http://creativecommons.org/licenses/by/3.0/), which permits unrestricted use, distribution, and reproduction in any medium, provided the original work is properly cited. The moral rights of the named author(s) have been asserted.

Introduction/Aim: Pain pressure threshold (PPT) is a commonly employed technique in the assessment of myofascial pain syndrome (Gerwin, 2001). The effect of rate of application (RoA) of the pressure algometer on the raw PPT score is poorly understood. The purpose of this study was to investigate the relationship between RoA and raw PPT. We tested the hypothesis that a positive linear relationship exists between RoA and raw PPT.

Methods: Thirty-three young healthy individuals (21.90 ± 1.77 years, 171.33 ± 11.07 cm, 68.71 ± 12.31 kg) with an identified latent trigger point in the right infraspinatus muscle were recruited from the University of Guelph student population. Participants received three PPT assessments at a baseline rate (30N/s), followed by two assessment trials at each of three target rates: low (15N/s), medium (35N/s), and fast (55N/s). Real-time data were collected using a custom designed program in Labview (National Instruments, version 2013) and saved for post-hoc analysis. Pearson’s correlation coefficients and linear regression analysis were performed for each individual participant’s rate variable trials.

Results: The mean Pearson’s correlation coefficient between RoA and PPT demonstrated a strong, positive relationship (Pearson’s R Mean ± SD: 0.77 ± 0.19). The average slope of the linear regression between RoA and PPT was low (slope Mean ± SD: 0.13 ± 0.09).

Discussion/Conclusions: The RoA during PPT assessment is strongly correlated to the raw PPT score. Although our data demonstrates a robust relationship between RoA and PPT, the observed effect of rate on PPT (slope) was small. Further research should investigate the clinical relevance of changes in rate on the raw PPT score in a clinical population.

## Transcription of genes linked with fibromyalgia (CCT5) and temporomandibular dysfunction (MTTR) is genetically regulated in human endometrium

Jane E. Girling^a^, Sarah Holdworth-Carson^a^, Jenny N. Fung^b^, Premila Paiva^a^, Eliza M. Colgrave^a^, Martin Healey^a^, Joseph E. Powell^b^, Grant W. Montgomery^b^, and Peter A.W. Rogers^a^

^a^Gynaecology Research Centre, Department of Obstetrics and Gynaecology, The University of Melbourne and The Royal Women’s Hospital, Melbourne, Victoria, Australia; ^b^Institute of Molecular Bioscience, University of Queensland, Brisbane, Queensland, Australia

**CONTACT** Jane E. Girling jgirling@unimelb.edu.au

© 2017 Jane E. Girling, Sarah Holdworth-Carson, Jenny N. Fung, Premila Paiva, Eliza M. Colgrave, Martin Healey, Joseph E. Powell, Grant W. Montgomery, and Peter A. W. Rogers. Published by Taylor & Francis.

This is an Open Access article distributed under the terms of the Creative Commons Attribution License (http://creativecommons.org/licenses/by/3.0/), which permits unrestricted use, distribution, and reproduction in any medium, provided the original work is properly cited. The moral rights of the named author(s) have been asserted.

Introduction/Aim: Endometriosis, a complex gynaecological disease causing pelvic pain, is influenced by environmental and genetic factors. Genome-wide association studies have identified single-nucleotide polymorphisms associated with endometriosis. Pain perception also has a genetic component; however, studies have yet to consider pain genetics relative to endometrium and/or endometriosis. We aim to elucidate the interaction between endometriosis and pain genetics and associated effects on gene expression (expression quantitative trait loci [eQTL]).

Methods: We developed an extensive database of genetic, molecular and clinical information from women with/without endometriosis (Royal Women’s Hospital; n = 648). Blood samples were genotyped using Human CoreExome chips. Endometrial gene expression was generated using Illumina Human HT12v4.0 Beadchips. eQTL analysis was performed using recoded and imputed SNP genotypes (n = 123, minor allele dosage, linear regression models, covariate: menstrual phase). We then identified endometrial eQTLs previously associated with pain/pain conditions.

Results: We identified endometrial eQTLs for 198 genes (Fung et al. 2017. Human Reproduction). Of these, ‘chaperonin-containing TCP1, subunit 5 (epsilon)’ (CCT5) and ‘5-methyltetrahydrofolate-homocysteine methyltransferase reductase’ (MTRR) were reported as being in genetic association with fibromyalgia and temporomandibular disorder (TMD), respectively (Zorina-Lichtenwalter et al. 2016. Neuroscience).

Discussion/Conclusions: We have demonstrated genetic regulation of endometrial gene expression, including two genes in genetic association with pain conditions fibromyalgia (CCT5) and TMD (MTRR). Ongoing studies will explore mechanisms causing endometriosis pain; we will examine functions of eQTLs in endometriosis pathophysiology and utilize our extensive database to examine factors contributing to heterogeneous pain symptoms. Ultimately, we hope to provide tailored therapy to women based on pain phenotypes.

## Systematic review of risk factors for chronic postsurgical pain in children

Naiyi Sun^a^, Kathryn Birnie^b^, Fiona Campbell^a^, and Jennifer Stinson^b^

^a^Anesthesia and Pain Medicine, The Hospital for Sick Children, Toronto, Ontario, Canada; ^b^Child Health Evaluative Sciences, The Hospital for Sick Children, Toronto, Ontario, Canada

**CONTACT** Naiyi Sun naiyi.sun@sickkids.ca

© 2017 Naiyi Sun, Kathryn Birnie, Fiona Campbell, and Jennifer Stinson. Published with license by Taylor & Francis

This is an Open Access article distributed under the terms of the Creative Commons Attribution License (http://creativecommons.org/licenses/by/3.0/), which permits unrestricted use, distribution, and reproduction in any medium, provided the original work is properly cited. The moral rights of the named author(s) have been asserted.

Introduction/Aim: Chronic postsurgical pain (CPSP) is defined as pain that develops after surgery and lasts for at least 2 months with other causes for pain excluded. CPSP in children leads to emotional suffering and negatively impacts all aspects of health-related quality of life. The purpose of this systematic review is to identify biopsychosocial risk factors for the development and maintenance of CPSP in children and assess the quality of supporting evidence.

Methods: Relevant studies were identified by searching the electronic databases MEDLINE, EMBASE, CINAHL, PsychINFO and Web of Science by a librarian and references of included studies. Studies were included for the review if they met the following criteria: 1) inclusion of children and adolescents (0–18 years) undergoing any type of surgery; 2) assessment of pain at least 2 months after surgery; and 3) assessment of one or more potential risk factors for development of CPSP.

Results: The search strategy generated a total of 1777 unique references. Twelve studies met the inclusion criteria and were reviewed in greater detail for analysis. Assessed risk factors associated with CPSP included child age, preoperative and acute postoperative pain, anxiety, pain catastrophizing, and parental catastrophizing.

Discussion/Conclusions: Early identification of patients at risk for CPSP and awareness of the impact of psychological factors will allow for earlier intervention and potentially better pain outcomes. Due to the small number of current studies, there is a need for further larger scale well designed studies to investigate this topic.

## Post-operative pain control in a teaching hospital in Kigali, Rwanda: An observational study

Danyela Petra Lee^a^, Eugene Tuyishime^b^, Joseph Niyitegeka^b^, Paulin Banguti^b^, Theo Twagirumugabe^b^, and J. O’Brien^a^, William P. McKay 0000-0003-0128-5960^a^

^a^Department of Anesthesiology, University of Saskatchewan, Saskatoon, SK, Canada; ^b^Department of Anesthesia, University of Rwanda, Kigali, Kigali, Rwanda

**CONTACT** Danyela Petra Lee danyela.lee@usask.ca

© 2017 Danyela Petra Lee, Paulin Banguti, Theo Twagirumugabe, and William P. McKay. Published with license by Taylor & Francis.

This is an Open Access article distributed under the terms of the Creative Commons Attribution License (http://creativecommons.org/licenses/by/3.0/), which permits unrestricted use, distribution, and reproduction in any medium, provided the original work is properly cited. The moral rights of the named author(s) have been asserted.

Introduction/Aim: Pain is a global health problem. Effective post-operative analgesia (POA) is lacking in developing countries. We studied: 1) *Practice*: what is POA quality for patients at the University Teaching Hospital of Kigali (UTHK)? 2) *Perceptions*: are they satisfied with POA?

Methods: an observational study at UTHK. Consenting adult patients undergoing major abdominal, thoracic, or orthopedic surgery were recruited. On post-operative day 2, participants were administered the International Pain Outcomes (IPO) and a brief questionnaire to assess attitudes towards post-operative pain.

Results: 91 patients were recruited; 83 (91%) completed the questionnaire. *Practice*: 54% received pre-emptive analgesia; 8% received no POA. On average, the worst pain was 6/10 (±2) and the least 3/10 (±1). 44% (±22%) of the time patients were in severe pain, with an interference score of 5/10 (±2) in ability to do activities in bed. *Perceptions*: 54% (±21%) of patients felt they received adequate pain relief, and 71% felt more pain treatment was not necessary. 57% of patients felt pain should not be taken away completely, and 76% felt they should put up with some pain rather than complain.

Discussion/Conclusions: With 8% of patients receiving no POA, POA at UTHK would not meet generally accepted practice in Canada. That 71% of patients felt that POA was adequate despite an activity interference score of 5/10 may be due to cultural perceptions that pain is necessary and patients should not complain. These results can be used as a baseline to evaluate future interventions in pain management in Kigali.

## Untangling trigeminal neuralgia from neurovascular compression: The role of multimodal magnetic resonance imaging and microstructural diffusivity analysis

P. S. Hung, E. Wharton-Shukster, K. E. Liang, and Mojgan Hodaie

Division of Brain, Imaging & Behaviour, Krembil Research Institute, University Health Network, Toronto, Ontario, Canada

**CONTACT** Peter Shih-Ping Hung peter.hung@mail.utoronto.ca

© 2017 Peter Shih-Ping Hung, Erika Wharton-Shukster, Kevin E. Liang, and Mojgan Hodaie. Published with license by Taylor & Francis.

This is an Open Access article distributed under the terms of the Creative Commons Attribution License (http://creativecommons.org/licenses/by/3.0/), which permits unrestricted use, distribution, and reproduction in any medium, provided the original work is properly cited. The moral rights of the named author(s) have been asserted.

Introduction/Aim: Trigeminal neuralgia (TN) is a chronic neuropathic facial pain disorder characterized by severe, intermittent electric-like pain. Despite uniformly unilateral pain, TN patients have greater likelihood of bilateral trigeminal nerve neurovascular compression (NVC). NVC frequently, but not always coincides with pain. Moreover, NVC is unrelated to pain intensity and cannot, alone, fully explain TN’s clinical presentation of pain. To identify *in vivo* imaging signatures specific to TN pain, we used diffusion tensor imaging (DTI) to characterize symptomatic and asymptomatic trigeminal nerve microstructure in patients with bilateral NVC.

Methods: 3T T1 anatomical, 60-directions diffusion-weighted, and FIESTA images were acquired and aligned to diffusion space via ANTs for 27 surgically-naïve TN patients. NVCs were graded (I to III) with FIESTA images. Multi-tensor tractography was used to identify bilateral trigeminal nerves at the pontine segment, root entry zone, and cisternal point of maximal compression. From these regions, DTI-derived microstructural metrics—axial, radial, mean diffusivities, and fractional anisotropy (FA)—were extracted for linear mixed effects statistics between nerve types with significance set at p < 0.05.

Results: Across all NVC grades, asymptomatic nerves did not demonstrate diffusivity changes. Symptomatic nerves, however, displayed significant, grade-dependent variations in diffusivities. Compared to contralateral nerves, Grade I symptomatic nerves had higher FA (p < 0.01) at the point of maximal compression while Grade III symptomatic nerves had lower FA (p < 0.05) at the root entry zone.

Discussion/Conclusions: Microstructural diffusivities can be of significant value for distinguishing nerves with pain from those without pain and provide valuable insights into the pathophysiology of TN.

## Patients’ attitudes toward non-physician screening of low back and low back-related leg pain complaints referred for surgical assessment

Joshua Rempel^a^, Jason Busse^b^, Brian Drew^c^, Kesava Reddy^c^, Aleksa Cenic^c^, Edward Kachur^c^, Naresh Murty^c^, Henry Candelaria^d^, Ainsley E. Moore^e^, and John J. Riva^e^

^a^Medicine, McMaster University, Hamilton, Ontario, Canada; ^b^Anesthesia, McMaster University, Hamilton, Ontario, Canada; ^c^Surgery, McMaster University, Hamilton, Ontario, Canada; ^d^Rehabilitation, Oakville Trafalgar Memorial Hospital, Oakville, Ontario, Canada; ^e^Family Medicine, McMaster University, Hamilton, Ontario, Canada

**CONTACT** Joshua Rempel rempeljt@mcmaster.ca

© 2017 Joshua Rempel, Jason Busse, Brian Drew, Kesava Reddy, Aleksa Cenic, Edward Kachur, Naresh Murty, Henry Candelaria, Ainsley E. Moore, and John J. Riva. Published by Taylor & Francis.

This is an Open Access article distributed under the terms of the Creative Commons Attribution License (http://creativecommons.org/licenses/by/3.0/), which permits unrestricted use, distribution, and reproduction in any medium, provided the original work is properly cited. The moral rights of the named author(s) have been asserted.

Introduction/Aim: To explore patient attitudes towards screening to assess suitability for low back surgery by non-physician healthcare providers.

Methods: We administered a 19-item cross-sectional survey to adults with low back and/or low back- related leg pain who were referred for elective surgical assessment at one of five spine surgeons’ clinics in Hamilton, Ontario, Canada. The survey inquired about demographics, expectations regarding wait time for surgical consultation, as well as willingness to pay, travel, and be screened by non-physician healthcare providers.

Results: 80 low back patients completed our survey, for a response rate of 86.0% (80 of 93). Most respondents (72.5%; 58 of 80) expected to be seen by a surgeon within 3 months of referral, and 88.8% (71 of 80) indicated willingness to undergo screening with a non-physician healthcare provider to establish if they were potentially a surgical candidate. Half of respondents (40 of 80) were willing to travel >50 km for assessment by a non-physician healthcare provider, and 46.2% were willing to pay out-of-pocket (25.6% were unsure). However, most respondents (70.0%; 56 of 80) would still want to see a surgeon if they were ruled out as a surgical candidate, and written comments from respondents revealed concern regarding agreement between surgeons’ and non-physicians’ determination of surgical candidates.

Discussion/Conclusions: Patients referred for surgical consultation for low back or low back-related leg pain are largely willing to accept screening by non-physician healthcare providers. Future research should explore the concordance of screening results between surgeon and non-physician healthcare providers.

## Striking gold: Negotiating urine drug testing of chronic pain patients in primary care

Leslie Carlin^a^, Ruth Dubin^b^, John Flannery^c^, Naima Salemohamed^d^, Andrew Smith^e^, Paul Taenzer^f^, Jane Zhao^g^, and Andrea Furlan^g^

^a^Physical Therapy, University of Toronto, Toronto, ON, Canada; ^b^Family Medicine, Queens University, Kingston, ON, Canada; ^c^MSK& Multisystem Rehab Program, University Health Network, Toronto, ON, Canada; ^d^Institute of Health Policy, Management, and Evaluation, University of Toronto, Toronto, Ontario, Canada; ^e^Addiction Medicine Service, Centre for Addiction and Mental Health, Toronto, ON, Canada; ^f^Psychology, University of Calgary, Calgary, AB, Canada; ^g^ECHO Ontario, MSK Outpatient Department, Toronto Rehabilitation Institute, University Health Network, Toronto, Ontario, Canada

**CONTACT** Leslie Carlin Leslie.Carlin@utoronto.ca

© 2017 Leslie Carlin, Ruth Dubin, John Flannery, Naima Salemohamed, Andrew Smith, Paul Taenzer, Jane Zhao, and Andrea Furlan. Published with license by Taylor & Francis.

This is an Open Access article distributed under the terms of the Creative Commons Attribution License (http://creativecommons.org/licenses/by/3.0/), which permits unrestricted use, distribution, and reproduction in any medium, provided the original work is properly cited. The moral rights of the named author(s) have been asserted.

Introduction/Aim: Health care providers (HCPs) must concern themselves with their chronic pain patients’ possible abuse or misuse of psychoactive medications. Urine drug testing (UDT) offers ‘objective’, though fallible, evidence of substance consumption that may bolster or counter patients’ narratives. Mandating UDT as part of clinical care can stress the therapeutic alliance. Participants in Ontario’s ProjectECHO-Chronic Pain (‘ECHO’), a tele-mentoring intervention, described using UDT in their practices. This qualitative investigation explores these dialogues.

Methods: Data from six focus group discussions, twelve in-depth interviews, and two group discussions were read by two or more researchers, using a qualitative-descriptive analytic approach. These authors compared their analyses and discussed divergent interpretations until achieving consensus regarding meaningful, relevant themes.

Results: ECHO participants repeatedly voiced the challenges of ordering UDT for their pain patients on opioids. Some HCPs described discomfort or ‘feeling conflicted’ when requesting UDTs while others avoided doing them altogether. Others spoke about their attempts to negotiate a mutually acceptable ‘middle ground’. Doctors described patients who ‘fired’ them after ‘failing’ a UDT or after being asked to submit to one. Team-based pharmacists expressed concern over poorly defined roles and lack of clear communication between pharmacist and HCP.

Discussion/Conclusions: Requesting UDT for patients on prescription opioids is a sensible best-practice policy that assumes a mutual desire for patients’ well-being and longevity. The everyday practice of health care, however, implicitly constructs cultural definitions of well-being and appropriateness that may not be shared. Promoting trust and compliance within the therapeutic relationship requires effective communication to accompany testing.

## The role of parent anxiety in young children’s pain memory development following surgery

Lauren McCallum^a^, Maria Pavlova^a^, Jillian Vinall^b^, and Melanie Noel^a,^^c^

^a^Psychology, University of Calgary, Calgary, Alberta, Canada; ^b^Anesthesia, University of Calgary, Calgary, Alberta, Canada; ^c^Behaviour & Developing Brain, Alberta Children’s Hospital Research Institute, Calgary, Alberta, Canada

**CONTACT** Lauren McCallumlemccall@ucalgary.ca

© 2017 Lauren McCallum, Maria Pavlova, Jillian Vinall, and Melanie Noel. Published with license by Taylor & Francis.

This is an Open Access article distributed under the terms of the Creative Commons Attribution License (http://creativecommons.org/licenses/by/3.0/), which permits unrestricted use, distribution, and reproduction in any medium, provided the original work is properly cited. The moral rights of the named author(s) have been asserted.

Introduction/Aim: Children’s memories of pain are a robust predictor of increased pain and distress at subsequent painful experiences and future health behaviours. Research suggests that parent catastrophizing leads to adolescents developing negatively biased memories for post-surgical pain. This study is the first to examine the relationship between parent and child anxiety prior to surgery and young children’s subsequent memories for pain.

Methods: Thirty children aged 4–7 undergoing tonsillectomies, and their parents were recruited. Two hours prior to surgery, parents completed the State-Trait Anxiety Inventory, and reported their children’s anxiety levels on a numerical rating scale. The Modified Yale Preoperative Anxiety Scale was used to assess children’s behavioural anxiety. Post-surgery and again one month later, parents and children reported on child pain using the Faces Pain Scale-Revised.

Results: Hierarchical regression analyses revealed that children of parents with higher state anxiety had more negatively estimated pain memories than children of less anxious parents, after controlling for child age and sex, and initial pain ratings. Parent anxiety accounted for 22.7% of the variance in child pain memories above and beyond the controlled variables (R^2^Δ = 0.227, p < .01), while the overall model accounted for 38.3% of the variance. Child anxiety was not significantly related to pain memory biases.

Discussion/Conclusions: Parental, but not child, anxiety was found to lead to negative biases in young children’s pain memories which parallels findings linking parent catastrophizing to adolescents’ post-surgical pain memories. Findings suggest parental anxiety may be a fruitful target for intervention to minimize children’s pain memory biases.

## Using a humanoid robot to reduce procedural pain in children with cancer: A pilot randomized controlled trial (RCT)

Carley Ouellette^a^, Jennifer N. Stinson^b^, Paul. C. Nathan^b^, Lindsay A. Jibb^c^, Vanessa Hum^b^, and Tanya Beran^d^

^a^Nursing, University of Western Ontario, London, Ontario, Canada; ^b^Child Health Evaluative Sciences, The Hospital for Sick Children, Toronto, Ontario, Canada; ^c^Nursing, University of Ottawa, Ottawa, Ontario, Canada; ^d^Community Health Sciences, University of Calgary, Calgary, Alberta, Canada

**CONTACT** Carley Ouellette carley.ouellette@sickkids.ca

© 2017 Carley Ouellette, Jennifer N. Stinson, Paul. C Nathan, Lindsay A. Jibb, Vanessa Hum, and Tanya Beran. Published with license by Taylor & Francis.

This is an Open Access article distributed under the terms of the Creative Commons Attribution License (http://creativecommons.org/licenses/by/3.0/), which permits unrestricted use, distribution, and reproduction in any medium, provided the original work is properly cited. The moral rights of the named author(s) have been asserted.

Introduction/Aim: Children with cancer often cite needle insertions into a subcutaneous port (SCP) as painful and frightening, even with topical anesthetic. We developed an evidence-based program employed on a humanoid robot that uses interactive movements and vocalizations to coach children through SCP access. Research objectives were to determine the feasibility and obtain preliminary estimates of effectiveness of the robot on pain and anxiety in children undergoing SCP access.

Methods: 40 children with cancer (4–9 years) who reported pain during previous SCP access, were randomized to receive either: intervention (robot using pre-programmed series of coaching behaviours before, during, and after procedure; N = 16) or active control (robot moving only; N = 24) during needle insertion by a nurse.

Results: Mean age was 6.15 years and 40% were female. Study accrual rate was 98%. Parents reported high satisfaction: 62% thought that MEDi was “very helpful” in reducing their child’s pain. Across both control and intervention groups, 81% reported decreased pain in the presence of MEDi. In addition, 67% of children enjoyed the presence of MEDi “very much.” There was a trend that children in intervention group reported less pain from previous SCP access (p-value = 0.08) compared to those in the control group. The technical difficulties that occurred which caused a disturbance during the intervention was 82% compared to the control group at 18%.

Discussion/Conclusions: Implementation of this study protocol is feasible but the robot’s SCP program would need to be refined in the intervention group before a future definitive RCT is conducted.

## Mentorship in anesthesia: A survey of perspectives among Canadian anesthesia residents

Suzan Ergun, Jason W. Busse, and Anne K. Wong

Anesthesia, McMaster University, Hamilton, Ontario, Canada

**CONTACT** Suzan Ergun suzan.ergun@gmail.com

© 2017 Suzan Ergun, Jason W. Busse, and Anne K. Wong. Published with license by Taylor & Francis.

This is an Open Access article distributed under the terms of the Creative Commons Attribution License (http://creativecommons.org/licenses/by/3.0/), which permits unrestricted use, distribution, and reproduction in any medium, provided the original work is properly cited. The moral rights of the named author(s) have been asserted.

Introduction/Aim: Mentorship is important for professional and academic growth; however, the role of mentorship in anesthesia is still being defined. We surveyed Canadian anesthesia residents to explore their perceptions of mentorship relationships.

Methods: We administered a 20-item cross-sectional survey to program directors and anesthesia residents in all Canadian departments of anesthesia. Program directors were asked about their mentorship programs, and residents were asked about their perceptions of benefits and barriers to effective mentoring.

Results: Sixteen of 17 (94%) program directors and 189 of 585 (32%) anesthesia residents responded to our survey. While 143 of 180 (79%) residents agreed that mentorship was beneficial to overall success as an anesthesiologist, only 11 of 16 (69%) program directors reported formal mentorship as part of their residency program, and only 119 of 189 (63%) residents reported access to a mentor. Barriers reported by residents included insufficient time with mentors, lack of formalized meeting times and objectives, mentor-mentee incompatibility (personal or professional), and lack of resident choice in mentor selection.

Discussion/Conclusions: Our study confirms that, despite positive perceptions among residents, mentorship remains underutilized in anesthesia programs. We identify barriers to effective mentorship, including the need to consider resident choice as a means to improve formal anesthesia mentorship programs.

## School functioning in children with chronic pain: A review and preliminary model

Kailyn Jones^a^, and Melanie Noel^b^

^a^Werklund School of Education, University of Calgary, Calgary, Alberta, Canada; ^b^Department of Psychology, University of Calgary, Calgary, Alberta, Canada

**CONTACT** Kailyn Jones kailyn.jones@ucalgary.ca

© 2017 Kailyn Jones and Melanie Noel. Published with license by Taylor & Francis.

This is an Open Access article distributed under the terms of the Creative Commons Attribution License (http://creativecommons.org/licenses/by/3.0/), which permits unrestricted use, distribution, and reproduction in any medium, provided the original work is properly cited. The moral rights of the named author(s) have been asserted.

Introduction/Aim: School is often cited as the “work” of childhood, and serves not only as an imperative site for the development of academic and cognitive skills but also for the development of identity, independence and social relationships. Elevated school absences in children with chronic pain are well documented; however, little about how pain effects school success beyond poor attendance is known. The developmental consequences and financial costs of school impairment are extensive with these children often achieving poorer grades, requiring costly special education services, and sometimes requiring full time homebound instruction. The aim of this study was to review what is known about school functioning in this population and propose a preliminary model to explain the phenomenon.

Methods: Using a set of predetermined inclusion criteria, key databases were searched for articles in peer-reviewed journals published since 2000. Articles were then reviewed and synthesized into the model.

Results: Studies were revealed to include measurement across variables from four key domains of functioning: (1) psychological factors (i.e., depressive and anxious symptoms); (2) social factors (i.e., peer relationships, child perceptions of teacher support and parent responses); (3) physiological factors (i.e., sleep disturbances), and (4) cognitive factors (i.e., perceived and actual disruption in divided attention and working memory).

Discussion/Conclusions: This review and preliminary model may serve as a tool to summarize factors that explain poor school functioning in children with chronic pain. The model also highlights a number of factors beyond pain intensity that should be considered when supporting this population in their schooling.

## Preoperative oral methadone for patients undergoing cardiac surgery: Reduction of postoperative pain

Timothy Bolton^a^, Sarah Chomicki^a^, William McKay^a^, Ryan Pikaluk^a^, John Tsang^b^, and Jeff Betcher^a^

^a^Anesthesiology, Perioperative Medicine and Pain Management, University of Saskatchewan, Saskatoon, Saskatchewan, Canada; ^b^Cardiac Surgery, University of Saskatchewan, Regina, Saskatchewan, Canada

**CONTACT** Timothy Bolton tim.bolton@usask.ca

© 2017 Timothy Bolton, Sarah Chomicki, William McKay, Ryan Pikaluk, John Tsang, and Jeff Betcher. Published with license by Taylor & Francis.

This is an Open Access article distributed under the terms of the Creative Commons Attribution License (http://creativecommons.org/licenses/by/3.0/), which permits unrestricted use, distribution, and reproduction in any medium, provided the original work is properly cited. The moral rights of the named author(s) have been asserted.

Introduction/Aim: Inadequately controlled sternotomy pain after cardiac surgery can lead to delayed recovery. Preoperative intravenous methadone is effective for reducing postoperative pain and opioid consumption. This initial pilot study investigated the effect of oral preoperative methadone on analgesia requirements, pain scores, and side effects.

Methods: Patients undergoing isolated coronary artery bypass graft (CABG) (n = 21) were randomized to receive methadone (0.3 mg/kg) or placebo prior to entering the operating room. Morphine requirements using patient-controlled analgesia (PCA) and Visual Analogue Scale (VAS) pain scores Q12H were recorded for 72 hours. Time to extubating, level of sedation, nausea, vomiting, pruritus, hypoventilation and hypoxia were also measured.

Results: Postoperative morphine during the first 24 hours was reduced by a mean of 23mg in the methadone group (mean difference [99%CI], -23 [37 to 13] mg; P < 0.005). Reduction in pain scores were not significant (P = 0.08). No reduction in PCA morphine or pain scores was observed beyond 24 hours postoperatively. The incidence of opioid related side effects was not increased throughout the postoperative period.

Discussion/Conclusion: Preoperative oral methadone reduced morphine requirements in the first 24 hours post CABG with no difference in opioid-related side effects. It could be conveniently given prior to starting an intravenous line as an effective, low cost, preoperative medication for cardiac surgery patients. A larger multi-centre study is planned.

## Predictors of opioid misuse following prescription of opioids for chronic non-cancer pain: A systematic review and meta-analysis of observational studies

Regina Li^a^, Li Wang^b^, Pat Hong^c^, Alka Kaushal^b^, Vahid Ashoorion^d^, Yasir Rehman^b^, Yaping Chang^d^, Kyle De Oliveira^e^, Anna Goshua^f^, Stephanie Ross^d^, Raad Yameen^g^, Rachel Couban^b^, Samantha Craigie^b^, and Jason W. Busse^b^

^a^Medicine, McMaster University, Hamilton, ON, Canada; ^b^Anesthesia, McMaster University, Hamilton, Ontario, Canada; ^c^Medicine, University of Ottawa, Ottawa, ON, Canada; ^d^Health Research Methods, Evidence, and Impact, McMaster University, Hamilton, Ontario, Canada; ^e^School of Medicine and Medical Science, University College Dublin, Dublin, Lienster, Ireland; ^f^Faculty of Health Sciences, McMaster University, Hamilton, Ontario, Canada; ^g^Institute of Medical Science, University of Toronto, Toronto, ON, Canada

**CONTACT** Regina Li regina.li@medportal.ca

© 2017 Regina Li, Li Wang, Pat Hong, Alka Kaushal, Vahid Ashoorion, Yasir Rehman, Yaping Chang, Kyle De Oliveira, Anna Goshua, Stephanie Ross, Raad Yameen, Rachel Couban, Samantha Craigie, and Jason W. Busse. Published with license by Taylor & Francis.

This is an Open Access article distributed under the terms of the Creative Commons Attribution License (http://creativecommons.org/licenses/by/3.0/), which permits unrestricted use, distribution, and reproduction in any medium, provided the original work is properly cited. The moral rights of the named author(s) have been asserted.

Introduction/Aim: Identification of patients with chronic non-cancer pain (CNCP) at higher risk for opioid misuse would be helpful to guide prescribing practices. We conducted a systematic review and meta-analysis of observational studies to explore factors associated with misuse among patients prescribed opioids for CNCP.

Methods: We searched MEDLINE, EMBASE, CINAHL, and PsycINFO databases through May 25^th^, 2016, to identify cohort or case-control studies that reported, in an adjusted analysis, the association between risk factors and misuse following opioid prescription for CNCP. We pooled estimates of association using random effects models for all independent variables reported by >1 study. We reported relative measures of association as pooled odds ratios (ORs), and absolute measures of association as the pooled absolute risk increase (ARI).

Results: Ten studies, involving 38,034 patients, reported 34 independent variables associated with opioid misuse. High quality evidence demonstrated increased odds of misuse with younger age (OR for every 10-year decrement 1.37, 95%CI 1.24 to 1.52), previous or current mental disorders (OR 1.50, 95%CI 1.24 to 1.81), previous or current substance use disorder (OR 4.02, 95%CI 2.51 to 6.43), and higher morphine equivalent daily dose (MED) (≥50 vs <50mg, OR 2.58, 95%CI 2.01 to 3.31). Given a baseline absolute risk of 24% for opioid misuse, the ARI varied from 6% (age) to 32% (substance abuse).

Discussion/Conclusions: Opioid misuse among patients using opioids for CNCP is common, and higher rates are associated with younger age, previous or current mental disorders or substance use disorder, and higher MED of prescribed opioid.

## Pilot study: Ketamine injection safety and efficacy in general practice for unresponsive chronic pain

M. Montbriand^a^, Janice Montbriand 0000-0003-0529-8507^b^

^a^Lakeshore Medical Clinic, Regina, SK, Canada; ^b^Anesthesia and Pain Management, Toronto General Hospital, Toronto, Ontario, Canada

**CONTACT** M. Montbriand admin@painmuse.org

© 2017 M. Montbriand and Janice Montbriand. Published with license by Taylor & Francis.

This is an Open Access article distributed under the terms of the Creative Commons Attribution License (http://creativecommons.org/licenses/by/3.0/), which permits unrestricted use, distribution, and reproduction in any medium, provided the original work is properly cited. The moral rights of the named author(s) have been asserted.

Introduction/Aim: Ketamine is a general anesthetic with NMDA receptor blocking properties. IV infusions have been found to hold promise in chronic pain and depression. The aim of the present study is to determine the safety and efficacy of ketamine in general practice, through the use of a previously established protocol using repeated shoulder intra-muscular (IM) injections to simulate an infusion.

Methods: 14 adults in this pilot (75% female, *M*_age_ = 47.5 *SD* = 12.6) with refractory pain (31% widespread pain, 31% moderate to high neuropathic symptoms) were treated at the medical clinic. Patients were given IM injections of 5–10 mgs of ketamine every 15 minutes. 54% of participants received two injections 15 minutes apart, while those with severe depression (Beck > 30) or extreme pain received three injections. Participants completed questionnaires assessing pain, depression, neuropathic symptoms, effect of ketamine on pain, and the length of this effect.

Results: Prior to injection, average pain levels were high (7.0/10 (range: 4–9)). After injection, pain decreased an average of 2.8 (range 2–4, *p* < .01) for a median of three days (range .1–4.4). Side effects were short term (30 minutes or less post-injection) and generally minor. Intoxication and incoordination regularly occurred but often improved with repeated injections. One patient had nausea, and rarely (2 patients) mild concentration difficulty was seen for some hours.

Discussion/Conclusions: In general practice, repeated small dose ketamine shots can be helpful and safe for uncontrolled pain. Side effects were minor and short term.

## Opioids for chronic non-cancer pain: A systematic review of randomized controlled trials

Jason W. Busse^a^, Li Wang^a^, Mostafa Kamal el Din^b^, Samantha Craigie^a^, Luis Montoya^c^, Sohail M. Mulla^b^, John J. Riva^b^, Luciane C. Lopes^d^, Nicole Vogel^e^, Eric Chen^f^, Karin Kirmayr^g^, Karin De Olivera^h^, Lori Oliveri^a^, Alka Kaushal^b^, Luis Chaparro^i^, Inna Oyberman^i^, Arnav Agarwal^j^, Ludwig Tsoi^k^, Tommy Lam^l^, Per O. Vandvik^m^, Rachel Couban^a^, Sandy Hsu^b^, Malgorzata Bala^n^, Stefan Schandelmaier^b^, Anne Scheidecker^o^, Shanil Ebrahim^b^, Bradley Johnston^p^, Regina Kunz^q^, Xin Sun^r^, Norman Buckley^a^, Daniel Sessler^s^, and Gordon H. Guyatt^b^

^a^Anesthesia, McMaster University, Hamilton, Ontario, Canada; ^b^Health Research Methods, Evidence, and Impact, McMaster University, Hamilton, ON, Canada; ^c^Dentistry, Santo Thomas University, Bogota, DC, Colombia; ^d^Pharmaceutical Sciences, University of Sorocaba, Sao Paolo, Sorocaba, Brazil; ^e^Division of Evidence-based Insurance Medicine, University Hospital Basel, Basel, Basel-Stadt, Switzerland; ^f^Medicine, McMaster University, Hamilton, ON, Canada; ^g^Rheumatology, Hospital Aleman, Buenos Aires, Buenos Aires, Argentina; ^h^School of Medicine, University College Dublin, Dublin, Leinster, Ireland; ^i^Anesthesia, University of Toronto, Toronto, ON, Canada; ^j^School of Medicine, University of Toronto, Toronto, ON, Canada; ^k^Accident and Emergency Department, Tseung Kwan O Hospital, Hong Kong, Hong Kong, China; ^l^Accident and Emergency Department, Tuen Mun Hospital, Hong Kong, Hong Kong, China; ^m^Medicine, Innlandet Hospital Trust, University of Oslo, Oslo, Norway; ^n^Faculty of Medicine, Jagiellonian University, Krakow, Lesser Poland Voivodeship, Poland; ^o^Anesthesia, University Hospital Basil, Basel, Basel-Stadt, Switzerland; ^p^Anesthesia and Pain Medicine, Hospital for Sick Children, Toronto, ON, Canada; ^q^Swiss Academy of Insurance Medicine, University Hospital Basel, Basel, Basel-Stadt, Switzerland; ^r^Chinese Evidence-based Medicine CenterCity: Chengdu, Sichuan University, Sichuan, China; ^s^Anesthesiology Outcomes Research, Cleveland Clinic, Cleveland, OH, USA

**CONTACT** Jason W. Busse bussejw@mcmaster.ca

© 2017 Jason W. Busse, Li Wang, Mostafa Kamal el Din, Samantha Craigie, Luis Montoya, Sohail M. Mulla, John J. Riva, Luciane C. Lopes, Nicole Vogel, Eric Chen, Karin Kirmayr, Kyle De Olivera, Lori Oliveri, Alka Kaushal, Luis Chaparro, Inna Oyberman, Arnav Agarwal, Ludwig Tsoi, Tommy Lam, Per Olav Vandvik, Rachel Couban, Sandy Hsu, Malgorzata Bala, Stefan Schandelmaier, Anne Scheidecker, Shanil Ebrahim, Bradley Johnston, Regina Kunz, Xin Sun, Norman Buckley, Daniel Sessler, and Gordon H. Guyatt. Published with license by Taylor & Francis.

This is an Open Access article distributed under the terms of the Creative Commons Attribution License (http://creativecommons.org/licenses/by/3.0/), which permits unrestricted use, distribution, and reproduction in any medium, provided the original work is properly cited. The moral rights of the named author(s) have been asserted.

Introduction/Aim: We conducted a systematic review of randomized controlled trials to establish the benefits and harms of opioids for chronic non-cancer pain (CNCP).

Methods: Studies eligible for our review randomly allocated patients presenting with CNCP to an opioid analgesic or a non-opioid control, and reported outcomes at ≥4 weeks’ follow-up. We identified relevant randomized controlled trials by a systematic search of CINAHL, EMBASE, MEDLINE, AMED, HealthSTAR, PsycINFO, and CENTRAL through to September 2016.

Results: Of 23,040 identified citations, 96 proved eligible. We found evidence of a subgroup effect based on duration of treatment. We found evidence of publication bias for reporting effects on pain and physical function. When restricted to 27 trials that treated patients for ≥3 months and were not at risk of publication bias (13,876 patients), high quality evidence showed that, compared to placebo, opioids reduced pain among patients with CNCP by 0.64cm on a 10cm VAS for pain (95% CI = −0.76 to −0.53, I^2^ = 51%; minimally important difference [MID] is 1cm). When restricted to 33 trials (12,058 patients) that were not at risk of publication bias, high quality evidence showed that, compared to placebo, opioids improved functional outcomes among patients with CNCP by 2.16 points on a 100-point SF-36 physical component summary score (95% CI 1.56 to 2.76, I^2^=55%; MID is 5-points). Low to moderate quality evidence showed no difference in pain relief between opioids and non-steroidal anti-inflammatory drugs (NSAIDs), nabilone, or antidepressants.

Discussion/Conclusions: On average, opioids produce small improvements in pain and function compared to placebo, and are similarly effective for pain relief compared to NSAIDS, nabilone, or anti-depressants.

## Plasma monoamines are potential biomarkers for conditioned pain modulation efficacy

Alisson R. Teles^a^, Diana-Luk Ye^b^, My-Linh Ma^b^, Dee-Anne Naylor^b^, Sheila Bote^b^, Pablo Ingelmo^c^, Serge Marchand^d^, Neil Saran^a^, Jean A. Ouellet^a^, and Catherine E. Ferland^e^

^a^Department of Orthopedics, McGill Scoliosis and Spine Group, McGill University, Montreal, QC, Canada; ^b^McGill Scoliosis and Spine Group Shriners Hospital for Children-Canada, Montreal, QC, Canada; ^c^Department of Anesthesia, Alan Edwards Centre for Research on Pain, McGill University, Montreal, QC, Canada; ^d^Department of Surgery, Université de Sherbrooke, Sherbrooke, QC, Canada; ^e^Department of Orthopedics, Department of Anesthesia, McGill Scoliosis and Spine Group, Alan Edwards Centre for Research on Pain, McGill University, Shriners Hospital for Children-Canada, Montreal, QC, Canada

**CONTACT** Alisson R. Teles alisson.r.teles@gmail.com

© 2017 Alisson R. Teles, Diana-Luk Ye, My-Linh Ma, Dee-Anne Naylor, Sheila Bote, Pablo Ingelmo, Serge Marchand, Neil Saran, Jean A. Ouellet, and Catherine E. Ferland. Published with license by Taylor & Francis.

This is an Open Access article distributed under the terms of the Creative Commons Attribution License (http://creativecommons.org/licenses/by/3.0/), which permits unrestricted use, distribution, and reproduction in any medium, provided the original work is properly cited. The moral rights of the named author(s) have been asserted.

Introduction/Aim: To investigate the role of blood monoamines as biomarkers of conditioned pain modulation (CPM) efficacy.

Methods: 105 pediatric patients with chronic back pain were enrolled in the study. The protocol involved: dosage of plasma monoamines (dopamine, DOPA; serotonin, 5-HT; epinephrine, Epi; norepinephrine, NE; metanephrine, ME; and normetanephrine, NME), quantitative sensory testing and conditioned pain modulation (CPM), as well as clinical assessment (functional disability index, FDI; adolescent pediatric pain tool, APPT; revised children’s anxiety and depression scale, RCADS; Pittsburgh sleep quality index, PSQI).

Results: Plasma levels of monoamines were correlated: DOPA and 5-HT had a positive correlation, and those molecules had negative correlations with Epi, NE, ME, and NME. CPM efficacy presented positive correlations with plasma levels of DOPA (ρ=0.251) and 5-HT (ρ = 0.176) and negative correlations with Epi (ρ = –0.145), NE (ρ = –0.245), ME (ρ = –0.275) and NME (ρ = –0.270). Receiver operating characteristic (ROC) analyses identified cutoffs for 5-HT (<30.0 ng/mL), DOPA (<1.3 pg/mL), ME (≥8.0 pg/mL), NE (≥42.0 pg/mL), and NME (≥42.0 pg/mL) for sub-optimal CPM. CPM efficacy was also associated with autonomic response during the test, measured by variation in the blood pressure. Positive correlation was observed between ME and autonomic response during the test.

Discussion/Conclusions: Plasma monoamines are associated with CPM efficacy. DOPA, 5-HT, NE, NME, and ME can be used as biomarkers of CPM efficacy in pediatrics. Their use as CPM biomarkers is especially important in pediatric patients with neurologic disorders that preclude adequate pain assessment. Future studies are needed to assess the efficacy of tailored treatments for pain according to plasma monoamines.

## Child catastrophizing about parent pain: Potential risk factor for children of parents with chronic pain

Kristen S. Higgins^a^, Christine T. Chambers^b^, Natalie O. Rosen^c^, Simon Sherry^d^, Somayyeh Mohammadi^e^, Mary Lynch^f^, Alexander J. Clark^g^, and Marsha Campbell-Yeo^h^

^a^Psychology & Neuroscience, Dalhousie University, IWK Health Centre, Halifax, Nova Scotia, Canada; ^b^Pediatrics; Psychology & Neuroscience; Anesthesia, Pain Management & Perioperative Medicine; Psychiatry, Dalhousie University, IWK Health Centre, Halifax, Nova Scotia, Canada; ^c^Psychology & Neuroscience; Obstetrics & Gynaecology; Psychiatry, Dalhousie University, IWK Health Centre, Halifax, Nova Scotia, Canada; ^d^Psychology & Neuroscience; Psychiatry, Dalhousie University, Halifax, Nova Scotia, Canada; ^e^Pediatrics, IWK Health Centre, Dalhousie, Halifax, Nova Scotia, Canada; ^f^Anesthesia, Pain Management & Perioperative Care; Pharmacology; Psychiatry, Dalhousie University, QEII Health Sciences Centre, Halifax, Nova Scotia, Canada; ^g^Anesthesia, Pain Management & Perioperative Care, Dalhousie University, QEII Health Sciences Centre, Halifax, Nova Scotia, Canada; ^h^School of Nursing; Psychology & Neuroscience; Pediatrics, Dalhousie University, IWK Health Centre, Halifax, Nova Scotia, Canada

**CONTACT** Kristen S. Higgins Kristen.higgins@dal.ca

© 2017 Kristen S. Higgins, Christine T. Chambers, Natalie O. Rosen, Simon Sherry, Somayyeh Mohammadi, Mary Lynch, Alexander J. Clark, and Marsha Campbell-Yeo. Published with license by Taylor & Francis.

This is an Open Access article distributed under the terms of the Creative Commons Attribution License (http://creativecommons.org/licenses/by/3.0/), which permits unrestricted use, distribution, and reproduction in any medium, provided the original work is properly cited. The moral rights of the named author(s) have been asserted.

Introduction/Aim: Children whose parents have chronic pain are at greater risk for pain and mental health problems than other children (Higgins et al., 2015). Children’s catastrophizing about their pain predicts negative outcomes (Vervoort et al., 2006); catastrophizing about parents’ pain may confer risk through similar mechanisms and be increased in this population due to increased exposure to parental pain. This project examines relationships between child catastrophizing about parents’ pain, child outcomes, and parent pain.

Methods: In an ongoing study, children (8–15 years) of parents with chronic pain (duration at least 6 months) completed a modified Pain Catastrophizing Scale for Parents (PCS-P; Goubert et al., 2006) regarding parents’ pain. Children and parents reported on their pain and catastrophizing about their own pain, and children reported on their internalizing symptoms.

Results: Preliminary analyses included 15 children (mean age = 11.47 years, SD = 2.62, N_females_ = 8) and parents (N_females_ = 12, mean pain intensity = 6.64, SD = 1.91). Child PCS-P scores were associated with catastrophizing about their own pain (r=0.60, p=0.038) and marginally correlated with anxiety (r = 0.57, p = 0.051). Child PCS-P scores were related to parental pain interference (r = 0.66, p = 0.010), but not to parent (r = 0.39, p = 0.187) or child (r = 0.36, p = 0.207) pain intensity or parent pain catastrophizing (r = 0.33, p = 0.248). Rumination about parents’ pain was associated with child internalizing symptoms (r = 0.61, p = 0.036).

Discussion/Conclusions: Results should be interpreted cautiously given the small sample to date; data from the final sample (estimated N = 70) will be reported. Preliminary results suggest that children’s catastrophizing about parents’ chronic pain may be a risk factor for children’s outcomes warranting further study.

## Understanding predictors for prescription opioid consumption 4 months after traumatic injury and corrective surgery

Brittany N. Rosenbloom^a^, Colin J.L. McCartney^b^, Sonya Canzian^c^, Hans J. Kreder^d^, Joel Katz 0000-0002-8686-447X^a^

^a^Psychology, York University, Toronto, ON, Canada; ^b^Anesthesiology and Pain Medicine, University of Ottawa, Ottawa, ON, Canada; ^c^Trauma & Neurosurgery and Mobility Programs, St. Michael’s Hospital, Toronto, ON, Canada; ^d^Orthopedic Surgery, Sunnybrook Health Sciences Centre, Toronto, ON, Canada

**CONTACT** Brittany N. Rosenbloom bnrosen@yorku.ca

© 2017 Brittany N. Rosenbloom, Colin J.L. McCartney, Sonya Canzian, Hans J. Kreder, and Joel Katz. Published with license by Taylor & Francis.

This is an Open Access article distributed under the terms of the Creative Commons Attribution License (http://creativecommons.org/licenses/by/3.0/), which permits unrestricted use, distribution, and reproduction in any medium, provided the original work is properly cited. The moral rights of the named author(s) have been asserted.

Introduction/Aim: Opioids are commonly used for acute pain management after traumatic injury and corrective surgeries. Few studies have prospectively followed patients and their pain management in the months following injury. The aim of this study was to examine the incidence and predictors of persistent prescription opioid use 4-months after traumatic injury.

Methods: Adults who sustained a traumatic musculoskeletal injury were recruited to participate in this observational prospective, longitudinal study within 14 days of injury (T1) and followed for 4-months (T2). Measures included questionnaires on pain, opioid consumption, pain disability, anxiety, depression, and posttraumatic stress symptoms as well as a chart review for injury related information. We used multivariate logistic regression models to progressively add significant T1 and T2 variables in order to assess their relative contributions to explaining T2 opioid use.

Results: The sample consisted of 122 patients [66.4% male; mean age=44.8 years (SD = 17.1)]. While in hospital, 94/3% (n = 115) were taking prescription opioids. At T2, 35.3% (n = 43) patients reported using prescription opioids. After controlling for age, sex, injury severity, T1 pain severity, and T2 symptoms of depression, two factors were significantly related to T2 prescription opioid use; namely, T2 pain severity (OR = 1.366 95% CI 1.071, 1.742) and T2 pain self-efficacy (OR = .943 95% CI .903, .984).

Conclusions: Over a third of adults were using opioids 4 months after traumatic musculoskeletal injury. Their use is related to pain severity and how well patients cope specifically with their pain, over and above other psychological factors, such as depression and anxiety.

## Impact of pain and other adverse outcomes on healthcare use following ambulatory surgery in Ontario

Monakshi Sawhney^a^, Elizabeth VanDenKerkhof^b^, Farzana Haq^c^, David Goldstein^d^, and Louie Wang^e^

^a^School of Nursing, Faculty of Health Sciences Kingston, Queen’s University Ontario Canada; ^b^Department of Anesthesiology & Perioperative Medicine, and School of Nursing, Faculty of Health Sciences,Queen’s University, Kingston Ontario Canada; ^c^ICES Queen’s, ICES Queen’s Kingston Ontario Canada; ^d^Anesthesiology, Brockville General Hospital Brockville Ontario Canada; ^e^Department of Anesthesiology & Perioperative Medicine, Kingston General Hospital, Queen’s University Brockville Ontario Canada

**CONTACT** Monakshi Sawhney mona.sawhney@queensu.ca

© 2017 Monakshi Sawhney, VanDenKerkhof Elizabeth, Farzana Haq, David Goldstein, Louie Wang. Published with license by Taylor & Francis

This is an Open Access article distributed under the terms of the Creative Commons Attribution License (http://creativecommons.org/licenses/by/3.0/), which permits unrestricted use, distribution, and reproduction in any medium, provided the original work is properly cited. The moral rights of the named author(s) have been asserted.

Introduction/Aim: The purpose of this study was to describe healthcare use (HCU) in the first 3 days following ambulatory surgery in Ontario. The objectives were to measure emergency service utilization (ED) and admission (AD) rates, identify the primary reasons for accessing services, and identify groups at high risk for HCU.

Methods: A prospective cohort study using administrative data from the Institute for Clinical Evaluative Sciences was conducted in adults who underwent ambulatory surgery between 2009-2014.

Results: 1,062,833 adults (52% female) with a mean age of 55 years were included. The most common procedures were colonoscopy (35.0%), rectal surgery (13.2%), partial hysterectomy (11.1%), cholecystectomy (9.5%) and knee repair (9.5%). Overall, 2.5% (n=26,496) returned to ED and 2.0% (21,086) were admitted. Thirty-one percent of ED visits occurred on postoperative day 1 (POD1) and 27.6% on POD2, with 79.7% of admissions on POD0. The highest rate of return to ED was after tonsillectomy (8.1%), cholecystectomy (5.1%) and appendectomy (5.0%). The highest rates of admission were for appendectomy (24.2%), muscle repair of chest or abdomen (non-hernia) (9.7%) and cholecystectomy (7.2%). Top reasons for ED visits were bleeding (12.8%) and pain (12.5%); admissions were primarily for convalescence (42.2%). Acute pain was the top reason for HCU after cholecystectomy (20.4% of visits), tonsillectomy (24.1%), shoulder surgery (28.9%) and the second most common reason after hernia (10.6%) and knee surgery (15.9%).

Discussion/Conclusions: Programs aimed at managing postoperative pain in high risk groups would likely cost less than unplanned healthcare utilization and would improve outcomes and healthcare quality.

## Clinical assessment of mechanical and thermal sensitivity of adolescents with painful scoliosis

My-Linh Ma^a^, Dee-Anne Naylor^b^, Jasmine Chong^c^, Carina D’Aiuto^d^, Pablo Ingelmo^e^, Neil Saran^f^, Serge Marchand^g^, Jean A. Ouellet^b^, and Catherine E. Ferland^e^

^a^Experimental Surgery, McGill University, Montreal, Quebec, Canada; ^b^Orthopaedic Surgery, McGill University Health Centre, Montreal, Quebec, Canada; ^c^Parasitology, McGill University, Montreal, Quebec, Canada; ^d^Pharmacology, McGill University, Montreal, Quebec, Canada; ^e^Anesthesia, McGill University Health Centre, Montreal, Quebec, Canada; ^f^Pediatric surgery, McGill University Health Centre, Montreal, Quebec, Canada; ^g^Surgery, Université de Sherbrooke, Sherbrooke, Quebec, Canada

**CONTACT** My-Linh Ma my-linh.ma@mail.mcgill.ca

© 2017 My-Linh Ma, Dee-Anne Naylor, Jasmine Chong, Carina D’Aiuto, Pablo Ingelmo, Neil Saran, Serge Marchand, Jean A. Ouellet, and Catherine E. Ferland. Published with license by Taylor & Francis.

This is an Open Access article distributed under the terms of the Creative Commons Attribution License (http://creativecommons.org/licenses/by/3.0/), which permits unrestricted use, distribution, and reproduction in any medium, provided the original work is properly cited. The moral rights of the named author(s) have been asserted.

Introduction/Aim: A significant proportion of adolescent idiopathic scoliosis (AIS) patients have chronic back pain. It was demonstrated that a suboptimal conditioned pain modulation (CPM) efficacy is associated with chronic pain. The objective was to investigate if chronic pain patients with an optimal CPM efficacy display different alterations in their ascending sensory pain processes compared to chronic pain patients with a suboptimal CPM efficacy.

Methods: Sixty patients with painful AIS performed mechanical and thermal quantitative sensory testing (QST). Patients were stratified into 2 groups based on the efficacy of their inhibitory pain response (optimal and suboptimal groups, n = 30 each). Differences in the mechanical detection, pressure pain, heat pain and tolerance thresholds and thermal temporal pain summation) between the 2 groups were evaluated using Mann-Whitney tests. Pain, functional and emotional profiles were assessed with questionnaires.

Results: Great variability in the CPM response was observed among the cohort (mean of -32.44%, 95%CI -45.16 to -18.71). A significant difference was observed in the heat pain threshold between the 2 groups (mean of 40.20°C ±3.25 and 38.56°C ±3.30 respectively, (p = 0.0276)), but not in the other QST parameters (p > 0.05). More patients had functional disability in the optimal CPM group (30% *versus* 10% in the suboptimal group), although the 2 groups displayed similar pain and emotional profiles.

Discussion/Conclusions: Our results suggest that AIS patients with a suboptimal endogenous inhibitory pain response develop an increased peripheral sensitivity to heat. Further work is needed to understand the underlying cause of chronic back pain associated to scoliosis.

## The effects of rhythmic sensory stimulation on fibromyalgia symptoms: A randomized control trial

T. Braun Janzen^a^, Denise Paneduro^b^, Kethmini Amarasinghe^b^, Sally Chung^b^, Larry Picard^b^, Allan Gordon^b^, and Lee Bartel^a^

^a^Faculty of Music, Music and Health Research Collaboratory, University of Toronto, Toronto, ON, Canada; ^b^Wasser Pain Management Centre, Sinai Health System, Toronto, ON, Canada

**CONTACT** Thenille Braun Janzen thenille.braunjanzen@utoronto.ca

© 2017 Thenille Braun Janzen, Denise Paneduro, Kethmini Amarasinghe, Sally Chung, Larry Picard, Allan Gordon, and Lee Bartel. Published with license by Taylor & Francis.

This is an Open Access article distributed under the terms of the Creative Commons Attribution License (http://creativecommons.org/licenses/by/3.0/), which permits unrestricted use, distribution, and reproduction in any medium, provided the original work is properly cited. The moral rights of the named author(s) have been asserted.

Introduction/Aim: This double-blinded exploratory randomized control trial evaluated the effects of rhythmic sensory stimulation with gamma frequency vibrotactile and auditory stimuli on fibromyalgia symptoms.

Methods: Fifty fibromyalgia patients were randomly assigned to two groups: *1)* continuous sine wave single-frequency stimulation (40 Hz) and *2)* random intermittent complex wave gamma range stimulation with peaks at 45 and 95 Hz. The intervention was self-administered with a portable vibroacoustic therapy device 30 minutes daily, 5 days per week, over 5 weeks, concomitant with standard care. Fibromyalgia symptoms (Fibromyalgia Impact Questionnaire Revised - FIQR), pain severity (Brief Pain Inventory), depression severity (Patient Health Questionnaire - 9), quality of life (Quality of Life Enjoyment and Satisfaction Questionnaire), and sleep quality (Pittsburg Sleep Quality Index) were assessed at baseline and post-intervention. Data were analyzed by intention to treat.

Results: Results revealed statistically significant improvements between baseline and post-intervention assessments in all outcome measures, including fibromyalgia symptoms, pain severity and interference, depression severity, quality of life, and sleep quality. No significant group differences were detected. Of those who completed all study assessments (*n* = 38), clinical significant changes in the FIQR post-intervention scores were observed for 20 patients, with an average improvement of 40% (range: 14.16% to 90%).

Discussion/Conclusions: These findings suggest that rhythmic sensory stimulation with gamma frequency vibrotactile and auditory stimuli can potentially decrease pain severity and fibromyalgia symptoms, ease associated comorbidities such as sleep disturbances and depression, and improve patients’ quality of life. Further research is needed to understand the mechanisms underlying these effects.

## Measuring changes from admission to discharge in an interdisciplinary chronic pain management program: Reduction of co-morbidity

Yuelin Li^a^, and Eleni G. Hapidou^b^

^a^Psychology Neuroscience and Behavior & Biology, McMaster University, Hamilton, ON, Canada; ^b^Psychiatry and Behavioral Neurosciences & Michael G. DeGroote Pain Clinic, McMaster University and Hamilton Health Sciences, Hamilton, ON, Canada

**CONTACT** Yuelin Li liyl2@mcmaster.ca

© 2017 Yuelin Li and Eleni G. Hapidou. Published with license by Taylor & Francis.

This is an Open Access article distributed under the terms of the Creative Commons Attribution License (http://creativecommons.org/licenses/by/3.0/), which permits unrestricted use, distribution, and reproduction in any medium, provided the original work is properly cited. The moral rights of the named author(s) have been asserted.

Introduction/Aim: Co-morbidity between psychiatric disorders such as depression and anxiety is very common in chronic pain. Most patients admitted to interdisciplinary chronic pain programs receive diagnoses of clinical depression and anxiety in addition to their chronic pain. They also tend to provide high ratings on psychometric measures of depression and anxiety. The purpose of this study was to examine the value of a four-week intensive interdisciplinary program in reducing this co-morbidity.

Methods: Differences between admission and discharge from a four-week program on several measures of pain-related variables such as depression, anxiety, pain intensity, pain-related disability, catastrophizing and other pain-related variables were examined in 602 consecutively admitted patients with chronic pain (52% women). A MANOVA also examined these differences in males and females.

Results: Highly significant differences were obtained between admission and discharge on all variables examined (p < 0.001). Gender effects were not obtained in terms of the co-morbidity variables. No interaction effects were found. Of particular interest here are the reductions in depression, catastrophizing (both at 25%) and anxiety (13%) scores at discharge, all clinically significant results.

Discussion/Conclusions: These results supported the hypothesis that co-morbidity of pain and psychological distress in individuals with chronic pain can be reduced after four weeks of interdisciplinary pain management.

## Cardiac pain in women: An iterative process to mapping the evidence

Monica Parry^a^, Ann Kristin Bjørnnes^a^, Hance Clarke^b^, Cooper L. Canadian^c^, Allan Gordon^d^, Paula Harvey^e^, Chitra Lalloo^f^, Marit Leegaard^g^, Sandra LeFort^h^, J. McFetridge-Durdle^i^, Michael McGillion^j^, Sheila O’Keefe-McCarthy^k^, Jennifer Price^e^, Jennifer Stinson^f^, Charles Victor^l^, and Judy Watt-Watson^m^

^a^Lawrence S. Bloomberg Faculty of Nursing, University of Toronto, Toronto, ON, Canada; ^b^Pain Research Unit, Toronto General Hospital, Toronto, ON, Canada, ON, Canada; ^c^Pain Coalition (CPC), Toronto, ON, Canada; ^d^Wasser Pain Management Centre, Mount Sinai Hospital, Toronto, ON, Canada; ^e^Women’s College Hospital, Toronto, ON, Canada; ^f^The Hospital for Sick Children, Toronto, ON, Canada; ^g^Nursing, Oslo and Akershus University College of Applied Sciences, Oslo, Norway; ^h^Memorial University of Newfoundland, St. John’s, NL, Canada; ^i^Florida State University, Tallahasse, Florida, United States; ^j^McMaster University, Hamilton, ON, Canada; ^k^Brock University, St, Catherines, ON, Canada; ^l^Health Policy, Management and Evaluation, University of Toronto, Toronto, ON, Canada; ^m^University of Toronto, Toronto, ON, Canada

**CONTACT** Monica Parry Monica.parry@utoronto.ca

© 2017 Monica Parry, Ann Kristin Bjørnnes, Hance Clarke, Cooper L. Canadian, Allan Gordon, Paula Harvey, Chitra Lalloo, Marit Leegaard, Sandra LeFort, Judy McFetridge-Durdle, Michael McGillion, Sheila O’Keefe-McCarthy, Jennifer Price, Jennifer Stinson, Charles Victor, and Judy Watt-Watson. Published with license by Taylor & Francis.

This is an Open Access article distributed under the terms of the Creative Commons Attribution License (http://creativecommons.org/licenses/by/3.0/), which permits unrestricted use, distribution, and reproduction in any medium, provided the original work is properly cited. The moral rights of the named author(s) have been asserted.

Introduction/Aim: To establish the current state of research related to the self-management of cardiac pain in women using the process and methodology of evidence mapping.

Methods: The main purpose of evidence mapping is to provide an overview of a broad range of research and identify evidence gaps and future research needs. Six steps were used to construct an evidence map of cardiac pain in women: 1) Identify the scope of the evidence map, 2) Define the key variables, 3) Establish a comprehensive search strategy, 4) Identify study inclusion and exclusion criteria, 5) Systematically retrieve, screen and classify the evidence, and 6) Report the findings in an evidence map. Twenty-one databases and grey literature sources were systematically searched using keywords and Medical Subject Headings (MeSH).

Results: In total, 6,582 eligible citations were identified. After exclusions, 288 unique articles were included in the evidence map: 24% (n = 68) reviews, 17% (n = 49) intervention studies, and 59% (n = 168) non-intervention studies. Seventy percent (n = 200) of the citations represented obstructive coronary artery disease (CAD), 5% (n = 14) non-obstructive CAD, and 14% (n = 41) post-procedural pain. The median sample size across the primary research studies was 90 and the mean age was 63 years.

Discussion/Conclusions: Our evidence map suggests that while much is known about the differing presentations of obstructive cardiac pain in middle-aged women, little research has focused on young and old women, non-obstructive cardiac pain, or self-management interventions to assist women to manage cardiac pain.

## Preliminary acceptability and feasibility of virtual reality distraction for subcutaneous port access in youth with cancer

Kathryn A. Birnie^a^, Lindsay Jibb^b^, Oussama Abla^c^, Karyn Positano^d^, Vanessa Hum^e^, Petra Hroch^f^, Nabilah Juma^g^, and Jennifer Stinson^a^

^a^Lawrence S. Bloomberg Faculty of Nursing, University of Toronto/Child Health Evaluative Sciences, University of Toronto/Hospital for Sick Children, Toronto, Ontario, Canada; ^b^Nursing, University of Ottawa, Ottawa, Ontario, Canada; ^c^Oncology/Haematology, Hospital for Sick Children, Toronto, Ontario, Canada; ^d^Child Life, Hospital for Sick Children, Toronto, Ontario, Canada; ^e^Child Health Evaluative Sciences, Hospital for Sick Children, Toronto, Ontario, Canada; ^f^Michael G. DeGroote School of Medicine, McMaster University, Hamilton, Ontario, Canada; ^g^Neonatal Intensive Care Unit, Hospital for Sick Children, Toronto, Ontario, Canada

**CONTACT** Kathryn A. Birnie kathryn.birnie@sickkids.ca

© 2017 Kathryn A. Birnie, Lindsay Jibb, Oussama Abla, Karyn Positano, Vanessa Hum, Petra Hroch, Nabilah Juma, and Jennifer Stinson. Published with license by Taylor & Francis.

This is an Open Access article distributed under the terms of the Creative Commons Attribution License (http://creativecommons.org/licenses/by/3.0/), which permits unrestricted use, distribution, and reproduction in any medium, provided the original work is properly cited. The moral rights of the named author(s) have been asserted.

Introduction/Aim: Children with cancer cite needle procedures, such as subcutaneous port access (SCP), as the most painful and distressing experiences during treatment. Distraction via virtual reality (VR) offers promise for reducing needle-related pain and distress given its highly immersive and interactive virtual environment. Prior to assessing efficacy, the VR intervention must be deemed acceptable and safe for youth with cancer during SCP. Specific aims were to assess (1) acceptability of VR (hardware and software) in youth with cancer, (2) any adverse events with VR use, and (3) capacity to communicate with youth during VR.

Methods: A mixed-methods, child-centered approach with three iterative cycles of VR intervention testing. Testing cycles included observation and interviews with youth with cancer 8–18 years old using VR hardware and software equipment to identify acceptability and safety issues requiring intervention refinement.

Results: Eight youth with cancer tested the VR interactive underwater distraction intervention (5 prior to and 3 during SCP). Youth reported the VR easy to navigate. They desired more interactive intervention components, a greater variety of games and music, and optional notifications of steps during needle procedure. Limitations to engaging fully with the VR equipment (e.g., turning head left/right) were identified due to requirements to remain still during the procedure. No adverse events occurred.

Discussion/Conclusions: Next steps include completing iterative testing during SCP procedures with modifications based on user feedback, and a pilot randomized controlled trial to evaluate preliminary effectiveness of VR distraction on pain intensity and distress during SCP needle insertion in this population.

## Pain as a key dimension of complexity: Physician narratives of patients with chronic pain

Fiona Webster^a^, Kathleen Rice^a^, Onil Bhattacharyya^b^, Joel Katz^c^, and Ross Upshur^d^

^a^Institute of Health Policy Management and Evaluation, Dalla Lana School of Public Health, University of Toronto; ^b^Women’s College Research Institute, Toronto, Ontario, Canada; ^c^Department of Psychology, York University; ^d^Dalla Lana School of Public Health, University of Toronto

**CONTACT** Fiona Webster fiona.webster@utoronto.ca

© 2017 Fiona Webster, Kathleen Rice, Onil Bhattacharyya, Joel Katz, and Ross Upshur. Published with license by Taylor & Francis.

This is an Open Access article distributed under the terms of the Creative Commons Attribution License (http://creativecommons.org/licenses/by/3.0/), which permits unrestricted use, distribution, and reproduction in any medium, provided the original work is properly cited. The moral rights of the named author(s) have been asserted.

Background: While there has been a great deal of attention paid to identifying the epidemiology of multi-morbidity and patient complexity using administrative data, comparatively little attention has been paid to the processes of care that treating complex patients entails, and to identifying what high-quality, patient-centered care for these patients should look like. Consequently, the concept of patient complexity itself does not necessarily speak to how challenging or straightforward it may be to provide high-quality patient-centered care. Further, the phenomenon of multi-morbidity is not well understood and currently includes 16 common medical conditions, not including pain. Methods: Our team undertook a critical ethnographic approach known as institutional ethnography in the province of Ontario, Canada. Results: We interviewed over 40 participants, including primary care providers (physicians, nurses, nurse practitioners, and allied health professionals).

Discussion: Our findings suggest that a definition of patient complexity based solely on presence and number of multi-morbidities is far too narrow. Indeed, from the perspective of primary care physicians, patients they consider complex are challenging not so much due their medical problems alone, but rather to their social and living conditions. In virtually every example that we were offered, chronic pain was raised by physicians as representing the greatest challenge to care provision. They also described that pain was frequently bound up with poverty, trauma, and mental health concerns. Such patients were challenging and frustrating for health care providers in part because the interventions needed are far beyond the scope of expertise, even as their social issues rendered the treatment of potentially-straightforward medical problems complicated.

## Chronic pelvic pain: A patient perspective of their first health care provider experience

Maram AlShareef, Denise Paneduro, Kareena Gurbaxani, Kethmini Amarasinghe, Leah Pink, and Allan Gordon

Wasser Pain Management Centre, Sinai Health System, Toronto, ON, Canada

**CONTACT** Maram AlShareef Malshareef81@yahoo.com

© 2017 Maram AlShareef, Denise Paneduro, Kareena Gurbaxani, Kethmini Amarasinghe, Leah Pink, and Allan Gordon. Published with license by Taylor & Francis.

This is an Open Access article distributed under the terms of the Creative Commons Attribution License (http://creativecommons.org/licenses/by/3.0/), which permits unrestricted use, distribution, and reproduction in any medium, provided the original work is properly cited. The moral rights of the named author(s) have been asserted.

Introduction/Aim: Chronic pelvic pain (CPP) is a common condition observed in primary health care yet no research has surveyed the experience of these individuals within the health system. In order to address this gap, the current study explored patients’ experiences when consulting for the first time with a health care provider (HCP) to communicate symptoms of CPP.

Methods: Thirty individuals (*M* age = 40, *SD* = 12; 27 females) with CPP completed a 10–15 minute anonymous survey while awaiting their medical appointment at the Wasser Pain Management Centre. Participants were asked to provide demographic information, pain duration, pain onset, the medical specialty of their initial HCP, and to report their feelings associated with expressing their symptoms as well as their overall satisfaction.

Results: The average pain duration was 5 years and 70% of patients were unsure how their pain began. The majority of patients indicated their first HCP was a family physician or gynecologist. Sixty percent reported a negative experience while expressing their symptoms such as feeling uncomfortable and hesitant and 67% reported feeling disappointed with the quality of health care obtained from their initial HCP. Patients were most disappointed with the level of dismissiveness and the lack of knowledge and personalized care received. The average overall satisfaction reported was 3.93 out of 10.

Discussion/Conclusions: These findings highlight the urgent need to provide early education regarding adequate diagnostic approaches for HCPs. Adopting a supportive and empathic therapeutic environment is critical for facilitating optimal health care for individuals with CPP.

## Physical and psychological interventions for pediatric cancer pain management

Perri R. Tutelman^a^, Christine T. Chambers^b^, Jennifer N. Stinson^c^, Jennifer A. Parker^d^, Melanie Barwick^e^, Fiona Campbell^f^, Conrad V. Fernandez^g^, Karen Irwin^h^, Lindsay A. Jibb^i^, Paul C. Nathan^j^, and Holly O. Witteman^k^

^a^Department of Psychology and Neuroscience/Centre for Pediatric Pain Research, Dalhousie University/IWK Health Centre, Halifax, Nova Scotia, Canada; ^b^Department of Pediatrics, Department of Psychology and Neuroscience/Centre for Pediatric Pain Research, Dalhousie University/IWK Health Centre, Halifax, Nova Scotia, Canada; ^c^Faculty of Nursing, Faculty of Medicine/Department of Child Health Evaluative Sciences; Department of Anesthesia and Pain Medicine, University of Toronto/Hospital for Sick Children, Toronto, Ontario, Canada; ^d^Centre for Pediatric Pain Research, IWK Health Centre, Halifax, Nova Scotia, Canada; ^e^Department of Psychiatry, Dalla Lana School of Public Health/Child and Youth Metal Health Research Unit, Department of Child Health Evaluative Sciences, University of Toronto/Hospital for Sick Children, Toronto, Ontario, Canada; ^f^Department of Anesthesia/Department of Anesthesia and Pain Medicine, University of Toronto/Hospital for Sick Children, Toronto, Ontario, Canada; ^g^Department of Pediatrics/Division of Haematology/Oncology, Dalhousie University/IWK Health Centre, Halifax, Nova Scotia, Canada; ^h^Cancer Knowledge Network, Toronto, Ontario, Canada; ^i^Faculty of Nursing/Department of Child Health Evaluative Sciences, Department of Haematology/Oncology, University of Toronto/Hospital for Sick Children, Toronto, Ontario, Canada; ^j^Faculty of Medicine, Institute of Health Policy Management and Evaluation/Department of Child Health Evaluative Sciences, Department of Haematology/Oncology, University of Toronto/Hospital for Sick Children, Toronto, Ontario, Canada; ^k^Department of Family and Emergency Medicine/Population Health and Optimal Health Practices Research Unit, Université Laval/Research Centre of the CHU de Québec-Université Laval, Québec City, Québec, Canada

**CONTACT** Perri R. Tutelman ptutelman@dal.ca

© 2017 Perri R. Tutelman, Christine T. Chambers, Jennifer N. Stinson, Jennifer A. Parker, Melanie Barwick, Fiona Campbell, Conrad V. Fernandez, Karen Irwin, Lindsay A. Jibb, Paul C. Nathan, and Holly O. Witteman. Published with license by Taylor & Francis.

This is an Open Access article distributed under the terms of the Creative Commons Attribution License (http://creativecommons.org/licenses/by/3.0/), which permits unrestricted use, distribution, and reproduction in any medium, provided the original work is properly cited. The moral rights of the named author(s) have been asserted.

Introduction/Aim: Prior evidence supports the use of various interventions to manage pain in children with cancer including pharmacological, physical and psychological approaches. Physical and psychological interventions represent important components of multimodal pain management, yet, research on their use in pediatric oncology populations is scarce. Moreover, the classification of individual interventions as either physical or psychological remains uncertain. Thus, the purpose of this study was to examine patterns of physical and psychological pain intervention use by parents of children with cancer.

Methods: Participants included an international sample of 230 parents (89% mothers) of children (50% boys) ages 1-18 years (mean = 8.9, *SD* = 4.5) with cancer. Parents completed an online questionnaire about their child’s pain in the past 2 weeks, including questions about how often they use 10 different physical and psychological pain interventions rated on a 5-point scale.

Results: Almost all parents (88%) reported administering at least one intervention within the past 2 weeks. An exploratory factor analysis on parents’ responses revealed a 2-factor structure characterizing the strategies as either physical (α = 0.57) or psychological (α = 0.79). Heat, cold, and massage/rubbing were characterized as physical while talking, deep breathing, relaxation, rest/sleep, distraction, prayer/meditation and imagery were characterized as psychological. Responses for both factors were positively associated with parent-reported child worst, least, and average pain intensity.

Discussion/Conclusions: Parents reported frequent pain intervention use and their responses categorized strategies as either physical or psychological. Future research will examine parental use of multimodal interventions and decision making surrounding selection of pain interventions.

## Risk mitigation strategies for opioid prescribing in chronic non-cancer pain: A systematic review

Raad Yameen^a^, Anna Goshua^b^, Samantha Craigie^c^, Rachel Couban^d^, Patrick Jiho Hong^e^, Curtis May^f^, Regina Li^g^, Li Wang^h^, and Jason Busse^i^

^a^Institute of Medical Sciences, University of Toronto, Toronto, Ontario, Canada; ^b^Health Sciences, McMaster University, Hamilton, Ontario, Canada; ^c^Anesthesia, McMaster University, Hamilton, Ontario, Canada; ^d^Anesthesia, McMaster University, Hamilton, Ontario, Canada; ^e^Faculty of Medicine, University of Ottawa, Ottawa, Ontario, Canada; ^f^Faculty of Medicine, University of British Columbia, Vancouver, British Columbia, Canada; ^g^Health Sciences, McMaster University, Hamilton, Ontario, Canada; ^h^Anesthesia, McMaster University, Hamilton, Ontario, Canada; ^i^Michael G. DeGroote Institute for Pain Research and Care, McMaster University, Hamilton, Ontario, Canada

**CONTACT** Raad Yameen raad.yameen@mail.utoronto.ca

© 2017 Raad Yameen, Anna Goshua, Samantha Craigie, Rachel Couban, Pat Hong, Curtis May, Regina Li, Li Wang, Jason Busse. Published with license by Taylor & Francis.

This is an Open Access article distributed under the terms of the Creative Commons Attribution License (http://creativecommons.org/licenses/by/3.0/), which permits unrestricted use, distribution, and reproduction in any medium, provided the original work is properly cited. The moral rights of the named author(s) have been asserted.

Introduction/Aim: Prevention of harm is an important concern when prescribing opioids for chronic non-cancer pain (CNCP). We conducted a systematic review on risk mitigation strategies for opioid prescribing in CNCP patients.

Methods: We searched MEDLINE, PubMed, EMBASE, CINAHL, PsycINFO through July 2016 for randomized controlled trials (RCTs) and observational studies investigating the effect of risk mitigation strategies (e.g. urine drug screening, treatment agreements, take-home naloxone, abuse-deterrent formulations, patch exchange, structured opioid therapy, specialist review, screening for aberrant drug-related behaviours) for CNCP patients prescribed opioids. Screening, data abstraction and risk of bias assessment were performed independently and in duplicate. When possible, we pooled relative measures of association as pooled odds ratios (OR).

Results: We identified 8 observational studies that were eligible for our review, all of which provided low quality evidence. Four articles (2,624 patients) suggested no association between treatment agreements and opioid misuse (pooled OR 1.28, 95% CI 0.80 to 2.05), one study (179,385 patients) found no association between baseline urine drug screening and risk of opioid overdose (HR 1.36, 95% CI 0.79-2.34), one study (1,985 patients) reported no association between take-home naloxone and the odds of fatal overdose (OR 1.08, 95%CI 0.18-6.46) and two industry-funded, single-arm observational studies reported a lower incidence of addiction, overdose, and death after tamper-resistant OxyContin was introduced.

Discussion/Conclusions: Although guidelines and professional organizations often recommend risk mitigation strategies to reduce harm when prescribing opioids for CNCP, the available evidence provides very little support for any approach.

## Development and content validity testing of the Indigenous Pain Expression Assessment Tool

Warda Limaye^a^, Margot Latimer^b^, and Amy Bombay^c^

^a^Department of Medicine, Dalhousie University, Halifax, NS, Canada; ^b^Faculty of Health Professions, Dalhousie University, IWK Health Centre, Halifax, NS, Canada; ^c^Department of Psychiatry, Department of Nursing, Dalhousie University, Halifax, NS, Canada

**CONTACT** Warda Limaye Warda.Limaye@dal.ca

© 2017 Warda Limaye, Margot Latimer, and Amy Bombay. Published with license by Taylor & Francis.

This is an Open Access article distributed under the terms of the Creative Commons Attribution License (http://creativecommons.org/licenses/by/3.0/), which permits unrestricted use, distribution, and reproduction in any medium, provided the original work is properly cited. The moral rights of the named author(s) have been asserted.

Introduction/Aim: Rates of pain conditions are disproportionately high amongst Indigenous populations in Canada. Research has shown that healthcare provider pain assessments in non-Caucasian populations are lower than those conducted for non-Hispanic Caucasians, leading to disparities in effective pain treatment. As few studies examine this relationship between healthcare providers and Canadian Indigenous groups, the aim of this study is to develop text-based vignettes to further the study of pain assessments for Aboriginal patients by healthcare providers.

Methods: Evidence-based, patient-related variables (sex, pain type, age, and ethnic saliency) were included in text-based vignettes with the objective of measuring responder assessment of race sensitive pain expression or beliefs. The vignettes were provided to 7 content experts in pain management to score content validity on three dimensions: usability, comprehensibility, and scenario validity. The results of the CVI (Lynn, 1986), including revisions and content expert suggestions for improvements, will be presented. The revised tool will be piloted in a survey-based, quantitative study with medical students.

Results: 4 responses were received, including from 1 Indigenous content expert. Average ratings were 2.5/4 for usability, 3.5/4 for comprehensibility, and 3.25/4, 2.75/4, and 3.5/4 for scenario validity, with no rating lower than 2/4. Feedback containing recommendations was received.

Discussion/Conclusions: The tool was rated to be comprehensible and relevant, with clear semantics. Recommendations included increasing the amount of clinically relevant details to aid in uncovering provider attitudes. The revised vignettes, containing expert feedback, will proceed to the pilot stage and begin to contribute to this understudied area of clinical research.

### Acknowledgments

The authors gratefully acknowledge conference travel support by the JJ Carroll Travel Fund.

## How much pain for your gain? Psychophysical evaluation of the monetary value of pain

Hocine Slimani^a^, Pierre Rainville^b^, and Mathieu Roy^a^

^a^Psychology, McGill University, Montreal, Quebec, Canada; ^b^Centre de Recherhe de l’Institut de Gériatrie de l’Université de Montréal, Université de Montréal, Montreal, Quebec, Canada

**CONTACT** Hocine Slimani ho.slimani@gmail.com

© 2017 Hocine Slimani, Pierre Rainville, and Mathieu Roy. Published with license by Taylor & Francis

This is an Open Access article distributed under the terms of the Creative Commons Attribution License (http://creativecommons.org/licenses/by/3.0/), which permits unrestricted use, distribution, and reproduction in any medium, provided the original work is properly cited. The moral rights of the named author(s) have been asserted.

Introduction/Aim: In order to make optimal decisions between goods of different nature, instrumental decision-making systems must base their choices on an abstract quantity: *value* (equivalent to the concept of *utility* in economy). In the present study, we aimed at determining the monetary value of pain in order to gain insight on how it influences reward seeking.

Methods: 30 healthy volunteers were recruited. Painful stimuli consisted of electrical shocks (Digitimer) delivered on the ankle. We first determined the psychometric function of their pain sensitivity. Thereafter, participants underwent a decision-making task during which they had to accept or decline offers that included pairs of varying levels of pain (threshold to tolerance) and monetary compensations (0 to 5 $).

Results: Our data show that the monetary value of pain increases as a function of stimulus intensity (t = 2.63, p = 0.001), with steeper increments in pain value when approaching pain tolerance.

Discussion/Conclusions: Our findings indicate that similar increases in perceived pain intensity yield greater gains in value when approaching pain tolerance than near-threshold levels.

## Effectiveness of breastfeeding or expressed breast milk to reduce procedural pain response in full term and preterm infants: A systematic review

Britney Benoit^a,^^b,^^c^, Ruth Martin-Misener^a^, Margot Latimer^a,^^b^, and Marsha Campbell-Yeo^a,^^b,^^c,^^d,^^e^

^a^School of Nursing, Dalhousie University, Nova Scotia, Canada; ^b^Centre for Pediatric Pain Research, IWK Health Centre, Nova Scotia, Canada; ^c^Maternal Newborn Program, IWK Health Centre, Nova Scotia, Canada; ^d^Department of Psychology and Neuroscience, Dalhousie University, Nova Scotia, Canada; ^e^Department of Pediatrics, IWK Health Centre, Nova Scotia, Canada

**CONTACT** Britney Benoit Britney.Benoit@Dal.Ca

© 2017 Britney Benoit, Ruth Martin-Misener, Margot Latimer, and Marsha Campbell-Yeo. Published with license by Taylor & Francis.

This is an Open Access article distributed under the terms of the Creative Commons Attribution License (http://creativecommons.org/licenses/by/3.0/), which permits unrestricted use, distribution, and reproduction in any medium, provided the original work is properly cited. The moral rights of the named author(s) have been asserted.

Aim: To provide an updated synthesis of the current state of evidence for the effectiveness of breastfeeding and expressed breast milk feeding in reducing procedural pain in full term and preterm infants.

Methods: A systematic search of key electronic databases (PubMed, CINAHL, EMBASE) was completed from January 1, 2011 to December 22, 2016. The search strategy included key terms for infant, breastfeeding, breast milk, and pain. Inclusion criteria required that studies be 1) an empirical investigation examining the use of breastfeeding or expressed breast milk as a pain relieving intervention, 2) include a sample of full term or preterm born infants, and 3) be published in English in a peer-reviewed journal. Risk of bias was scored using using Cochrane tools.

Results: Of the 1,032 abstracts screened, 21 were found eligible for inclusion. Fifteen studies reported on the use of breastfeeding or expressed breast milk in full term infants (*n* = 1,908) and six reported on preterm infants (*n* = 428). Direct breastfeeding was more effective than maternal holding, maternal skin-to-skin contact, topical anesthetics, and music therapy; and was as or more effective than sweet tasting solutions in full term infants. Expressed breast milk was not consistently found to reduce pain responding in full term or preterm infants. Studies generally had moderate to high risk of bias.

Conclusion: There is sufficient evidence to recommend direct breastfeeding for procedural pain management in full term infants. Based on current evidence, expressed breast milk alone should not be considered an adequate intervention.

## Prediction of outcome in an interdisciplinary chronic pain management program

E. G. Hapidou^a^, and Yuelin Li^b^

^a^Psychiatry and Behavioral Neurosciences & Michael G. DeGroote Pain Clinic, McMaster University and Hamilton Health Sciences, Hamilton, ON, Canada; ^b^Psychology Neuroscience and Behavior & Biology, McMaster University, Hamilton, ON, Canada

**CONTACT** Eleni G. Hapidou hapidou@hhsc.ca

© 2017 Eleni G. Hapidou and Yuelin Li. Published with license by Taylor & Francis.

This is an Open Access article distributed under the terms of the Creative Commons Attribution License (http://creativecommons.org/licenses/by/3.0/), which permits unrestricted use, distribution, and reproduction in any medium, provided the original work is properly cited. The moral rights of the named author(s) have been asserted.

Introduction/Aim: The purpose of this study was to predict outcomes of interdisciplinary chronic pain management. Outcomes were defined in terms of goal attainment and patient satisfaction after a four-week program. Patients had heterogeneous pain and were referred by insurance companies, worker’s compensation and veterans affairs. They completed a variety of psychometric instruments on pain-related measures at admission and discharge. At discharge, they also provided ratings of goal attainment and program satisfaction.

Methods: Stepwise regression analyses were performed on a large data set of 882 patients who provided complete data at both admission and discharge with goal attainment and program satisfaction scores as the criterion variables. Difference scores between admission and discharge on pain intensity, depression, anxiety, catastrophizing, recent bothersome symptoms, acceptance and readiness to change measures served as the predictors.

Results: Goal attainment was best predicted by the difference scores on readiness to change subscales (pre-contemplation, contemplation and maintenance), recent bothersome symptoms, catastrophizing, and activities engagement subscale of the acceptance measure. Patient satisfaction with treatment was best predicted by the difference scores on pre-contemplation and maintenance, recent bothersome symptoms, and the activities engagement subscale of the acceptance measure.

Discussion/Conclusions: Results show that patients are more likely to evaluate their goal attainment and satisfaction more positively when they also make changes in their readiness to change, they adopt self-management strategies for pain, reduce their symptoms in the past month and are more accepting of their chronic pain problem. These results are discussed in the context of the literature on pain management outcomes.

## Chronic postsurgical pain and long term opioid use after ankle surgery

Matthew Foss^a^, Rob Rideout^b^, Mark Glazebrook^c^, A. John Clark^a^, and Mary E. Lynch^a^

^a^Anesthesiology, Pain Management and Perioperative Medicine, Dalhousie University, Halifax, Nova Scotia, Canada; ^b^Anesthesiology, Pain Management and Perioperative Medicine, Queen Elizabeth II Health Sciences Center, Halifax, Nova Scotia, Canada; ^c^Orthopedic Surgery, Dalhousie University, Halifax, Nova Scotia, Canada

**CONTACT** Matthew Foss matthew.foss@dal.ca

© 2017 Matthew Foss, Rob Rideout, Mark Glazebrook, A. John Clark, and Mary E. Lynch. Published with license by Taylor & Francis.

This is an Open Access article distributed under the terms of the Creative Commons Attribution License (http://creativecommons.org/licenses/by/3.0/), which permits unrestricted use, distribution, and reproduction in any medium, provided the original work is properly cited. The moral rights of the named author(s) have been asserted.

Introduction/Aim: Chronic post surgical pain (CPSP) occurs in 10–50% of patients after common surgical procedures, in 2–10% this is severe. A key predictor of CPSP is severity of initial postoperative pain. It is critical to maximize strategies to improve the management of postoperative pain. The current study examined whether continuous neural blockade would improve outcomes following major ankle surgery.

Methods: This was a pragmatic trial where a program of continuous neural blockade at home was available. It was hypothesized that some patients would not receive continuous neural blockade (eg. patient preference or ineligible block) and that groups may differ in pain control and CPSP. Outcome measures consisted of the Brief Pain Inventory (BPI) pain and interference scales. Patients were followed to 6 months.

Results: Thirty-nine patients were recruited, 34 received continuous neural blockade, 5 inpatient multimodal analgesia. There were large decreases in mean pain severity, *F*(2.37, 90.68) = 27.23, *p* < .001, η_p_^2^ = .42 and pain interference over time, *F*(2.54, 96.39) = 20.08, *p* < .001, η_p_^2^ = .35. Group size was too small to compare difference in CPSP between groups. Pain scores indicated both groups did well. Four patients had CPSP at 3 months and 2 patients (5%) at 6 months, 12 patients were taking an opioid before surgery this was down to 4 patients using an opioid at 6 months.

Discussion/Conclusions: Even with best evidence postsurgical analgesic approaches the rate of CPSP is 5%, to improve beyond this may require a transitional pain service that also addresses psychosocial aspects.

## Critical role of microglial pannexin-1 channels in opioid withdrawal

Nicole E. Burma^a^, Robert P. Bonin^b^, Heather Leduc-Pessah^c^, Corey Baimel^d^, Zoe F. Cairncross^e^, Michael Mousseau^f^, Catherine M. Cahill^g^, Stephanie L. Borgland^h^, and Yves De Koninck^i^, and Tuan Trang^j^

^a^Department of Comparative Biology and Experimental Medicine, Department of Physiology and Pharmacology, Hotchkiss Brain Institute, University of Calgary, Calgary, AB, Canada; ^b^Department of Pharmaceutical Sciences, Leslie Dan Faculty of Pharmacy, University of Toronto, Toronto, ON, Canada; ^c^Department of Comparative Biology and Experimental Medicine, Department of Physiology and Pharmacology, Hotchkiss Brain Institute, University of Calgary, Calgary, AB, Canada; ^d^Department of Physiology and Pharmacology, Hotchkiss Brain Institute, University of Calgary, Calgary, AB, Canada; ^e^Department of Comparative Biology and Experimental Medicine, Department of Physiology and Pharmacology, Hotchkiss Brain Institute, University of Calgary, Calgary, AB, Canada; ^f^Department of Comparative Biology and Experimental Medicine, Department of Physiology and Pharmacology, Hotchkiss Brain Institute, University of Calgary, Calgary, AB, Canada; ^g^Department of Anaesthesiology and Perioperative Care, University of California Irvine, Irvine, California, USA; ^h^Department of Physiology and Pharmacology, Hotchkiss Brain Institute, University of Calgary, Calgary, AB, Canada; ^i^Department of Psychiatry and Neuroscience, Institut Universitaire en santé mentale de Québec, Université Laval, Laval, Québec, Canada; ^j^Department of Comparative Biology and Experimental Medicine, Department of Physiology and Pharmacology, Hotchkiss Brain Institute, University of Calgary, Calgary, AB, Canada

**CONTACT** Nicole E. Burma neburma@ucalgary.ca

© 2017 Nicole E. Burma, Robert P. Bonin, Heather Leduc-Pessah, Corey Baimel, Zoe F. Cairncross, Michael Mousseau, Catherine M. Cahill, Stephanie L. Borgland, Yves De Koninck, Tuan Trang. Published with license by Taylor & Francis.

This is an Open Access article distributed under the terms of the Creative Commons Attribution License (http://creativecommons.org/licenses/by/3.0/), which permits unrestricted use, distribution, and reproduction in any medium, provided the original work is properly cited. The moral rights of the named author(s) have been asserted.

Introduction/Aim: Repeated opioid use can lead to physical dependence, which manifests as a withdrawal syndrome upon cessation of drug use. Converging evidence suggests that opioid withdrawal is critically mediated by cellular changes in the spinal dorsal horn. In the present study, we examined the role of microglial pannexin-1 (Panx1) channels in morphine withdrawal.

Methods: Rats and mice were treated with escalating doses of morphine for 5 days. On day 5, the opioid receptor antagonist naloxone was injected to rapidly precipitate withdrawal behaviours. To assess the role of spinal microglia in withdrawal, Mac1-Saporin was intrathecally injected into morphine dependent animals prior to withdrawal. Mixed adult cell cultures were isolated from the spinal cords of morphine dependent and control animals and analyzed for co-expression of Panx1 and CD11b (a microglial marker) using flow cytometry, and for functional changes in microglial Panx1 using a YO-PRO dye-uptake assay.

Results: Depletion of spinal microglia significantly attenuated the physical signs of morphine withdrawal. Using flow cytometry, we found that morphine treatment increased Panx1 expression exclusively within the microglial population. In acutely isolated spinal microglia, BzATP-evoked YO-PRO dye uptake was potentiated in microglia isolated from morphine dependent animals compared to control animals. To test the role of microglial Panx1 *in vivo*, we genetically deleted Panx1 from Cx3cr1 expressing cells (microglial population) in mice and found that these animals exhibited a robust reduction in withdrawal symptoms.

Discussion/Conclusions: Collectively, our findings reveal a novel and critical role for microglial Panx1 in morphine withdrawal.

## Investigating the relationship between preschooler executive functioning and pain-related distress post-needle

Shaylea Badovinac, Hannah Gennis, and Rebecca P. Riddell

Department of Psychology, York University, Toronto, Ontario, Canada

**CONTACT** Shaylea Badovinac sdbadov@yorku.ca

© 2017 Shaylea Badovinac, Hannah Gennis, and Rebecca Pillai Riddell. Published with license by Taylor & Francis.

This is an Open Access article distributed under the terms of the Creative Commons Attribution License (http://creativecommons.org/licenses/by/3.0/), which permits unrestricted use, distribution, and reproduction in any medium, provided the original work is properly cited. The moral rights of the named author(s) have been asserted.

Introduction/Aim: Between infancy and early childhood, children take on an increasing role in the regulation of their own distress. In the context of pain management, self-regulation involves exerting ‘top-down’ control over behavioural and emotional responses to pain through executive functioning. Our goal was to investigate the relationship between executive functioning and post-needle pain-related distress expression in preschoolers.

Methods: The current study used existing data (*n* = 138) from a longitudinal cohort that video-recorded caregiver-preschooler dyads during routine vaccination appointments. Videotapes were coded for pain-related distress at 1, 2, and 3 minutes post-needle (FLACC; Merkel et al., 1997). Dyads also subsequently participated in a psychoeducational assessment which comprehensively assessed preschooler executive functioning and included the NEPSY Statue subtest (Korkman et al., 1998), the Day/Night task (Gerstadt et al., 1994) and parent- and teacher-rated BRIEF questionnaires (Behavior Rating Inventory of Executive Function; Gioia et al., 2000).

Results: Bonferroni-corrected bivariate correlations revealed no significant relationships between measures of executive function and FLACC scores at 1, 2, or 3 minutes post-needle.

Discussion/Conclusions: Our results suggest that teacher-reported, parent-reported and objective measures (both behavioural and cognitive) of executive functioning are not associated with pain-related distress signalling among preschoolers. This may be due to the fact that preschoolers’ self-regulatory strategies have not yet been consolidated, and thus there are still a number of influential situational (e.g., caregiver behaviour) and dispositional (e.g., temperament) factors that work together to predict post-needle distress while broader self-regulatory abilities (such as those tapped into during psychoeducational assessments) are still being developed.

## The utility of universal urinary drug screening in chronic pain management

Luke Wiseman 0000-0002-7431-4489^a^, and Mary Lynch^b^

^a^Faculty of Medicine, Dalhousie University, Halifax, Nova Scotia, Canada; ^b^Department of Anesthesia, Pain Management & Perioperative Medicine, Department of Pharmacology, Department of Psychiatry, Dalhousie University, Halifax, Nova Scotia, Canada

**CONTACT** Luke Wiseman lukewiseman@dal.ca

© 2017 Luke Wiseman and Mary Lynch. Published with license by Taylor & Francis.

This is an Open Access article distributed under the terms of the Creative Commons Attribution License (http://creativecommons.org/licenses/by/3.0/), which permits unrestricted use, distribution, and reproduction in any medium, provided the original work is properly cited. The moral rights of the named author(s) have been asserted.

Introduction/Aim: To study the prevalence of unexpected tandem mass spectrometry (TMS) urinary drug screening (UDS) results for all new patients presenting at a hospital-based chronic pain center and to assess which drugs are most likely to contribute to an unexpected result. Also, to assess the clinical utilization of unexpected results by pain physicians.

Methods: From June 2014 to June 2016, a total of 664 chronic non-cancer pain (CNCP) patients were seen for initial consult at a hospital-based chronic pain center. Charts were reviewed and used to create a database containing sex, age, UDS result, and medication/illicit drug history. For all unexpected results, an interview was conducted with the treating physician to determine the clinical implications of the UDS.

Results: The overall percentage of patients with an unexpected UDS result was 16.67% for the general pain specialists and 50% for the pain/addictions specialist, with opioids and benzodiazepines contributing the most. Although eight out of nine physicians found UDS helpful in general, only 29.58% of unexpected UDS were helpful in the management of their patients and had a direct influence on their care.

Discussion/Conclusions: The prevalence of unexpected results in UDS in CNCP patients is significant. Most physicians agree that UDS is helpful but in only a limited number of cases did the unexpected result provide helpful information that significantly influenced patient care. When UDS impacted patient care, it provided information to improve collaborative practices and patient-physician communication. It was also used as a method to guide further testing and prescribing.

## Pain assessment and management of premature infants in a Canadian neonatal intensive care unit (NICU): How are we doing?

Adele Orovec^a^, Timothy Disher^b^, and Marsha Campbell-Yeo^b^

^a^Medical Science, Dalhousie University, Halifax, Nova Scotia, Canada; ^b^School of Nursing, Dalhousie University, Halifax, Nova Scotia, Canada

**CONTACT** Adele Orovec aorovec@dal.ca

© 2017 Adele Orovec, Timothy Disher, and Marsha Campbell-Yeo. Published with license by Taylor & Francis.

This is an Open Access article distributed under the terms of the Creative Commons Attribution License (http://creativecommons.org/licenses/by/4.0/), which permits unrestricted use, distribution, and reproduction in any medium, provided the original work is properly cited.

Introduction/Aim: Despite strong evidence that repeated pain exposure in neonates is associated with adverse outcomes, inadequate pain assessment and management has been reported with less than half receiving pain relief. Given the past decade’s emphasis on optimizing care in neonatal settings, there is a need to evaluate the current status of pain assessment and use of pain relieving interventions in this population. The aim of this study is thus to evaluate the level of pain assessment and management in a cohort of hospitalized Canadian preterm neonates.

Methods: A secondary analysis of study data collected from premature neonates enrolled in a clinical trial (Campbell-Yeo et al., 2013) and supplemental chart review.

Results: The 242 neonates included in the study underwent a total of 10468 painful procedures (4801 tissue breaking and 5667 non tissue breaking with only 56.6% and 12.2% having a documented pain score using the Premature Infant Pain Profile (PIPP) respectively). Of those with a documented pain score the most likely procedures to receive a pain score were heel sticks (60.8%), venipunctures (58.4%) and peripherally inserted central catheters (56.8%). The average PIPP score was 4.71 (range 0–21). Procedures least likely to receive a pain score were suctioning (0.2%), tape removals (6.8%), and endotracheal tube insertions (7.4%). A pharmacological pain relieving intervention was provided 40.6%, a nonpharmacological intervention 30.2%, and in combination 28.9% of the time.

Discussion/Conclusions: There was considerable variation in reporting and treatment of pain. Future efforts remain needed to promote consistent pain assessment and management.

## Suicide attempts and completions in chronic pain: A retrospective cohort study

Mary-Ellen Hogan^a^, Anna Taddio^b^, Joel Katz^c^, Vibhuti Shah^d^, and Murray Krahn^e^

^a^Graduate Department of Pharmaceutical Sciences, University of Toronto, Toronto, Ontario, Canada; ^b^Leslie Dan Faculty of Pharmacy, University of Toronto, Toronto, Ontario, Canada; ^c^Department of Psychology, York University, Toronto, Ontario, Canada; ^d^Institute for Health Policy Management and Evaluation, Faculty of Medicine, University of Toronto, Toronto, Ontario, Canada; ^e^Toronto Health Economics and Technology Assessment Collaborative, University of Toronto, Toronto, Ontario, Canada

**CONTACT** Mary-Ellen Hogan maryellenhogan@gmail.com

© 2017 Mary-Ellen Hogan, Anna Taddio, Joel Katz, Vibhuti Shah, and Murray Krahn. Published with license by Taylor & Francis.

This is an Open Access article distributed under the terms of the Creative Commons Attribution License (http://creativecommons.org/licenses/by/3.0/), which permits unrestricted use, distribution, and reproduction in any medium, provided the original work is properly cited. The moral rights of the named author(s) have been asserted.

Introduction/Aim: Little research exists on suicide completion rates in chronic pain. We aimed to describe attempts and completions in a population-based matched sample with and without chronic pain using administrative data.

Methods: Ontarians ≥18 years were identified from the Canadian Community Health Survey. Individuals with and without chronic pain were matched on age, sex, rurality and income using propensity methods and linked administrative data. They were followed from survey response to death or December 31, 2013. Suicide attempts and completions were identified using ICD-10 codes from emergency department records and Ontario death records, respectively. We also employed an accepted broader definition of suicide, which included accidental poisoning and death of undetermined intent. Suicide rates were expressed as number per 100,000 person years.

Results: There were 18,430 pairs of adults with (cases) and without (controls) chronic pain. Average age was 56 years and 61% were female. Mean (SD) follow-up was 6.9 (3.6) years. 133 people attempted suicide at least once among cases versus 66 among controls (P < 0.01). There were 19 suicides among cases compared to 12 among controls (p > 0.05), translating to 15 and 9 suicides per 100,000 person years. For the broad suicide definition, there were 38 and 17 suicides (p < 0.01) with rates of 30 and 13 per 100,000.

Discussion/Conclusions: Suicide attempts and completions (broad definition) occurred twice as frequently in people with chronic pain versus matched controls. There was no statistical difference in completions using the narrow definition. Our results suggest that chronic pain is associated with suicidal behavior.

## Factors predicting quality of life in breast cancer survivors with chronic neuropathic pain

Amanda Carson^a^, E. Khoo^b^, Heather Romanow^b^, Yaad Shergill^b^, Catherine Smyth^c,^^b,^^d^, and Patricia Poulin^b,^^c,^^d,^^e^

^a^Psychology, York University, Toronto, ON, Canada; ^b^Clinical Epidemiology Program, The Ottawa Hospital Research Institute, Ottawa, ON, Canada; ^c^Pain Clinic, The Ottawa Hospital, Ottawa, ON, Canada; ^d^Department of Anesthesiology, The University of Ottawa, Ottawa, ON, Canada; ^e^Department of Psychology, The Ottawa Hospital, Ottawa, ON, Canada

**CONTACT** Amanda Carson carsona@yorku.ca

© 2017 Amanda Carson, Eve-Ling Khoo, Heather Romanow, Yaad Shergill, Catherine Smyth, and Patricia Poulin. Published with license by Taylor & Francis.

This is an Open Access article distributed under the terms of the Creative Commons Attribution License (http://creativecommons.org/licenses/by/3.0/), which permits unrestricted use, distribution, and reproduction in any medium, provided the original work is properly cited. The moral rights of the named author(s) have been asserted.

Introduction/Aim: Chronic neuropathic pain (CNP) affects up to half of all breast cancer survivors. Considering that pharmacological treatments for CNP remain limited, there is ongoing interest in furthering our understanding of patients’ experiences and developing psychosocial interventions such as mindfulness-based stress reduction (MBSR), which demonstrates promising benefits for those with CNP. To explore how one’s quality of life (QOL) might be impacted by CNP, we investigated predictors of QOL, specifically mindfulness.

Methods: Although this study is part of a larger randomized control trial assessing the effects of MBSR, a cross-sectional survey conducted prior to the intervention consisted of 103 women (Mean age=53, *SD*=10.27) with CNP who completed the following self-report measures: Five Facets Mindfulness Questionnaire, Brief Pain Inventory, Short Form Health Survey-12-v2 and Profile of Mood States-2A.

Results: Participants reported moderate to severe pain on a 10-point scale (*M*=4.80, *SD*=1.80). Mental health-related QOL (MHQOL) was positively related to mindfulness and negatively related to pain intensity and mood disturbance. Physical health-QOL was not related to mindfulness. Using hierarchal regression, mindfulness predicted 43% of the MHQOL variance after controlling for age and pain intensity (*F*(7,88)=11.40, *p*<.001, *R*^2^*Adjus- ted*=.43).

Discussion/Conclusions: Consistent with previous research, a relationship exists between mindfulness and pain-related outcomes such as QOL. Given the negative impact a diminished QOL may have, further investigation in mindfulness and MBSR for CNP is warranted. Such investigations may include examining relationships between the above-mentioned outcomes before and after mindfulness training.

## The association of acute postoperative pain with chronic postsurgical pain following cardiac surgery: A systematic review of observational studies

Shaunattonie Henry^a^, Jennifer Yost^a^, Sandra Carroll^a^, Jason W. Busse^b^, J. Charles Victor^c^, and Michael McGillion^d^

^a^School of Nursing, McMaster University, Hamilton, Ontario, Canada; ^b^Department of Health Research Methods, Evidence, and Impact, McMaster University, Hamilton, Ontario, Canada; ^c^Institute of Health Policy, Management and Evaluation, University of Toronto, Toronto, Ontario, Canada; ^d^School of Nursing, McMaster University, Hamilton, Ontario, Canada

**CONTACT** Shaunattonie Henry henrys1@mcmaster.ca

© 2017 Shaunattonie Henry, Jennifer Yost, Sandra Carroll, Jason W. Busse, J. Charles Victor, and Michael McGillion. Published with license by Taylor & Francis.

This is an Open Access article distributed under the terms of the Creative Commons Attribution License (http://creativecommons.org/licenses/by/3.0/), which permits unrestricted use, distribution, and reproduction in any medium, provided the original work is properly cited. The moral rights of the named author(s) have been asserted.

Introduction/Aim: Despite the demonstrated survival and symptom-related benefits of cardiac surgery, up to 56% of patients develop chronic post-surgical pain (CPSP). Most established risk factors associated with CPSP following cardiac surgery are non-modifiable (e.g. age). We conducted a systematic review to explore the association between the intensity of acute postoperative pain and the development of CPSP following cardiac surgery.

Methods: We searched CINAHL, EBSCOhost, Cochrane Library, Clinical Evidence, Medline PubMed, ProQuest and Ovid from 1997-2017. Eligible studies enrolled patients undergoing cardiac surgery and, in an adjusted model, reported the association between acute post-surgical pain intensity and the development of CPSP. We appraised the quality of studies using Critical Appraisal Skills Programme Checklist and risk for bias using Quality in Prognostic Studies tool. We used inverse-variance, random-effects meta-analysis to summarize standardized mean differences (SMD) (associated 95% CI) to examine the strength of association between acute postop pain and CPSP.

Results: Six studies, involving 1,812 patients, were eligible. Methodological quality ranged from high to low; key methodological issues included heterogeneity and reliability of measurement approaches, chart auditing strategies and attrition. Acute post-operative pain showed a small association with the development of CPSP (SMD 0.28; 95%CI 0.12 to 0.44).

Discussion/Conclusion: Our review suggests there is a small association between greater intensity of acute post-operative pain and the development of CPSP after cardiac surgery. As such, any reduction in persistent pain achieved through pharmacological reduction of acute post-operative pain will likely be obscured by the random error from all other determinants of long-term pain.

## The influence of caregiver culture on preschool pain expression

Drexler K. Ortiz^a^, Monica C. O’Neill^a^, Rebecca R. Pillai Riddell^b^, Hartley Garfield^c^, and Saul Greenberg^c^

^a^Psychology, York University, Toronto, Ontario, Canada; ^b^Psychology, Psychiatry, York University, Hospital for Sick Children, & University of Toronto, Toronto, Ontario, Canada; ^c^Pediatrics, Hospital for Sick Children & University of Toronto, Toronto, Ontario, Canada

**CONTACT** Drexler Klein Ortiz drexlerk@my.yorku.ca

© 2017 Drexler Klein Ortiz, Monica C. O’Neill, Rebecca R. Pillai Riddell, Hartley Garfield, and Saul Greenberg. Published with license by Taylor & Francis.

This is an Open Access article distributed under the terms of the Creative Commons Attribution License (http://creativecommons.org/licenses/by/3.0/), which permits unrestricted use, distribution, and reproduction in any medium, provided the original work is properly cited. The moral rights of the named author(s) have been asserted.

Introduction/Aim: To examine if caregiver sensitivity mediates the relationship between caregiver culture and preschool pain at 1 and 2 minutes post-needle.

Methods: A subsample (*N* = 154) of preschooler-caregiver dyads was examined from a longitudinal cohort following routine immunization appointments from infancy through preschool. Preschooler pain behaviors were coded at 1 and 2 minutes post-needle (FLACC).^1^ Caregiver sensitivity was coded using The Maternal Behavior Q-Set Short Form (MBQS-SF).^2^ Caregiver culture was operationalized according to an objectively derived individualism rating of caregivers’ self-reported heritage culture.^3^

Results: Two mediation models showed that the indirect effect of caregiver culture on preschooler pain, through caregiver sensitivity, was non-significant at 1 minute (*AB* = −.017; 95% CI [−.167, .065]) and 2 minutes post-needle (*AB* = −.011; 95% CI [−.151, .079]).

Discussion/Conclusions: Caregiver sensitivity did not mediate the relationship between caregiver culture and preschool pain expression at 1 and 2 minutes post-needle. The present findings differ from analyses during infancy wherein caregiver emotional availability mediated the relationship between the level of individualism of caregivers’ self-reported heritage culture and infant pain.^4^ While preliminary, the discrepancy between the infant and preschool relationships suggests that caregiver culture may have less influence on caregiver sensitivity and in turn pain expression during the preschool vaccination. Preschoolers are more mature in their pain self-regulatory strategies and thus parental factors (such as culture and sensitivity) may have different impacts than during infancy.

### References

1. Merkel, S. I., Voepel-Lewis, T., Shayevitz, J. R., & Malviya, S. (1997). **The FLACC: a behavioral scale for scoring postoperative pain in young children**. Pediatric nursing, 23(3), 293.

2. Tarabulsy, G. M., Provost, M. A., Bordeleau, S., Trudel-Fitzgerald, C., Moran, G., Pederson, D. R., … & Pierce, T. (2009). **Validation of a short version of the maternal behavior Q-set applied to a brief video record of mother-infant interaction**. Infant Behavior and Development, 32(1), 132–136.

3. Taras, V., Steel, P., & Kirkman, B. L. (2012). **Improving national cultural indices using a longitudinal meta-analysis of Hofstede’s dimensions**. Journal of World Business, 47(3), 329–341.

4. O’Neill, M. C., Riddell, R. P., Garfield, H., & Greenberg, S. (2016). **Does Caregiver Behavior Mediate the Relationship Between Cultural Individualism and Infant Pain at 12 Months of Age?**. The Journal of Pain, 17(2), 1273–1280.

## Predictors of suboptimal pain relief following spinal cord stimulation - A retrospective study

Manikandan Rajarathinam^a^, Harsha Shanthanna^a^, Phil Chan^a^, Mauricio Forero^a^, Ahmed Abdulhadi Al Jishi^b^, Raffy Gutman^c^, Mary Van Doorn^d^, Frazier Frances^d^, and Tony Tidy^e^

^a^Anesthesia/Pain medicine, McMaster University, Hamilton, ON, Canada; ^b^Neurosurgery, Hamilton Health Sciences/McMaster University, Hamilton, ON, Canada; ^c^Anesthesiology, McMaster University, Hamilton, ON, Canada; ^d^Neuromodulation, Hamilton Health Sciences, Hamilton, ON, Canada; ^e^Department of Anesthesia, McMaster University, Hamilton, ON, Canada

**CONTACT** Manikandan Rajarathinam drmani.ab8@gmail.com

© 2017 Manikandan Rajarathinam, Harsha Shanthanna, Phil Chan, Mauricio Forero, Ahmed Abdulhadi Al Jishi, Raffy Gutman, Mary Ann Van Doorn, Frazier Frances, and Tony Tidy. Published with license by Taylor & Francis.

This is an Open Access article distributed under the terms of the Creative Commons Attribution License (http://creativecommons.org/licenses/by/3.0/), which permits unrestricted use, distribution, and reproduction in any medium, provided the original work is properly cited. The moral rights of the named author(s) have been asserted.

Introduction/Aim: Spinal cord stimulation (SCS) is a long term therapy for certain chronic pain conditions where conservative modalities of pain management have failed. This is a retrospective study to assess the failure rate of SCS therapy in the cohort of patients over the past 5 years in our Institution and the identifiable factors that might have contributed to the failure.

Methods: The neuromodulation program registry will be screened for SCS implantation records and patient contact details. The parameters like demographics, smoking history, duration of chronic pain and opioid dose prior to SCS, indication, implantation details will be obtained from the records. The patients will be telephonically contacted to assess the degree of pain relief immediately following the procedure, the continuity of pain relief and the current degree of pain relief in relation to initial relief. The patients will be asked to rate the overall Global impression of Change in a 7 point scale. Based on this information, they will be categorized in to positive responders (>50% pain relief) and negative responders (<50% pain relief). The incidence of failure with SCS therapy and the factors predicting treatment failures are the primary outcomes of the study. The change in global health status and the incidence of adverse effects are the secondary outcomes.

Results/Conclusion: 65 patients were identified by the screening of the Neuromodulation registry and are being contacted telephonically to assess the outcome of treatment. The results of the study and conclusion will be presented in the meeting.

## Exploring the movement of knowledge among health care providers using the echo chronic pain model in Ontario

Naima Salemohamed^a^, Jennifer Stinson^a^, Jane Zhao^b^, Leslie Carlin^c^, Ruth Dubin^d^, Paul Taenzer^e^, Fiona Webster^f^, Emily Seto^a^, and Andrea Furlan^g^

^a^Institute of Health Policy, Management and Evaluation, University of Toronto, Toronto, Ontario, Canada; ^b^ECHO Ontario: MSK Department, Toronto Rehabilitation Institute, Toronto, Ontario, Canada; ^c^Physical Therapy, University of Toronto, Toronto, Ontario, Canada; ^d^Department of Family Medicine, Queens University, Kingston, Ontario. Canada; ^e^Department of Psychology, University of Calgary, Calgary, Alberta, Canada; ^f^Dalla Lana School of Public Health, University of Toronto, Toronto, Ontario, Canada; ^g^ECHO Program: MSK Department, Toronto Rehabilitation Institute, Toronto, Ontario, Canada

**CONTACT** Naima Salemohamed naima.salemohamed@mail.uToronto.ca

© 2017 Naima Salemohamed, Jennifer Stinson, Jane Zhao, Leslie Carlin, Ruth Dubin, Paul Taenzer, Fiona Webster, Emily Seto, and Andrea Furlan. Published with license by Taylor & Francis.

This is an Open Access article distributed under the terms of the Creative Commons Attribution License (http://creativecommons.org/licenses/by/3.0/), which permits unrestricted use, distribution, and reproduction in any medium, provided the original work is properly cited. The moral rights of the named author(s) have been asserted.

Introduction/Aims: The ECHO Ontario Chronic Pain Program is a telementoring (telehealth) platform, which supports health care providers (HCPs; spokes) in managing their own patients with chronic pain in their home communities, using the expertise of subspecialists (hub). The aims for this project include understanding: a) if HCPs have increased knowledge and skills in pain management and opioid stewardship, b) whether ECHO’s community of practice model impacted HCPs’ knowledge sharing and c) how HCPs in this program gained insights into their motivations and confidence levels in managing this challenging population.

Methods: Thirteen qualitative semi-structured interviews were completed with participants who have completed or are still attending ECHO. A representative sample of HCPs were included (a) rural vs. urban settings, (b) those who presented ECHO cases vs. others who chose not to, and (c) different professions of HCPs.

Results: Preliminary results have demonstrated that the ECHO Ontario Chronic Pain Program supports collaboration by providing a variety of views from the different disciplines, having a respectful space to listen while learning, and supporting the building of links with other HCPs through this virtual community. Additionally, HCPs value the different resources available and tools being taught by the expert team.

Discussion/Conclusions: These preliminary results suggest that the ECHO Ontario Chronic Pain Program has built a supportive network of HCPs and it promotes continuous learning. Further analysis will provide the opportunity to understand the reach of ECHO and to learn how ECHO is having a impact at the community level in rural, remote, and underserved areas.

## Non-ionotropic NMDA receptor activity contributes to reversal of hyperalgesia and sensitization in pain reconsolidation

A. D’Souza, Y. Xie, Irene Lecker, and Robert Bonin

Pharmaceutical Sciences, University of Toronto, Toronto, Ontario, Canada

**CONTACT** Abigail D’Souza abigail.dsouza@mail.utoronto.ca

© 2017 Abigail D’Souza, Yu-Feng Xie, Irene Lecker, and Robert Bonin. Published with license by Taylor & Francis.

This is an Open Access article distributed under the terms of the Creative Commons Attribution License (http://creativecommons.org/licenses/by/3.0/), which permits unrestricted use, distribution, and reproduction in any medium, provided the original work is properly cited. The moral rights of the named author(s) have been asserted.

Introduction/Aim: Pathological pain can arise from synaptic plasticity within nociceptive pathways of the spinal cord dorsal horn. We have previously shown that the reactivation of sensitized pain pathways triggers a process that parallels memory reconsolidation, in which potentiated synapses undergo a simultaneous process of potentiation and depotentiation. Pharmacologically isolating the depotentiation process can reverse synaptic potentiation in the dorsal horn and erase hyperalgesia.

Reconsolidation is crucially dependent on NMDA receptor activity. Recent work has suggested that NMDA receptors have both ionotropic and non-ionotropic effects that are associated with synaptic potentiation and depotentiation, respectively. Here, we test the hypothesis that non-ionotropic NMDA activity (NI-NMDA) mediates depotentiation in pain reconsolidation to reverse hyperalgesia.

Methods: Whole-cell patch clamp in acutely isolated spinal cord slices and an *ex vivo* spinal cord explant model were used to study long-term potentiation (LTP) in the dorsal horn. Mechanical sensitization was induced in behavioural studies by injection of capsaicin in the hind paw.

Results: The combination of neuronal activity and pharmacological isolation of NI-NMDA with 7-chlorokynurenate (7-CK) caused the reversal of dorsal horn LTP. The application of 7-CK alone did not reverse LTP. Behaviourally, intrathecal administration of 7-CK in combination with plantar capsaicin reversed mechanical hyperalgesia, while nonspecific blockade of NMDA activity did not.

Discussion/Conclusions: These data indicate a key role for NI-NMDA in pain reconsolidation and identify this signaling pathway as a potential novel target for lasting relief. Future work aims to isolate the mechanisms of synaptic depotentiation and cellular changes that enable NI-NMDA during sensitization.

## Supporting Parents Acute Lymphoblastic Leukemia (ALL) Pain Care involvement: A Qualitative Interpretive Description

Amanda Bettle^a^, Margot Latimer^a,^^b^, Conrad Fernandez^a,^^b^, and Jean Hughes^a,^^b^

^a^IWK Health Centre, Halifax NS, Canada; ^b^Dalhousie University, School of Nursing, Halifax NS, Canada

**CONTACT** Margot Latimer Margot.Latimer@iwk.nshealth.ca

© 2017 Amanda Bettle, Margot Latimer, Conrad Fernandez, and Jean Hughes. Published with license by Taylor & Francis

This is an Open Access article distributed under the terms of the Creative Commons Attribution License (http://creativecommons.org/licenses/by/3.0/), which permits unrestricted use, distribution, and reproduction in any medium, provided the original work is properly cited. The moral rights of the named author(s) have been asserted.

Background: Children with Acute Lymphoblastic Leukemia experience pain from the disease, treatment and procedures. Parents can be effective in managing their child’s pain but little is known about how they learn to do this. Aims: The purpose of this study was to describe parent and pediatric oncology nurse perspectives on what parents do to relieve their child’s pain during ALL treatment. A second purpose was to identify key structures and processes that facilitate parents’ optimal pain care involvement. Methods: This study was framed by Appreciative Inquiry (Cooperrider, 2008) and Interpretive Descriptive methods (Thorne, 2008) were used to describe: pain sources, parents’ pain care role, and key structures supporting their pain care involvement. Results: Eight clinic nurses and ten parents participated and six key themes per group were identified. Parent themes included: *establishing therapeutic relationships, relearning how to care for my child, overcoming challenges and recognizing pain, learning parent specific strategies, empowered to take active pain care role, and maintaining relationships*. Nurse themes included: *establishing relationships, preparing parents to care for their child, facilitating pain assessment, teaching parents best pain care, empowering parents, and maintaining relationships*. Conclusions: These findings can be used to guide clinical practice and future research.

## Current practices in defining and classifying adverse events, including worsening pain, in massage and manual therapies research: A scoping study.

Donelda Gowan, Anne Leis

Department of Community Health & Epidemiology, College of Medicine, University of Saskatchewan, Saskatoon, Saskatchewan, Canada

**CONTACT** Donelda Gowan d.gowanmoody@usask.ca

© 2017 Donelda Gowan and Anne Leis. Published with license by Taylor & Francis.

This is an Open Access article distributed under the terms of the Creative Commons Attribution License (http://creativecommons.org/licenses/by/3.0/), which permits unrestricted use, distribution, and reproduction in any medium, provided the original work is properly cited. The moral rights of the named author(s) have been asserted.

Aims: Potentially harmful patient outcomes, including mild worsening of pain severity, are common patient safety incidents reported in massage and manual therapies research. There is, however, a common complaint that uniformity and consensus regarding what constitutes an adverse event is lacking. A scoping review was conducted to explore definitions and taxonomies used to operationalize potentially harmful outcomes across disciplines that use ‘hands-on’ manual therapy.

Methods: Based on the methodology of Arksey and O’Malley a six stage scoping review was conducted. Eight electronic databases were searched. Inclusion and exclusion criteria were applied for screening then data was extracted and charted to collate and synthesize material ending in a stakeholder consultation to support knowledge translation.

Results: A total of 967 records were identified and 14 were relevant to our study objectives and included in our final sample. Reporting of mild, minor and transient worsening of pain is common in the manual therapy research from massage therapy, acupuncture, chiropractic, physiotherapy, osteopathy and naprapathy. The duration, intensity and impact on function are the common elements used in taxonomies and numeric rating scales are common means to describe patient outcomes in the study of potential harm.

Conclusion: There is a lack of uniform definition and taxonomy to describe adverse patient outcomes such as worsening pain in patient safety research. The scoping review has provided a useful characterization of where consensus and debate exists.

## Flurbiprofen 8.75 mg spray or lozenge provides relief from sore throat pain in patients positive or negative for beta-haemolytic streptococci

Adrian Shephard^a^, Valeria Bychkova^b^, Joanne Hunt^c^, Natalia Burova^d^, and Eugenia Radkova^e^

^a^Reckitt Benckiser Healthcare International Ltd, Slough, Berkshire, UK; ^b^Evidence Generation & Clinical Research, Reckitt Benckiser, Moscow, Russia; ^c^Respiratory Medical Science, Health Relief, Reckitt Benckiser, Slough, Berkshire, UK; ^d^Federal State Establishment Clinical Diagnostic Medical Center Department, Saint Petersburg, Russia; ^e^OCT Clinical Trials, Saint Petersburg, Russia

**CONTACT** Adrian Shephard Adrian.Shephard@rb.com

© 2017 Reckitt Benckiser. Published with license by Taylor & Francis.

This is an Open Access article distributed under the terms of the Creative Commons Attribution License (http://creativecommons.org/licenses/by/3.0/), which permits unrestricted use, distribution, and reproduction in any medium, provided the original work is properly cited. The moral rights of the named author(s) have been asserted.

Introduction/Aim: Approximately 10% of sore throats are caused by a bacterial infection (Worrall. Can Fam Physician 2007;53:1961–2); therefore, antibiotics are not usually warranted. The NSAID, flurbiprofen, has been developed as both a lozenge and spray to relieve sore throat symptoms. This analysis investigated the effect of flurbiprofen 8.75 mg spray and lozenge on sore throat pain intensity in patients positive or negative for beta-haemolytic streptococci group A or C.

Methods: A randomised, double-blind study was conducted across 16 centres in Russia in adult patients with acute sore throat. At baseline, beta-haemolytic streptococci were identified by throat swab culture. Patients were randomly assigned to take one dose of flurbiprofen 8.75 mg spray plus placebo lozenge (n=218), or flurbiprofen 8.75 mg lozenge plus placebo spray (n=222), and were asked to rate sore throat pain on the Sore Throat Pain Intensity Scale (STPIS; 100-mm visual analogue scale, ‘no pain’ to ‘severe pain’).

Results: Similar proportions of patients in the spray (6.5%, 13/201) and lozenge (4.3%, 9/210) groups were positive for beta-haemolytic streptococci (group A or C). Mean±standard deviation change from baseline in STPIS at 2 hours post-dose was similar between patients positive for beta-haemolytic streptococci (-36.5±20.89 mm for spray and -44.2±24.83 mm for lozenge) and negative (-41.1±21.84 mm for spray and -40.1±22.28 mm for lozenge). Swab testing results were not known until after clinical evaluations were completed and patients did not take antibiotics during the study.

Discussion/Conclusions: Both flurbiprofen 8.75 mg spray and lozenge provide relief from sore throat pain in patients positive or negative for beta-haemolytic streptococci (group A or C).

## A pilot focus group study of *Farmooo*, a virtual reality pain distraction game designed for 14- to 18-year-old patients undergoing chemotherapy

Janice Ng, Henry Lo, Xin Tong, Weina Jin, Diane Gromala, Caron Strahlendorf

**CONTACT** Janice Ng janicewzn@gmail.com

© 2017 Janice Ng, Henry Lo, Xin Tong, Weina Jin, Diane Gromala, and Caron Strahlendorf. Published with license by Taylor & Francis

This is an Open Access article distributed under the terms of the Creative Commons Attribution License (http://creativecommons.org/licenses/by/3.0/), which permits unrestricted use, distribution, and reproduction in any medium, provided the original work is properly cited. The moral rights of the named author(s) have been asserted

Introduction/Aim: *Farmooo* is an immersive Virtual Reality (VR) farm simulation game designed to help distract teenage cancer patients from pain during their chemotherapy treatments. The research is collaboration between BC Children’s Hospital and Simon Fraser University’s Pain Studies Lab.

Methods: A 2.25-hour Focus Group was conducted with 6 outpatients (for their comparatively risk-free potential). The study session comprised: a presentation about the design of *Farmooo*, game testing, a post-test questionnaire and a discussion circle.

Results: Participants expressed deep appreciation that a VR game was built specifically for them. Some concerns were raised about interaction with an attached IV, warm hands necessary for accurate gesture detection and desire for more challenging gameplay. Most participants enjoyed the game, particularly the experience of feeling immersed in the 3D interactive VR environment (M = 75.33, SD = 26.83). All showed high interest in and positive attitudes about using a more robust VR game if they were still undergoing chemotherapy (M = 87.17, SD = 11.41). Nausea associated with VR was much less concerning than expected.

Discussion/Conclusion: Results gleaned from this study provide a basis for better understanding the specific needs and attitudes of teens who may use VR like *Farmooo* as a means of distraction during chemotherapy. The researchers intend to enhance the current VR game according to the findings and will work with BC Children’s Hospital to ensure *Farmooo* can become accessible for patients in sustainable ways.

## Nursing Students’ Knowledge and Attitudes Regarding Pain

Jennifer Hroch^a^, Elizabeth VanDenKerkhof^a,^^b^, Mona Sawhney^a^, Nancy Sears^c^, and Laurie Gedcke-Kerr^a^

^a^School of Nursing, Queen's University, Kingston, ON, Canada; ^b^Department of Anesthesiology & Perioperative Medicine, Queen’s University, Kingston, ON, Canada; ^c^St. Lawrence College, Kingston, ON, Canada

**CONTACT** Elizabeth VanDenKerkhof ev5@queensu.ca

© 2017 Jennifer Hroch, Elizabeth VanDenKerkhof, Mona Sawhney, Nancy Sears, and Laurie Gedcke-Kerr. Published with license by Taylor & Francis

This is an Open Access article distributed under the terms of the Creative Commons Attribution License (http://creativecommons.org/licenses/by/3.0/), which permits unrestricted use, distribution, and reproduction in any medium, provided the original work is properly cited. The moral rights of the named author(s) have been asserted.

Aim: National and international practice guidelines exist for the management of acute and chronic pain. Nurses are key contributors to interprofessional pain management teams, and therefore, it is important to understand their knowledge and attitudes about pain before they enter independent practice. The aim of this study was to assess pain knowledge and attitudes of nursing students in Ontario.

Methods: Using a cross-sectional, descriptive design, 336 final year bachelorette and practical nursing students were recruited from 4 sites (2 schools) in eastern Ontario through convenience sampling. They completed a paper version of the Knowledge and Attitudes Survey Regarding Pain (KASRP). Descriptive statistics and bivariable and multivariable linear regression were conducted. Statistical significance was set at p≤.05.

Results: The mean KASRP score (SD) was 66.7% (9.1). English as the first language, attending the bachelorette program in School A, and having prior experience caring for someone in pain were independently associated with a statistically significant increase in KASRP scores. Common areas of weakness were related to understanding populations at risk of respiratory depression after receiving an opioid, dosage calculations, medication administration and pharmacology. Areas of strength were related to understanding that pain is subjective, reliability of children’s report of pain, and symptoms of withdrawal from opioids.

Conclusions: Further research is needed to gain a broader understanding of pain knowledge and attitudes of nursing students and practicing clinicians across Ontario and Canada. A longitudinal study would improve our understanding of how knowledge and attitudes change as students enter independent clinical practice.

## A Novel Approach to Patient-Centered Pain Medicine in Ontario

A. Mailis^a,^^b^, K. Spivak^a^, and H. Ghori^a^

^a^Pain and Wellness Centre, Vaughan Ontario, Canada; ^b^Comprehensive Integrated Pain Program, University Health Network, Ontario, Canada

**CONTACT** A. Mailis angela.mailis@uhn.ca

© 2017 A. Mailis, K. Spivak, and H. Ghori. Published with license by Taylor & Francis.

This is an Open Access article distributed under the terms of the Creative Commons Attribution License (http://creativecommons.org/licenses/by/3.0/), which permits unrestricted use, distribution, and reproduction in any medium, provided the original work is properly cited. The moral rights of the named author(s) have been asserted.

Aim: To present a) the development of a publicly funded interdisciplinary non-interventional pain management program in the Greater Toronto Area and b) demographic/outcome data of community treated chronic pain patients.

Methods: The Pain Team established an extensive demographic questionnaire, a battery of validated instruments, a consent form outlining obligations/expectations, and inclusion/exclusion criteria. Data were collected from participants given a customized treatment plan which outlined duration, goals, and types of interventions and whenever possible compared to those of downtown academic pain programs.

Results: Preliminary analysis presents only demographics of the first 33 participants [21 females, 12 males, aged 19-83 (mean age 45), pain duration 0.5-30 years (mean 7.6), 40% Canadian born]. Causes of pain were equally split between motor vehicle accidents, sport traumas and work place injuries, and diseases. One third had received multiple injections in the past; 30% consumed marijuana (only in half medically prescribed); 1/3 were unable to work due to pain; 45% were employed; commonest pain site was low back. Significant biomedical pathology was observed in 92% of the males and 33% of the females (43% of them had multisite/widespread pain as compared to none of the males). Opioid consumption was negligible in both genders.

Conclusion: Compared to academic hospital-based pain patient samples, significant differences were observed in terms of ethnic origin, work status, and opioid consumption, as well as between males and females. Detailed demographic data, dropout rates, outcomes, and barriers despite provision of free pain services, will be presented once analyses are completed.

## Best of a bad bunch: A network meta-analysis of pain-relieving treatments for retinopathy of prematurity eye exams

T. Disher^a^, C. Cameron^b^, S. Mitra^c^, and M. Campbell-Yeo^d^

^a^Dalhousie School of Nursing, Halifax, NS, Canada; ^b^Cornerstone Research; ^c^IWK Health Centre, Department of Pediatrics, Division of Neonatology; ^d^Dalhousie University School of Nursing and Department of Psychology and Neuroscience;IWK Health Centre Department of Pediatrics, Division of Neonatology

© 2017 T. Disher, C. Cameron, S. Mitra, and M. Campbell-Yeo. Published with license by Taylor & Francis.

This is an Open Access article distributed under the terms of the Creative Commons Attribution License (http://creativecommons.org/licenses/by/4.0/), which permits unrestricted use, distribution, and reproduction in any medium, provided the original work is properly cited. The moral rights of the named author(s) have been asserted.

Background: Preterm neonates at risk of developing retinopathy of prematurity (RoP) undergo frequent and painful eye exams. Currently, no consensus regarding the optimal way to manage this pain has been reached. A lack of head-to-head randomized clinical trials (RCT) comparing possible pain relieving treatment options has contributed to this problem.

Aim: To estimate the relative efficacy and safety of all available interventions intended to reduce pain from RoP exams.

Methods: We searched MEDLINE, Embase, Cochrane CENTRAL, and Web of Science for randomized controlled trials comparing at least two pain-relieving interventions. Two reviewers performed study selection, data extraction, and quality appraisal. We performed a network meta-analysis using a random-effect model.

Results: Fifteen trials (n = 915 infants), evaluating nine treatments reported results from a validated pain assessment tool. Sweet taste + topical anesthetic, and multisensory (engagement of multiple senses e.g. gustatory + non nutritive sucking (NNS)) interventions + topical anesthetic showed statistically significant improvement over topical anesthetic alone (MD: -1.78; -3.03), although absolute scores still indicated moderate pain. Neither acetaminophen, digital retina imaging, sweet taste alone, or NNS combined with anesthetic showed significant improvement over anesthetic alone. Four studies reported adverse events with no differences in rates of events between anesthetic drops alone or in combination with sweet taste, Tylenol, multi-sensory interventions, or NNS.

Conclusions: Combining topical anesthetic with sweet taste or multisensory interventions is likely to provide the most effective pain relief for RoP exams, although no treatment reduces overall scores to ranges considered to indicate low or no pain.

## The association between pain behaviors and partners’ responses: Explaining the role of partner burden and relationship quality

Somayyeh Mohammadi^a,^^b^, Christine T. Chambers^a,^^c^, and Natalie O. Rosen^d^

^a^Centre for Pediatric Pain Research, IWK Health Centre, Halifax, Nova Scotia, Canada; ^b^Department of Pediatrics, Dalhousie University, Halifax, Nova Scotia, Canada; ^c^Departments of Pediatrics and Psychology & Neuroscience, Dalhousie University, Halifax, Nova Scotia, Canada; ^d^Departments of Psychology and Neuroscience, Obstetrics & Gynaecology, Dalhousie University, Halifax, Nova Scotia, Canada

**CONTACT** Somayyeh Mohammadi smh.mohammadi@hotmail.com

© 2017 Somayyeh Mohammadi, Christine Chambers, and Natalie Rosen. Published with license by Taylor & Francis.

This is an Open Access article distributed under the terms of the Creative Commons Attribution License (http://creativecommons.org/licenses/by/3.0/), which permits unrestricted use, distribution, and reproduction in any medium, provided the original work is properly cited. The moral rights of the named author(s) have been asserted.

Aims: Pain expressions encourage supportive responses of partners such as solicitous and distracting responses; however, expressing pain may not always be related to supportive responses. Gaining an enhanced knowledge of the pathways from pain expressions to partner responses provides a better understanding of the reasons behind different partner responses. We hypothesized that in individuals with chronic pain, expressing pain would be associated with greater partner burden. Consequently, greater partner burden would be linked to poorer relationship satisfaction. Finally, poorer relationship satisfaction would be associated with expressing more negative and less solicitous and distracting responses.

Methods: In an online survey, 125 participants with chronic pain completed standardized questionnaires of pain behaviors, perceptions of partner burden, relationship satisfaction and partner responses to their pain. For each partner response, a separate serial mediation analysis was performed. In each analysis, the independent variable was pain expression, partner burden was the first mediator, followed by relationship satisfaction as the second mediator.

Results: Expressing more pain was associated with higher levels of partner burden. Consequently, higher perception of partner burden was related to lower levels of relationship satisfaction. Finally, lower relationship satisfaction was associated with more punishing and less solicitous and distracting responses.

Conclusions: The findings suggest that in individuals with chronic pain, expressing pain might be related to their perception of their partner well-being, and consequently, to their relationship satisfaction and their partner responses.

Keywords: Pain behaviors, responses, partners, burden, relationship quality

## Biological and psychological predictors of disability severity and change in pain sites in rheumatoid arthritis

Abi Muere^a^, Nadil Zeiadin^b^, Matthew Woo^b^, Hayley Yurgan^a^, Phylicia Verreault^a^, Dean Tripp^c^, and Mala Joneja^b^

^a^Psychology, Queen’s University, Kingston, Ontario, Canada; ^b^Department of Medicine, Queen’s University, Kingston, Ontario, Canada; ^c^Psychology, Anaesthesiology, Urology, Queen’s University, Kingston, Ontario, Canada

**CONTACT** Abi Muere abigail.muere@queensu.ca

© 2017 Abi Muere, Nadil Zeiadin, Matthew Woo, Hayley Yurgan, Phylicia Verreault, Dean Tripp, and Mala Joneja. Published with license by Taylor & Francis.

This is an Open Access article distributed under the terms of the Creative Commons Attribution License (http://creativecommons.org/licenses/by/3.0/), which permits unrestricted use, distribution, and reproduction in any medium, provided the original work is properly cited. The moral rights of the named author(s) have been asserted.

Introduction/Aim: Rheumatoid arthritis (RA) is a long-term inflammatory disease condition characterized by painful joint inflammation, often in the hands and feet. The current goal of treatment is symptom remission; however, only a minority of patients achieve full remission. The present study aimed to identify biological and psychological factors that predict disability severity and change in number of pain sites 6 months post-baseline.

Methods: RA patients (*N* = 109; 86 women, 23 men) recruited from tertiary care completed questionnaires (Pain Catastrophizing Scale, Patient Health Questionnaire-9, Brief Resilience Coping Scale, Health Assessment Questionnaire) and a physician-administered measure of objective disease activity (DAS-28) at baseline. Patients completed the same questionnaires 6 months post-baseline. A hierarchical linear regression was conducted to identify predictors of disability severity. A logistic regression was conducted to identify predictors of change in number of pain sites.

Results: In the final model, baseline disease activity (unstandardized *b* = .162), age (unstandardized *b* = .019), and catastrophizing (unstandardized *b* = .20) were positive predictors of disability severity 6 months post-baseline (*t*s > 3.24, *p*s < .002). Psychological factors significantly predicted disability severity above and beyond biological factors (*F*(3,94) = 9.00, *p* < .001). Approximately 55% of participants reported fewer pain sites 6 months post-baseline. However, no biological or psychological factors were identified to have significantly increased the odds of having fewer pain sites.

Discussion/Conclusions: Both biological and psychological factors play an important role in RA-related disability. Catastrophizing should be considered a critical target for management of RA-related disability. Further research is needed to identify predictors of change in number of pain sites in an RA population.

## The potential use of a serious game to help patients learn about post-operative pain management - an evaluation study

Brynja Ingadottir^a,^^b,^^c^, Sigridur Zoëga^a,^^c^, Katrin Blöndal^a^, David Thue^d^, Ingela Thylen^e^, and Tiny Jaarsma^b^

^a^Surgical Services, Landspitali – The National University Hospital of Iceland, Reykjavík, Iceland; ^b^Department of Social and Welfare Studies, Linköping University, Linköping, Sweden; ^c^University of Iceland, Faculty of Nursing, Reykjavik, Iceland; ^d^School of Computer Science, Reykjavik University, Reykjavik, Iceland; ^e^Department of Cardiology and Department of Medical and Health Sciences, Linköping University, Linköping, Sweden

**CONTACT** Brynja Ingadottir brynjain@landspitali.is

© 2017 Brynja Ingadottir, Sigridur Zoëga, Katrin Blöndal, David Thue, Ingela Thylen, and Tiny Jaarsma. Published with license by Taylor & Francis.

This is an Open Access article distributed under the terms of the Creative Commons Attribution License (http://creativecommons.org/licenses/by/3.0/), which permits unrestricted use, distribution, and reproduction in any medium, provided the original work is properly cited. The moral rights of the named author(s) have been asserted.

Introduction/Aim: To describe the evaluation of a serious game designed for patients to learn about post-operative pain management.

Methods: The game was developed by an interdisciplinary team. In the game, the player controls the actions of a virtual human character who has recently been discharged home from hospital after surgery. By making different decisions about the character’s daily activities (including pain management) players can observe how their decisions influence the character’s recovery. The usability and efficacy of the game were evaluated in one session with questionnaires measuring usability, attitudes towards pain management and knowledge acquisition; semi-structured interviews; and direct observation while participants played the game.

Results: Participants (N = 20, mean age 48 ± 14, 11 women), recruited from the public, described the usability of the game as high and expressed satisfaction with this novel method of learning, despite some technological challenges. Ease of use was confirmed by observation. Knowledge of pain medication and strategies such as taking pain medication regularly, improved after playing the game. Correct answers increased from 54% (before playing) to 71% (p = 0.001).

Discussion/Conclusions: A serious computer game has the potential to improve knowledge about postoperative pain management. The game was well received by participants and can be a useful tool to initiate and facilitate discussions between healthcare providers and patients.

## Feasibility of two self-guided web-based interventions for adolescents and young adults with migraine: a pilot randomized controlled trial

A. Huguet^a,^^b^, S. Rozario^a^, V. Varalli^a^, S. MacIntyre^a^, P. J. McGrath^a,^^b,^^c^, A. Purdy^d^, I. Kronish^e^, L. Wozney 0000-0001-6933-9596^a^, C. MacLean^f^, J. Stinson^g,^^h^

^a^Centre for Research in Family Health, IWK Health Centre, Halifax, NS, Canada; ^b^Department of Community Health and Epidemiology, Dalhousie University, Halifax, NS, Canada; ^c^Department of Science, Pediatrics, and Psychiatry, Dalhousie University, Halifax, NS, Canada; ^d^Department of Neurology, QE II Health Sciences Centre, Halifax, NS, Canada; ^e^Department of Medicine, Columbia University, New York, NY, USA; ^f^Faculty, Academic Family Medicine, University of Saskatchewan, Saskatoon, SK, Canada; ^g^Anesthesia and Pain Medicine, The Hospital for Sick Children, Toronto, ON, Canada; ^h^Centre for the Study of Pain, University of Toronto, Toronto, ON, Canada

**CONTACT** A. Huguetanna.huguet@dal.ca

© 2017 A. Huguet, S. Rozario, V. Varalli, S. MacIntyre, P. J. McGrath, A. Purdy, I. Kronish, L. Wozney, C. MacLean, and J. Stinson. Published with license by Taylor & Francis.

This is an Open Access article distributed under the terms of the Creative Commons Attribution License (http://creativecommons.org/licenses/by/3.0/), which permits unrestricted use, distribution, and reproduction in any medium, provided the original work is properly cited. The moral rights of the named author(s) have been asserted.

*Aims*: Migraines are common. Cognitive Behavioral Therapy (CBT) is advocated as the first-line treatment although rarely available. Internet-based interventions can help gain access. There is limited evidence that Internet-based CBT, with no human support, are well used, accepted and effective. Our purpose was to assess acceptability and adherence to two self-guided CBT: SPHERE, a comprehensive program aimed to teach a variety of skills, and PRISM, a targeted program aimed to identify headache triggers and provide recommendations to cope with them.

*Method*: A pilot randomized controlled trial was performed. Sixty participants were stratified into two age groups (14-21, 22-35) and randomly allocated to SPHERE, PRISM or usual care. The Client Satisfaction Questionnaire (CSQ-8) and interviews were conducted 4-month post-randomization.

*Results*: Satisfaction was favorable: CSQ-8: SPHERE Mdn=25, range =16-31; PRISM Mdn=24, range =15-30. Adherence was low: 7 of 19 SPHERE participants went beyond the 6th topic out of 30, and PRISM identified a potential headache trigger and provided recommendations to 6 out of 20 participants.

*Conclusions*: Low adherence to internet interventions is common. The nascent field of research on internet interventions could benefit from taking into account participant views. Based on participants’ feedback we are introducing changes (e.g., add instructions, simplify programs) and we will test adherence to new versions.

## Strengthening system services and supports to promote stay-at-work and return-to-work for persons living with persistent pain: A qualitative study

Lynn Cooper^a^, Lynn Shaw^b^, Maria Bryson-Campbell^c^, and Bill Chedore^d^

^a^Canadian Pain Coalition, Oshawa, Ontario, Canada; ^b^School of Occupational Therapy, Faculty of Health Professions, Dalhousie University, Halifax, Nova Scotia, Canada; ^c^Senior Research Associate, ResearchOne, Brights Grove, Ontario, Canada; ^d^Canadian Injured Workers’ Alliance, Thunder Bay, Ontario, Canada

**CONTACT** Lynn Cooper lkcooperbes@rogers.com; office@canadianpaincoalition.ca

© 2017 Lynn Cooper, Lynn Shaw, Mikelle Bryson-Campbell, and Bill Chedore. Published with license by Taylor & Francis.

This is an Open Access article distributed under the terms of the Creative Commons Attribution License (http://creativecommons.org/licenses/by/4.0/), which permits unrestricted use, distribution, and reproduction in any medium, provided the original work is properly cited.

Introduction/Aim: Tapping into the expertise of a broad spectrum of experts that interact with injured workers with chronic pain was identified as an imperative by the steering committee of the *Creating a Way Forward Project* initiated by the Canadian Pain Coalition and Canadian Injured Workers’ Alliance. The aim was to provide evidence and knowledge to inform social change in the opportunities for injured workers to live and work with chronic pain.

Methods: A focus group study comprised of N=15 experts participated in a group interview/discussion process exploring barriers, facilitators and opportunities to work with persistent pain. A template analysis (King 2004) was conducted by two qualitative researchers to identify the preliminary codes from dialogue with a heterogeneous group of experts and stakeholders. Deeper reflection on the discussion from a constructivist standpoint led to core themes and insights that were confirmed with experts.

Results: Insights included: Purposeful capacity building through knowledge and education; enabling interconnectivity of services and providers; establishing collaborative planning processes among people, services and stakeholders; and strengthening the interdependence of the stakeholders through a focus on shared benefits related to achieving positive outcomes for injured workers with chronic pain.

Discussion/Conclusions: Social change at the systems level must consider the functionality, integrity, and stabilization of services and supports in the context in which persons with persistent pain live and work. In the poster we will present opportunities informed by this research to help injured workers with persistent pain engage in orchestrating a healthy way of living, stay at work and return to work.

## Evaluation of the quality and proportion of pain related content of available online health resources for parents of preterm infants receiving intensive care.

Brianna Richardson^a,^^b^, Justine Dol^b,^^c^, Talia Boates^b^, and Mikelle Campbell-Yeo^a,^^b,^^d^

^a^School of Nursing, Faculty of Health Professions, Dalhousie University; ^b^Centre for Pediatric Pain Research, IWK Health Centre; ^c^Faculty of Health Professions, Dalhousie University; ^d^Departments of Pediatrics, Psychology & Neuroscience, Dalhousie University

**CONTACT** Marsha Campbell-Yeo marsha.campbell-yeo@dal.ca

© 2017 Brianna Richardson, Justine Dol, Talia Boates, and Marsha Campbell-Yeo. Published with license by Taylor & Francis.

This is an Open Access article distributed under the terms of the Creative Commons Attribution License (http://creativecommons.org/licenses/by/3.0/), which permits unrestricted use, distribution, and reproduction in any medium, provided the original work is properly cited.

Aims: Parents of preterm infants are increasingly turning to the Internet for information on their baby’s health needs. However, little is known of the the reliability and evidence based up-to-date quality of online information for parents related to general or pain specific care.

Methods: We systematically searched Google.com combining “premature baby” plus 12 key terms (i.e., “pain”, “breastfeeding OR feeding”). Inclusion criteria were webpages published in English within the top 100 ‘hits’ per search on May 28/16. Exclusion criteria included newspaper articles, scientific sources, hospital websites, or non-relevant results. Websites were evaluated using DISCERN, a 16-item questionnaire to evaluate reliability and overall quality and Health On Net (HON) to evaluate credibility and transparency.

Results: The initial search yielded 1,200 websites with 337 eligible for full review. As of March 31/17, 178 websites were reviewed and evaluated with 98 websites excluded. Using DISCERN, only 33.7% of websites received above 80% score on reliability of publication with only 12.5% being rated 5 (out of 5) on overall quality. Findings indicate few websites focused on infant pain (n=8), with only 25.0% receiving a score above 80% in reliability with 25.0% being rated 5 (out of 5) in overall quality. Of pain websites, 25% held HONcode certification (12.9% total) and were dated after 2015 (23.0% total).

Conclusions: This evaluation suggests that infant pain is not a prevalent topic addressed online and the quality of websites that do exist are lacking in reliable, credible, updated content, scoring worse than general websites overall.

## Neural correlates of affective touch in mice

Claire Marie Chan^a^, Chulmin Cho^b^, Sivaani Sivaselvachandran^b^, and Loren J. Martin^b^

^a^Cell & Systems Biology, University of Toronto Mississauga, Mississauga, Ontario, Canada; ^b^Psychology, University of Toronto Mississauga, Toronto, Ontario, Canada

**CONTACT** Claire Marie Chan claire.chan@utoronto.ca

© 2017 Claire Marie Chan, Chulmin Cho, Sivaani Sivaselvachandran, and Loren J. Martin. Published with license by Taylor & Francis.

This is an Open Access article distributed under the terms of the Creative Commons Attribution License (http://creativecommons.org/licenses/by/3.0/), which permits unrestricted use, distribution, and reproduction in any medium, provided the original work is properly cited. The moral rights of the named author(s) have been asserted.

Introduction/Aim: C-tactile (CT) afferents convey affective touch signals and are stimulated via light and slow stroking of hairy skin. It has been suggested that CT afferents become dysfunctional in chronic pain, potentially accounting for clinical reports of pain in response to previously innocuous stimuli. Human studies describe CT projections to a network that includes the insula, amygdala, and prefrontal cortex. However, there is not an analogous network in a mouse model. Activation of CT afferents in mice has been proposed to have analgesic effects; the lack of a mouse network provides a challenge for animal researchers who wish to explore therapeutic benefits of CT activation. Our current work aims to describe an activational network of affective touch in mice.

Methods: To stimulate CT afferents, a “gentle touch” protocol was used: an experimenter applied a stroke to a mouse using a gloved finger, while a non-stroked mouse remained undisturbed to serve as a comparison. Western Blot c-fos analysis was used to compare brain activation between stroked and non-stroked animals. ROIs were chosen based on the human literature.

Results: Preliminary immunoblots revealed: (1) higher relative c-fos expression in the amygdala and hypothalamus of stroked vs. non-stroked animals, and (2) higher relative c-fos expression in the hypothalamus and nucleus accumbens of male stroked animals.

Discussion/Conclusions: This work is the first to demonstrate regional activity in response to CT stimulation in rodents. Further work will map a comprehensive neural profile of affective touch in a rodent model, which can be used to investigate tactile dysfunction in pain.

## T-cell dependent reversal of allodynia in pregnant mice

Sarah Rosen, Boram Ham, Michael Haichin, Shannon Drouin, Jeffrey Mogil

Psychology, McGill University, Montreal, QC, Canada

**CONTACT** Sarah Rosen srosen625@gmail.com

© 2017 Sarah Rosen, Boram Ham, Michael Haichin, Shannon Drouin, and Jeffrey Mogil. Published with license by Taylor & Francis.

This is an Open Access article distributed under the terms of the Creative Commons Attribution License (http://creativecommons.org/licenses/by/3.0/), which permits unrestricted use, distribution, and reproduction in any medium, provided the original work is properly cited. The moral rights of the named author(s) have been asserted.

Introduction/Aim: It has been consistently reported in the clinic that many female chronic pain sufferers have an attenuation of symptoms during pregnancy. Also, rats have an increased acute pain tolerance during pregnancy, due to an increase in opioid receptors in the spinal cord. These past studies, however, did not consider that neurons are not the only cell type involved in pain processing, which also involves cells of the immune system, such as microglia and T-cells. We wished to investigate the implications of this for microglia-dependence of mechanical allodynia in pregnant mice and attenuation of chronic pain during pregnancy.

Methods: Subjects were naïve, young adult female mice. Neuropathic surgery was a unilateral spared nerve injury (SNI), and inflammatory injury was a single unilateral hind paw injection of complete Freund’s adjuvant (CFA 50% in 20 μl) or 5% formalin. Behavioral testing consisted of Von Frey, Hargreaves’ method, tail withdrawal, and formalin percent licks.

Results: Early pregnant mice do switch to a microglia dependent mechanism to mediate allodynia. Female mice with injury progressively lose mechanical allodynia during pregnancy, and is reversible by intrathecal administration of naloxone. Nude pregnant mice do not exhibit a blockade of mechanical allodynia during late pregnancy after injury. Furthermore, naïve nude mice show a blunted analgesic response to opioids, which can be rescued with the adoptive transfer of immune cells from CD-1 mice.

Discussion/Conclusions: This data strongly suggests that T-cells are playing a role in opioid-mediated analgesia. Further experiments are aimed at determining how T-cells affect opioid analgesia produced by pregnancy and morphine administration.

## Music medicine: An alternative treatment for managing suffering associated with temporomandibular disorder

Alicia Howard^a^, Howard Tenenbaum^b^, Michael B. Goldberg^c^, Denise Paneduro^d^, Bruce V. Freeman^e^, Kethmini Amarasinghe^d^, Allan Gordon^d^, and Lee Bartel0000-0002-3925-5292^a^

^a^Faculty of Music, University of Toronto, Toronto, ON, Canada; ^b^Periodontology at the Faculty of Dentistry, University of Toronto and Periodontics at Mount Sinai Hospital, Toronto, ON, Canada; ^c^Department of Periodontics, Faculty of Dentistry, University of Toronto, Toronto, ON, Canada; ^d^The Wasser Pain Management Centre, Mount Sinai Hospital, Toronto, ON, Canada; ^e^Mount Sinai, Facial Pain Unit, Mount Sinai Hospital, Toronto, ON, Canada

**CONTACT** Alicia Howard alicia.howard@mail.utoronto.ca

© 2017 Alicia Howard, Howard Tenenbaum, Michael B. Goldberg, Denise Paneduro, Bruce V. Freeman, Kethmini Amarasinghe, Allan Gordon, and Lee Bartel. Published with license by Taylor & Francis.

This is an Open Access article distributed under the terms of the Creative Commons Attribution License (http://creativecommons.org/licenses/by/3.0/), which permits unrestricted use, distribution, and reproduction in any medium, provided the original work is properly cited. The moral rights of the named author(s) have been asserted.

Introduction/Aim: This study examined the effectiveness of *music medicine* for patients with temporomandibular joint disorder (TMD) using a single blind randomized crossover trial.

Methods: Twenty-five patients with TMD from an outpatient dental clinic were randomly assigned to one of two interventions: 1) vibroacoustic therapy (Sound Oasis VTS-1000) with music and 2) prescribed self-selected music. Participants began the second assigned treatment following a 4-week washout period. Outcome measures included mood (Multi-Dimensional Mood Questionnaire; MMQ), pain severity (Visual Analog Scale), quality of life (Quality of Life Enjoyment and Satisfaction), and depression (CES-D). Outcomes were assessed before and after each treatment. Mixed ANOVA with Order (self-selected music or VAT first), Time of Treatment (First Treatment vs. Second Treatment), and Treatment Effect (Pre-Treatment vs. Post-Treatment) as factors were conducted for analysis of outcomes.

Results: Given the high correlation between anxiety and depression, anxiety and depression scores from the MMQ were combined into a measure of suffering. There was a significant Treatment Effect × Time of Treatment interaction, F(1,23) = 7.68, *p* = .011. A post hoc t-test showed a significant decrease in suffering, (Pre *M* = 4.48, Post *M* = 3.90, *d* = 0.46), t(24) = 3.49, *p* = .002. No significant treatment effects were observed for pain, depression, or quality of life.

Conclusion: The results suggest that music medicine may not reduce the intensity of pain, but it may help TMD patients to better manage the emotional suffering associated with pain.

## Evaluating a sensitive issue: Reliability and validity of allodynia measures used in the somatosensory rehabilitation method

Tara Packham^a^, Claude J. Spicher^b^, and Joy C. MacDermid^c^

^a^Hand Therapy Clinic, Regional Rehabilitation Program, Hamilton Health Sciences, Hamilton, Ontario, Canada; ^b^Medicine Department, University of Fribourg, Fribourg, Fribourg, Switzerland; ^c^School of Rehabilitation Sciences, McMaster University, Hamilton, ON, Canada

**CONTACT** Tara Packham packhamt@hhsc.ca

© 2017 Tara Packham, Claude J. Spicher, and Joy C. MacDermid. Published with license by Taylor & Francis.

This is an Open Access article distributed under the terms of the Creative Commons Attribution License (http://creativecommons.org/licenses/by/3.0/), which permits unrestricted use, distribution, and reproduction in any medium, provided the original work is properly cited. The moral rights of the named author(s) have been asserted.

Introduction/Aim: Somatosensory rehabilitation is a conservative treatment program for the somatosensory changes seen in neuropathic pain syndromes. While several level 4 studies supporting the effectiveness of this method have been published, the psychometric properties of the embedded measurement techniques have not. Monofilaments are used in standardized procedures for mapping (Allodynography) the area of skin that is painful to touch, and quantifying the severity of allodynia (Rainbow Pain Scale). The purpose of this study was to investigate the reliability and validity of Allodynography and the Rainbow Pain Scale.

Methods: We evaluated these clinical examination signs in persons with complex regional pain syndrome (CRPS), or neuropathic pain after nerve injury or fracture. Blinded evaluations were completed by 2 different evaluators at baseline, and again by one of the evaluators one week later. Comparisons were made to the McGill Pain Questionnaire, and other evaluations of pain and disability.

Results: Inter-rater reliability of the Rainbow Pain Scale, including a zero score for no allodynia was ICC= 0.79 in 24 subjects; test-retest reliability was ICC=0.82 (n=18). In the 12 participants identified to have allodynia, inter-rater reliability of allodynography was ICC=0.97; test-retest reliability was ICC=0.89. Convergent and divergent construct validity hypotheses were largely confirmed in both direction and magnitude.

Discussion/Conclusions: This small study of the core evaluations used in the somatosensory rehabilitation method has generated preliminary support for the reliability of these tools. Future work should include replication in larger samples with diverse forms of neuropathic pain and CRPS, and further evaluations of validity and responsiveness.

## Survey of canadian physicians’ educational needs and interests in chronic pain management in cancer patients without an active disease

Kim Horrill^a^, and Jessica Lam^b^

^a^ACNP Purdue Pharma Canada; ^b^Purdue Pharma Canada

**CONTACT** Kim Horrill kim.horrill@purdue.ca

© 2017 Kim Horrill and Jessica Lam. Published with license by Taylor & Francis.

This is an Open Access article distributed under the terms of the Creative Commons Attribution License (http://creativecommons.org/licenses/by/3.0/), which permits unrestricted use, distribution, and reproduction in any medium, provided the original work is properly cited. The moral rights of the named author(s) have been asserted.

Introduction/Aim: The most significant barrier to effective pain management in cancer patients without an active disease is the limited knowledge and skills of many physicians who manage pain. The purpose of this survey was to assess the educational needs and interests of primary care physicians treating chronic pain in the continuum of cancer.

Methods: A survey was mailed out to primary care physicians across Canada. The physician’s demographics, knowledge and confidence with practical management, opinions on strategies to optimize prescribing, goals with chronic pain management, evaluation of barriers and gaps, and topics of interests were collected.

Results: A total of 161 responses were collected. Majority of physicians (90%) acknowledged their lack of sufficient knowledge about chronic pain management in cancer patients without an active disease. Only 10% of the respondents were confident in managing these patients. The three most cited barriers physicians face when managing chronic pain were the coverage of medications, concern about patient opioid misuse or abuse, and patient noncompliance. Physicians were most interested in learning about these three topics: Treatment guidelines; Pharmacological treatment options; and nonpharmacological treatment options for chronic pain which would help improve their ability to manage these patients appropriately. Other topics of interest include pathophysiology of chronic pain in continuum of cancer and pain assessment.

Conclusions: Physicians who manage chronic pain related to cancer treatment in patients without an active disease have knowledge deficiencies. By understanding physicians’ educational needs and interests, relevant strategies can be developed and initiated to close these knowledge gaps.

## Decision-making by sub-elite athletes about playing through pain: Results of a focused ethnography of gymnasts, rowers and speed skaters

Amy Barrette^a^, and Katherine Harman^b^

^a^Exercise Science, Concordia University, Montreal, QC, Canada; ^b^Physiotherapy, Dalhousie University, Halifax, NS, Canada

**CONTACT** Amy Barrette amyfbarrette@gmail.com

© 2017 Amy Barrette and Katherine Harman. Published with license by Taylor & Francis.

This is an Open Access article distributed under the terms of the Creative Commons Attribution License (http://creativecommons.org/licenses/by/3.0/), which permits unrestricted use, distribution, and reproduction in any medium, provided the original work is properly cited. The moral rights of the named author(s) have been asserted.

Introduction/Aim: To explore factors associated with sub-elite athletes playing through pain in gymnastics, rowing and speed skating.

Methods: We conducted semi-structured interviews with athletes, coaches and rehabilitation specialists. Coach participants were recruited through their Provincial Sport Organization. Subsequently, injured athletes of the recruited coaches who were training for a major competition were recruited. Rehabilitation specialists that were known to treat sub-elite athletes were recruited independently by email. We studied: five coaches, four athletes and three rehabilitation specialists. Athletes were photographed during a regular practice shortly before an important competition and all participants were interviewed after that competition. Photographs were used during the interview to stimulate discussion. Interviews were transcribed verbatim and thematic analysis followed using NVivo™ software.

Results: The participant interviews revealed 3 main themes related to playing through pain. They are: Listening to your body, Decision-Making and Who decides. Decision-Making was the dominant theme of our findings. Although there were circumstances described when either the coach or rehabilitation specialist removed an athlete from play, mostly the athletes decided to continue to play through their pain. Different factors were identified to come into play when the athletes were decision-making including: Impact & Consequences, Support & Pressure, Culture of Risk and Dreams & Goals.

Discussion/Conclusions: When sub-elite athletes, striving to be the best in their sport continue to train with the pain of an injury, performance is affected in the short term and long-term consequences are also possible. Our study provides some insight into the contrasting forces that athletes balance as they decide to continue or to stop.

## The levels of serum IGF-1 and IGF-2 in myofascial pain syndrome (MPS)

L. Grosman-Rimon^a^, John Srbely^b^, Lukas Linde^b^, John Flannery^a^, and Dinesh Kumbhare^a^

^a^Department of Medicine, Division of Physical Medicine and Rehabilitation, Toronto Rehabilitation Institute, Toronto, Ontario, Canada; ^b^Human Health and Nutritional Science, University of Guelph, Guelph, Ontario, Canada

**CONTACT** Liza Grosman-Rimon liza.grosman-rimon@uhn.ca

© 2017 Liza Grosman-Rimon, John Srbely, Lukas Linde, John Flannery, and Dinesh Kumbhare. Published with license by Taylor & Francis.

This is an Open Access article distributed under the terms of the Creative Commons Attribution License (http://creativecommons.org/licenses/by/3.0/), which permits unrestricted use, distribution, and reproduction in any medium, provided the original work is properly cited. The moral rights of the named author(s) have been asserted.

Introduction/Aim: Insulin-like growth factor-1 (IGF-1) plays an important role in muscle maintenance and repair. The role of IGF-2 in the muscle is less clear. The objectives of our study were to compare the levels of IGF-1 and IGF-2 in patients with acute myofascial pain syndrome (MPS) versus healthy asymptomatic controls and to examine sex differences.

Methods: Participants were recruited randomly from the hospital emergency department with acute MPS (n = 43) and non-MPS controls (n  =  21), recruited via advertisements in the hospital and community. The group and sex differences between serum IGF-1 and IGF-2 were assessed. IGF-1 and IGF-2 were measured systemically in patients with MPS within 24 hours of symptoms and in healthy controls, using antibody-immobilized beads on a Luminex analyzer.

Results: No significant differences were observed in IGF-1 levels (mean ± SEM, pg/mL) between males and females with MPS (99,446.77 ± 597,0.0, and 79,930.75 ± 576,4.6, respectively) and healthy asymptomatic males and females (91,847.32 ± 669,7.3, and 107,512.40 ± 117,27.8, respectively). The mean IGF-2 levels (mean ± SEM, pg/mL) of men and women with MPS were lower (253,343.00 ± 349,61.8, and 204,524.20 ± 35,610.51, respectively) than those of healthy men and women (428,177.20 ± 284,78.6, and 511,274.4 ± 800,07.6, respectively).

Discussion/Conclusions: IGF-2 was lower in patients with acute MPS versus healthy asymptomatic controls. No differences were observed between males and females in the groups. Future studies should investigate the mechanisms and the role of IGF-2 in muscle maintenance and repair in MPS.

## Are there sex differences in the levels of inflammatory mediators in patients with acute myofascial pain?

Liza Grosman-Rimon^a^, John Srbely^b^, Lukas Linde^b^, John Flannery^a^, and Dinesh Kumbhare^a^

^a^Department of Medicine, Division of Physical Medicine and Rehabilitation, Toronto Rehabilitation Institute, Toronto, Ontario, Canada; ^b^Human Health and Nutritional Science, University of Guelph, Guelph, Ontario, Canada

**CONTACT** Liza Grosman-Rimon liza.grosman-rimon@uhn.ca

© Liza Grosman-Rimon, John Srbely, Lukas Linde, John Flannery, and Dinesh Kumbhare. Published with license by Taylor & Francis.

This is an Open Access article distributed under the terms of the Creative Commons Attribution License (http://creativecommons.org/licenses/by/3.0/), which permits unrestricted use, distribution, and reproduction in any medium, provided the original work is properly cited. The moral rights of the named author(s) have been asserted.

Introduction/Aim: A recent study reported that sex and gender differences in pain and analgesia exist. A higher prevalence for women than men were reported to have several clinical pain-related disorders. Emerging evidence suggests that inflammatory mediators may contribute to sex differences in pain sensitivity, involving not only central but also peripheral pathways. The aim of the study is to compare the levels of inflammatory mediators between men and women with acute myofascial pain.

Methods: The differences between the levels of systemic inflammatory mediators between women (n = 33) and men (n = 24) with MPS and healthy women (n = 12) and men (n = 19) were assessed.

Inflammatory mediators include macrophage inflammatory proteins (MIP-1α), MIP-1β, macrophage-derived chemokine (MDC), MCP-1, granulocyte-macrophage colony-stimulating factor (GM-CSF), tumor necrosis factor-α (TNF-α), Interleukin-6 (IL-6), IL-8, and growth factors fibroblast growth factor (FGF-2), Vascular endothelial growth factor (VGEF), and platelet-derived growth factor (PDGF) and were measured in patients with MPS within 24 hours of symptoms and in healthy controls, using antibody-immobilized beads on a Luminex analyzer.

Results: The levels of the inflammatory mediators and growth factors were significantly higher in patients with MPS compared to healthy controls. No significant sex differences were observed in the MPS group in the levels of TNF-a, MCP-1 IL8, IL-6, MIP-1a and IL-1a, FGF-2, PDGF. Similarly, in healthy controls, no sex differences were found in TNF-a, MCP-1, IL-8, IL-6, MIP-1a, MIP-1b, IL-1a and GM-CSF, as well as, PDGF levels. Median G-CSF levels were significantly higher in men with MPS compared to women with MPS, while no sex differences were found in healthy controls. In men compared to women with MPS, there was a trend of higher median levels of GM-CSF, MIP-1β, and VGEF. Pain scores did not correlate with the levels of inflammatory mediators in patients with MPS.

Discussion/Conclusions: The levels of most of the inflammatory mediators assessed in this study were elevated to a similar extent in both men and women. Investigating biomarker levels in MPS may be important for both diagnosis and treatment of MPS.

## Cross-border trafficking of prescription oxycodone products manufactured in Canada

K. Patrick May^a^, Travis Rosen^a^, Colleen M. Haynes^a^, Nabarun Dasgupta^a^, Jody L. Green^b^, Richard C. Dart^b^, and Janetta L. Iwanicki^b^

^a^Rocky Mountain Poison and Drug Center, Denver Health and Hospital Authority, Denver, CO, USA; ^b^Canadian Consumer Product and Pharmaceutical Safety Inc., Toronto, ON, Canada

**CONTACT** K. Patrick May kevin.may@rmpdc.org

© 2017 Denver Health and Hospital Authority. Published with license by Taylor & Francis.

This is an Open Access article distributed under the terms of the Creative Commons Attribution License (http://creativecommons.org/licenses/by/3.0/), which permits unrestricted use, distribution, and reproduction in any medium, provided the original work is properly cited. The moral rights of the named author(s) have been asserted.

Introduction/Aim: As more restrictions are imposed on prescription opioid access, diversion of products across the United States-Canada borders is likely. The StreetRx Program was studied to quantify this cross-border trafficking of prescription oxycodone products manufactured in Canada and used in the United States (US).

Methods: StreetRx is a publicly accessible website which collects anonymously reported street price data for prescription and illicit drugs. US site users reporting acquisition of certain Canadian products were prompted to identify the country in which they acquired the product. Drugs included oxycodone products manufactured only in Canada. This report includes data collected from August to November 2016.

Results: There were 67 reports of Canadian prescription oxycodone products reported to the US StreetRx website; 51 (76%) of these reports confirmed that they selected a drug that is marketed in Canada but was acquired in the US, 9 (13%) indicated that the drug was acquired in Canada and brought into the US, and 7 (10%) did not report the country in which the drug was acquired. There were 62 (92.5%) reports of crushable oxycodone products and 5 (7.5%) reports of OxyNEO®, the only difficult to crush (tamper-resistant) oxycodone product in our analysis. Reports in the US came from diverse geographic areas and were not concentrated in states bordering Canada.

Discussion/Conclusions: Illicit trade of prescription oxycodone products from Canada to the US does occur. Further monitoring and research is needed to examine the driving factors and extent to which this illicit trade persists.

## Charting knowledge into practice: assessing opioid and chronic pain management performance in ECHO ontario, a retrospective chart review

Jane Zhao, MSc^a^, Bayley Ostenfeldt, MPH^b^, Bryan MacLeod, MD^c^, Leslie Carlin, PhD^d^, and Andrea Furlan, MD PhD ^a,^^d^

^a^ECHO Ontario, MSK Outpatient Department, Toronto Rehabilitation Institute, University Health Network, Toronto, Ontario, Canada; ^b^Pain Research, CERAH, Lakehead University, Thunder Bay, Ontario, Canada; ^c^Northern Ontario School of Medicine, Lakehead University, Thunder Bay, Ontario, Canada; ^e^Department of Medicine, University of Toronto, Toronto, Ontario, Canada

**CONTACT** Jane Zhao jane.zhao@uhn.ca

© 2017 CIHR.

This is an Open Access article distributed under the terms of the Creative Commons Attribution License (http://creativecommons.org/licenses/by/3.0/), which permits unrestricted use, distribution, and reproduction in any medium, provided the original work is properly cited. The moral rights of the named author(s) have been asserted.

Introduction and aim: Opioid and chronic pain management is a challenging and complex problem. ECHO (Extension for Community Healthcare Outcomes) Ontario Chronic Pain and Opioid Stewardship (ECHO) is a telemedicine program designed to educate health care providers, improve pain management, and disseminate best practices. Though previous studies suggest that ECHO can be an effective strategy for knowledge translation, few studies have explored provider performance in clinical practice. This study evaluates the translation of knowledge from ECHO and its implementation into practice.

Methods: A chart review tool based on Moore’s framework for Continuing Medical Education was used to assess provider performance one year prior to (‘baseline’) and one year after attending ECHO. Providers were recruited from both urban and rural practice settings. Patient charts were eligible if they had been prescribed opioids at any time during the period of assessment.

Results: Compared to baseline, the one-year assessment demonstrated that providers increased appropriate use of urine drug screens, signed opioid contracts, documentation of risk assessment tools, and documentation of adverse events. Some providers switched their patients from short-acting to long-acting opioids; others did not document change in their opioid prescribing patterns. Change in non-pharmacological modalities for chronic pain management varied depending on the geographical location of the practice.

Discussion and conclusion: Our results demonstrate the documented implementation of knowledge into clinical practice at one-year following ECHO attendance. This finding complements previous findings of increased self-reported knowledge and self-efficacy in chronic pain management. Further work aims to refine the chart review tool’s sensitivity to change and to investigate patient-level outcomes.

